# Bistable Switching of π‐Conjugation in a Dithienylethene‐Bridged Luminescent Trityl Diradicaloid

**DOI:** 10.1002/anie.5705848

**Published:** 2026-09-04

**Authors:** Xingmao Chang, Sören Schlittenhardt, Julia Zolg, Maximilian Herm, Siyang He, Vivek Chandrakant Wakchaure, Dinar Abdullin, Robert Göstl, Dirk M. Guldi, Alexander J. C. Kuehne, Sabine Richert, Max von Delius

**Affiliations:** ^1^ Institute of Organic Chemistry and Center for Integrated Quantum Science and Technology Ulm University Ulm Germany; ^2^ Institute of Physical Chemistry II Ulm University Ulm Germany; ^3^ Institute of Physical and Theoretical Chemistry Johann Wolfgang Goethe‐University Frankfurt Frankfurt Germany; ^4^ Institute of Macromolecular and Organic Chemistry and Center for Integrated Quantum Science and Technology Ulm University Ulm Germany; ^5^ FAU Profile Center Solar Department of Chemistry and Pharmacy & Interdisciplinary Center for Molecular Materials (ICMM) Friedrich‐Alexander‐Universität Erlangen‐Nürnberg Erlangen Germany; ^6^ DWI – Leibniz Institute for Interactive Materials Aachen Germany; ^7^ Institute of Technical and Macromolecular Chemistry RWTH Aachen University Aachen Germany; ^8^ Department of Chemistry Indian Institute of Technology Jodhpur Karwar, Rajasthan India; ^9^ Department of Chemistry and Biology University of Wuppertal Wuppertal Germany

**Keywords:** conjugated materials, EPR spectroscopy, persistent diradicals, photoswitches

## Abstract

Robust external manipulation of spin communication in molecular frameworks is desirable for potential applications in quantum information science. Herein, we incorporate two types of dithienylethene (DTE) units into tris(2,4,6‐trichlorophenyl)methyl (TTM)‐based diradicaloids and report on the successful bistable switching of the optical and magnetic properties of the molecule. Visible‐light irradiation of the open form generates up to 97% of the closed form, in which conjugation between the two radical centers leads to absorption over the entire visible range of the spectrum as well as a twofold increase of the singlet–triplet gap. These results establish TTM/DTE hybrids as persistent and multi‐level responsive diradicaloids.

## Graphical abstract

Photoswitches that affect π‐conjugation offer a powerful strategy for external manipulation of luminescent diradicals. Dithienylethene‐linked persistent trityl diradicaloids, **DTE‐TTM_2_** and **F_6_‐DTE‐TTM_2_**, exhibit bistable switching of their optical, electrochemical and magnetic properties.

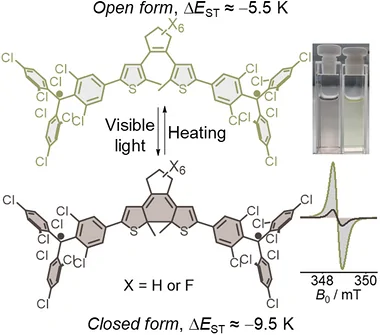

## 1 Introduction

Diradical(oid)s, possessing two unpaired electrons with varying degrees of spin–spin interactions, have attracted considerable interest because of their electronic structure, optical properties, and complex spin states, which differ markedly from those of monoradicals, and promise applications in quantum sensing and communication [1, 2, 3, 4, 5, 6, 7, 8, 9, 10, 11, 12, 13, 14]. Switchable diradicals provide a molecular platform for the reversible optical/thermal control of electronic spin states [9, 15]. Pioneering work by Irie et al. appended nitronyl nitroxide radicals to diarylethene‐based photoswitches (Figure 1a), enabling reversible modulation of the intramolecular magnetic interaction by light irradiation [16, 17, 18, 19]. More recently, switching between open‐shell and closed‐shell electronic states has been achieved by the groups of Dumele, Juríček, Kubo, and Feringa [20, 21, 22, 23, 24].

**FIGURE 1:**
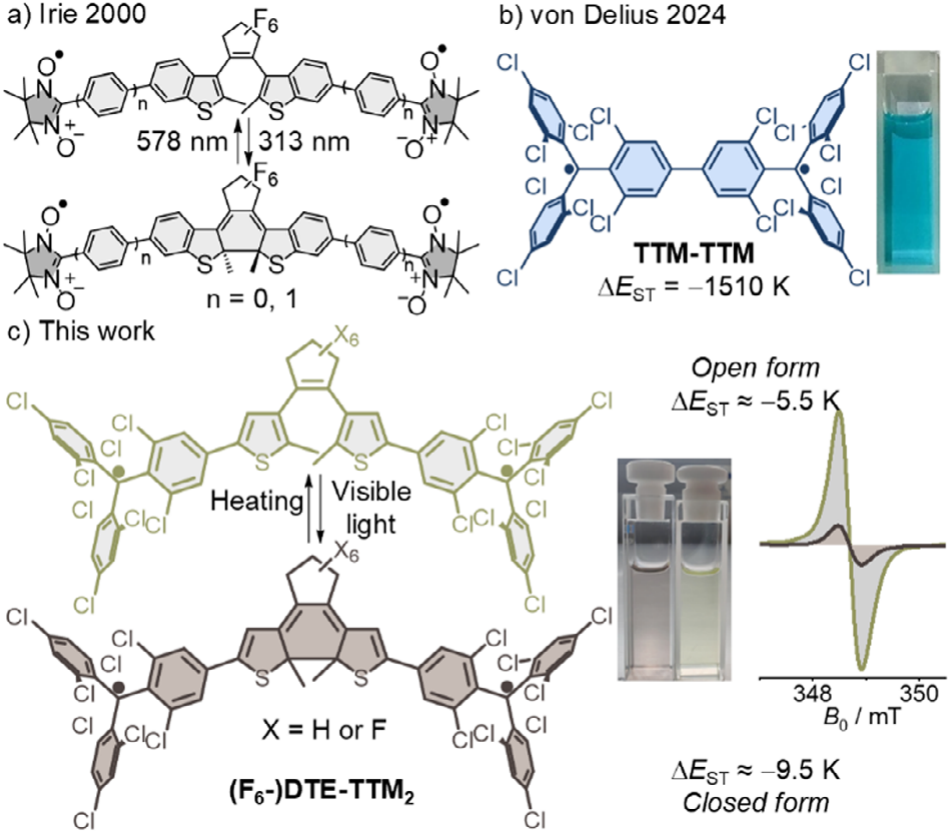
(a and b) Most relevant prior work on photoswitchable DTE‐nitronyl nitroxide and Chichibabin‐type **TTM‐TTM** diradicaloids. (c) Bistable switching demonstrated in this work and indication of optical and magnetic response.

Kinetically stabilized trityl radicals, such as perchlorotriphenylmethyl (PTM) and tris(2,4,6‐trichlorophenyl) methyl (TTM) [25, 26, 27, 28, 29, 30, 31], are persistent for months to years at room temperature and have been widely employed as building blocks of multi‐radical architectures [32, 33, 34, 35, 36, 37, 38, 39, 40, 41]. Our serendipitous discovery of a stable Chichibabin diradicaloid (Figure 1b), which exhibits a sky‐blue color arising from extended π‐conjugation [42], in contrast to the corresponding Thiele's and Müller's diradicaloids [43, 44, 45, 46, 47, 48], highlighted the role of the conjugated bridge in modulating optical and magnetic properties [48, 49]. We, therefore, hypothesized that the well‐established diarylethenes [50, 51, 52, 53, 54] as molecular switches that strongly affect conjugation [55], while leaving molecular geometry more or less intact [50, 51, 52, 56, 57, 58], could be ideally suited to manipulate the properties of TTM‐based diradical(oid)s through external stimuli.

To test this hypothesis, we linked two TTM radicals with (fluorinated) dithienylethene bridges to afford two diradicaloids, **DTE‐TTM_2_** and **F_6_‐DTE‐TTM_2_**. These compounds do not only exhibit high diradical character, consistent with the previously reported persistent Chichibabin diradicaloids [42, 59], but they also display bistable switching behavior in their optical properties, particularly a visible color change from pale green to purple for **DTE‐TTM_2_** and yellow to purple for **F_6_‐DTE‐TTM_2_** (Figure 1c), as well as in their electrochemical and magnetic properties upon visible‐light irradiation and heating.

## 2 Results and Discussion

### 2.1 Synthesis and Characterization

**DTE‐TTM_2_** and **F_6_‐DTE‐TTM_2_** were synthesized in their open (“o”) form via Suzuki coupling of the monoradical **TTM‐I** [60] with **o‐(F_6_‐)DTE‐Bpin_2_**, the latter obtained by borylation of the corresponding precursors [56, 61]. The paramagnetic diradical compounds **o‐DTE‐TTM_2_** and **o‐F_6_‐DTE‐TTM_2_** were rigorously purified and characterized using a previously optimized [59] combination of recycling GPC, HPLC, and high‐resolution MALDI‐MS (Figures 2a,b and S1–S4). The single‐crystal x‐ray diffraction (SCXRD) analysis of **o‐F_6_‐DTE‐TTM_2_** confirmed its open‐form structure with the two trityl radical centers adopting a propeller‐like structure around a trigonal sp^2^ carbon atom (Figures 2d and S5). The distance between the two radical centers of 17.4 Å is in agreement with the DFT calculations (Figure S37). The tilted thiophene moieties indicate the typical lack of extended π‐conjugation in the open form of the **F_6_‐DTE** unit. While the DFT‐optimized structure of the closed form **c‐F_6_‐DTE‐TTM_2_** shows a similar distance between radical centers (17.1 Å), all bond lengths and angles are indicative of conjugation along the entire carbon framework connecting the two radical centers (Figures S36–S41).

**FIGURE 2:**
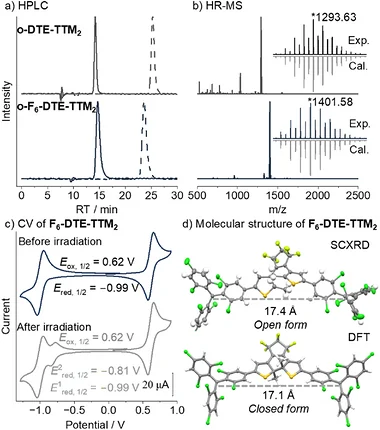
(a) Representative HPLC chromatograms of purified **o‐DTE‐TTM_2_** and **o‐F_6_‐DTE‐TTM_2_** (Buckyprep M, 0.5 mL/min, mobile phase: hexane/toluene, 8:2 for solid lines and 9.5:0.5 for dashed lines). (b) Representative high‐resolution matrix‐assisted laser desorption/ionization‐mass spectrometry (MALDI‐MS; positive mode) of **DTE‐TTM_2_** and **F_6_‐DTE‐TTM_2_**, including isotopic patterns. (c) Cyclic voltammograms (CV) of **F_6_‐DTE‐TTM_2_** (open form; dark blue) and irradiated **F_6_‐DTE‐TTM_2_** (containing ca. 87% of the closed form; grey) at a scan rate of 100 mV/s (0.1 M tetrabutylammonium hexafluorophosphate in anhydrous CH_2_Cl_2_). (d) Molecular structure and inter‐radical distance in the open and closed forms of **F_6_‐DTE‐TTM_2_** derived from single‐crystal x‐ray diffraction (SCXRD) and DFT‐optimized structure, respectively. Crystals were grown from CH_2_Cl_2_ by slow diffusion of antisolvent hexane; CCDC number: 2556067.

The electrochemical properties of the two compounds were investigated by cyclic voltammetry (CV; Figures 2c and S7–S10). **o‐F_6_‐DTE‐TTM_2_** exhibits one reversible oxidation and reduction at 0.62 and –0.99 V, respectively (vs. Fc^+^/Fc). This result indicates the presence of two electrochemically equivalent TTM radical units in the open form. **o‐DTE‐TTM_2_** displays similar oxidations and reductions, but a slightly broader oxidation (Figure S7). Overall, the CV profiles of both open‐form diradicaloids indicate weak electronic communication between the two radical centers, consistent with limited π‐conjugation due to the open form of the DTE bridge. Upon irradiation with light (430 nm), new redox features emerge in both compounds. For irradiated **F_6_‐DTE‐TTM_2_**, a new reduction appears at −0.81 V, while the oxidation remains largely identical to that of **o‐F_6_‐DTE‐TTM_2_** (Figure S10). In contrast, irradiated **DTE‐TTM_2_** exhibits two reductions and three oxidations, when compared with **o‐DTE‐TTM_2_** (Figures S7 and S9). Among these, the oxidation at 0.22 V is likely arising from the closed form of DTE [62, 63]. These characteristic changes in the CV profiles reveal enhanced π‐conjugation along with stronger electrochemical communication between the two radical centers in the closed form.

The optical properties of **o‐DTE‐TTM_2_** and **o‐F_6_‐DTE‐TTM_2_** were first examined by UV–vis absorption and photoluminescence spectroscopy (Figures S12–S14). Both compounds display intense absorption at 377 and 430 nm, assigned to the TTM radical units and the (F_6_‐)DTE moieties, respectively. Relatively weak and broad absorption extending from 510 to 740 nm for **o‐DTE‐TTM_2_** and from 510  to 700 nm for **o‐F_6_‐DTE‐TTM_2_** are also observed. Based on frontier molecular orbital analysis (Figures S44 and S47), these two low‐energy bands are assigned to charge transfer (CT) transitions from the HOMO, which is localized on the electron donor DTE moiety, to the electron acceptor SUMOs (singly unoccupied molecular orbital), situated on the TTM units. Both diradicaloids show weak red emission, presumably originating from the CT excited state, with low photoluminescence quantum yields of 1.0% for **o‐DTE‐TTM_2_** and 0.7% for **o‐F_6_‐DTE‐TTM_2_**. The emission spectra exhibit a maximum at ∼710 nm with a shoulder at ∼800 nm (Figure S14).

### 2.2 Switching of Optical Properties

The photoswitching of the diradicaloids was subsequently examined using LEDs of different wavelengths. Upon irradiation at 365, 430, 465, 490, or 525 nm, the solution color changed visibly from pale green to dark purple, indicating open‐to‐closed form photoisomerization. LED light with a maximum at 430 nm (lamp power: 70 mW) was selected for detailed studies. Upon irradiation of **o‐DTE‐TTM_2_**, new absorption bands at 505 and 710 nm, together with a shoulder at 780 nm, gradually intensified, while the bands at 377 and 430 nm decreased (Figure S19a). Similarly, **F_6_‐DTE‐TTM_2_** showed the gradual growth of new absorption bands at 520 and 790 nm, with a shoulder at 710 nm, accompanied by a decrease in the absorbance between 370 and 450 nm (Figure 3a). Clear isosbestic points were observed at 465 nm for **DTE‐TTM_2_** and at 453 nm for **F_6_‐DTE‐TTM_2_**, indicating a clean two‐state photoisomerization process between the open and closed forms. The intense absorption across the whole visible spectrum, with red‐shifted bands at 710 nm for **c‐DTE‐TTM_2_** and 790 nm for **c‐F_6_‐DTE‐TTM_2_**, suggests enhanced π‐conjugation along the whole scaffold connecting the two TTM radical centers after photocyclization. This enhanced π‐conjugation is further corroborated by frontier orbital analysis, which reveals decreased HOMO–SUMO energy gaps for the closed‐forms, alongside complete orbital delocalization across the entire molecular backbone (Figures S45 and S48).

**FIGURE 3:**
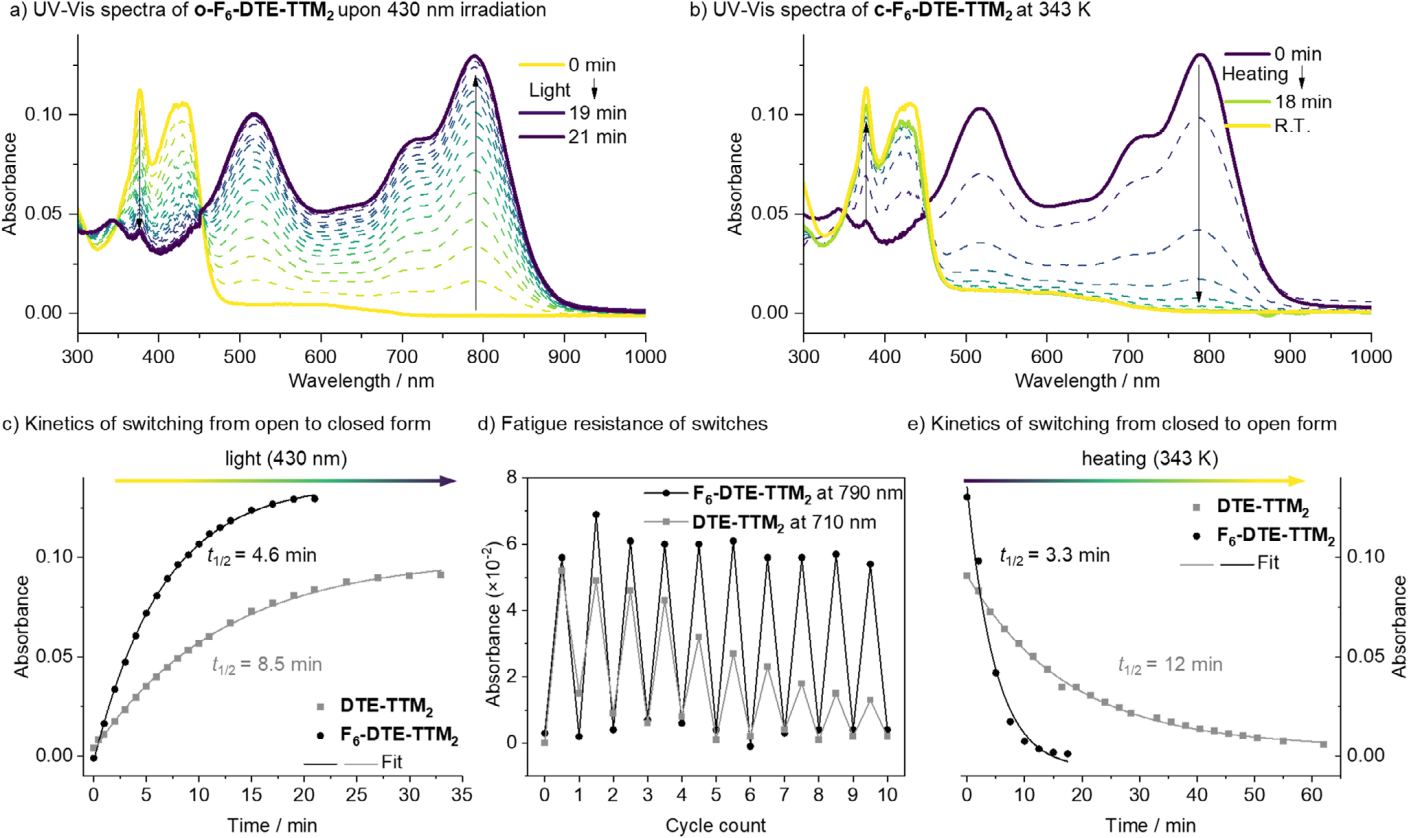
UV–vis absorption spectra of **o‐F_6_‐DTE‐TTM_2_** upon irradiation at 430 nm at room temperature (a) and **c‐F_6_‐DTE‐TTM_2_** upon heating to 343 K (b), recorded at a concentration of 2.5 µM in toluene. Kinetics and fitting of **DTE‐TTM_2_** and **F_6_‐DTE‐TTM_2_** for the open‐to‐closed at room temperature (c) and closed‐to‐open (e) isomerization processes, (d) fatigue resistance of both switches at a concentration of 1.25 µM in toluene with absorbance monitored at 710 nm for **DTE‐TTM_2_** (grey) and 790 nm for **F_6_‐DTE‐TTM_2_** (black).

The reverse switching from the closed to the open form was initially investigated by irradiating the closed‐form diradicaloids with LEDs matching the newly generated absorption bands at 510 and 720 nm. However, the absorption spectra of both solutions remained essentially unchanged in toluene and CCl_4_ (as a heavy‐atom containing solvent), suggesting that irradiation at these wavelengths provides insufficient access to a reactive excited‐state pathway for the closed‐to‐open isomerization [54, 64, 65, 66, 67]. To further elucidate the behavior of **F_6_‐DTE‐TTM_2_**, fs‐transient absorption spectroscopy was performed. Upon photo‐excitation with 420 nm of the **o‐F_6_‐DTE‐TTM_2_**, two states are populated sequentially with lifetimes of 8 ps and 1.2 ns (Figure S17). Albeit 420 nm photo‐excitation experiments afford the closed‐form in the steady‐state experiments, a thermally activated back‐switching throughout the measurements and/or low quantum yields of open‐to‐closed form photoisomerization hampered an unambiguous detection toward the end of the fs‐transient absorption measurements. Upon photoexcitation with 780 nm, the **c‐F_6_‐DTE‐TTM_2_** shows an even faster relaxation via two distinct states with lifetimes as short as 0.4 and 11 ps (Figure S18). It is therefore safe to conclude that the **c‐F_6_‐DTE‐TTM_2_** relaxes to the ground‐state on a timescale that excludes any productive photoisomerization. As such, a thermally activated pathway emerges for the **c‐F_6_‐DTE‐TTM_2_** as the only route to regenerate the **o‐F_6_‐DTE‐TTM_2_**.

Thermal stimulation was therefore employed to drive the reverse switching process. Upon heating to 343 K, both closed‐form solutions gradually returned to their pale‐green (**o‐DTE‐TTM_2_**) and yellow (**o‐F_6_‐DTE‐TTM_2_**) color, consistent with regeneration of the open forms. For **c‐F_6_‐DTE‐TTM_2_**, heating led to a gradual decrease in the absorption at 790 and 520 nm and a concomitant increase in the absorptions at 377 and 430 nm, with a clear isosbestic point at 452 nm (Figure 3b). These changes mirror those observed during photocyclization and, thus, confirm thermally controlled closed‐to‐open switching. **c‐DTE‐TTM_2_** showed similar thermal switching behavior, but required more than 1 h heating at 343 K to reach a plateau, substantially longer than the 18 min observed for **c‐F_6_‐DTE‐TTM_2_** (Figures 3c,e and S19–S21). The switching kinetics of the two diradicaloids in both directions were analyzed by monitoring the absorption changes at 710 nm for **DTE‐TTM_2_** and 790 nm for **F_6_‐DTE‐TTM_2_** (Figure 3c,e). Under identical blue‐light irradiation, **F_6_‐DTE‐TTM_2_** reached a photostationary plateau within ∼20 min, faster than **DTE‐TTM_2_**, which required ∼35 min. The corresponding half‐lives for the open‐to‐closed conversion are 4.6 and 8.5 min, respectively. For the thermally induced closed‐to‐open conversion at 343 K, **c‐F_6_‐DTE‐TTM_2_** showed a half‐life of 3.3 min, whereas **c‐DTE‐TTM_2_** exhibited a much longer half‐life of 12 min. Moreover, **c‐F_6_‐DTE‐TTM_2_** and **c‐DTE‐TTM_2_** show relatively short half‐lives of 4.3 and 42 h at room temperature [68], respectively, which could be beneficial for applications at lower temperatures (Figures S22, S23, and Table 1). Overall, the fluorinated diradicaloid displays faster switching kinetics in both directions, when compared to its non‐fluorinated analogue.

**TABLE 1:** Photophysical, magnetic, and switching properties of the diradicaloids.

Compound		*λ*_abs_ (nm)	*λ*_PL_ (nm)	Δ*E*_ST_ (K)	PSD^a^	Half‐lives
DTE‐TTM_2_	Open	377, 440, 630	710, 800	−(5.6 ± 0.3)	84%	8.5 min^b^
	Closed	502, 710, 780	n.d.	−(9.2 ± 1.0)		42 h^c^
F_6_‐DTE‐TTM_2_	Open	377, 427, 607	710, 800	−(5.5 ± 0.4)	97%	4.6 min^b^
	Closed	520, 710, 790	n.d.	−(9.7 ± 0.8)		4.3 h^c^

^a^ Photostationary distribution (irradiated with 430 nm light and monitored at 710 nm for **DTE‐TTM_2_** and 790 nm for **F_6_‐DTE‐TTM_2_**).

^b^ Photochemical “open‐to‐closed” half‐life upon irradiation with 430 nm light (2.5 µM in toluene).

^c^ Thermal “closed‐to‐open” half‐life at room temperature (2.5 µM in toluene).

The fatigue resistance of both switches was evaluated over repeated irradiation/heating cycles. Each cycle involved irradiation of the open‐form solutions with light (430 nm, 12 W) of identical intensity for 5 min, followed by thermal recovery (343 K) for 20 min for **c‐F_6_‐DTE‐TTM_2_** and 60 min for **c‐DTE‐TTM_2_**. We found that **F_6_‐DTE‐TTM_2_** retained robust switching performance over 10 cycles, with only a slight decrease in the 790 nm absorption. In stark contrast, **DTE‐TTM_2_** showed an approximately 75% decrease in the 710 nm absorption band after 10 cycles, indicating poorer fatigue resistance, in agreement with earlier reports on DTE switches [69]. The photostationary state distribution (PSD) of the two diradicaloids was estimated by comparing the absorption‐intensity ratios of the irradiated samples with those of the pure closed‐form compounds (Section 7.4 in the SI), providing closed/open PSD values of 84% for **DTE‐TTM_2_** and 97% for **F_6_‐DTE‐TTM_2_** (Table 1), which are in the typical range for these two types of switches [57, 58, 69].

### 2.3 Switching of Magnetic Properties

Complementary to the absorption studies, room‐temperature continuous‐wave EPR spectroscopy was used to follow the open‐to‐closed photoisomerization under light irradiation and the consequent closed‐to‐open thermal recovery upon heating (Figures 4a, S26, and S27). The initial solutions of the open‐form diradicaloids revealed the typical EPR spectrum of TTM [42]. Upon irradiation (430 nm, 12 W), both diradicaloids showed a decrease in EPR intensity by ∼80%, while the overall spectral shape remained nearly unchanged, suggesting that the EPR signals before and after irradiation arise from the same paramagnetic species. Subsequent heating of the irradiated solutions showed the opposite trend, with an increase of EPR intensities, which rules out photodegradation as the origin of the signal decrease. In addition, the EPR signal intensity of **c‐F_6_‐DTE‐TTM_2_** is found to increase linearly with concentration, while the spectral line shape remains unchanged, suggesting negligible aggregation effects over the concentration range of 10–300 µM (Figure S29). **F_6_‐DTE‐TTM_2_** restored to the original EPR intensity fully within 10 min, while only 77% of the initial EPR intensity was recovered for **DTE‐TTM_2_** within 80 min and this process was significantly slower than for **F_6_‐DTE‐TTM_2_**. These results agree well with the optical switching kinetics and demonstrate that **F_6_‐DTE‐TTM_2_** undergoes faster and more robust reversible magnetic switching than **DTE‐TTM_2_**.

**FIGURE 4:**
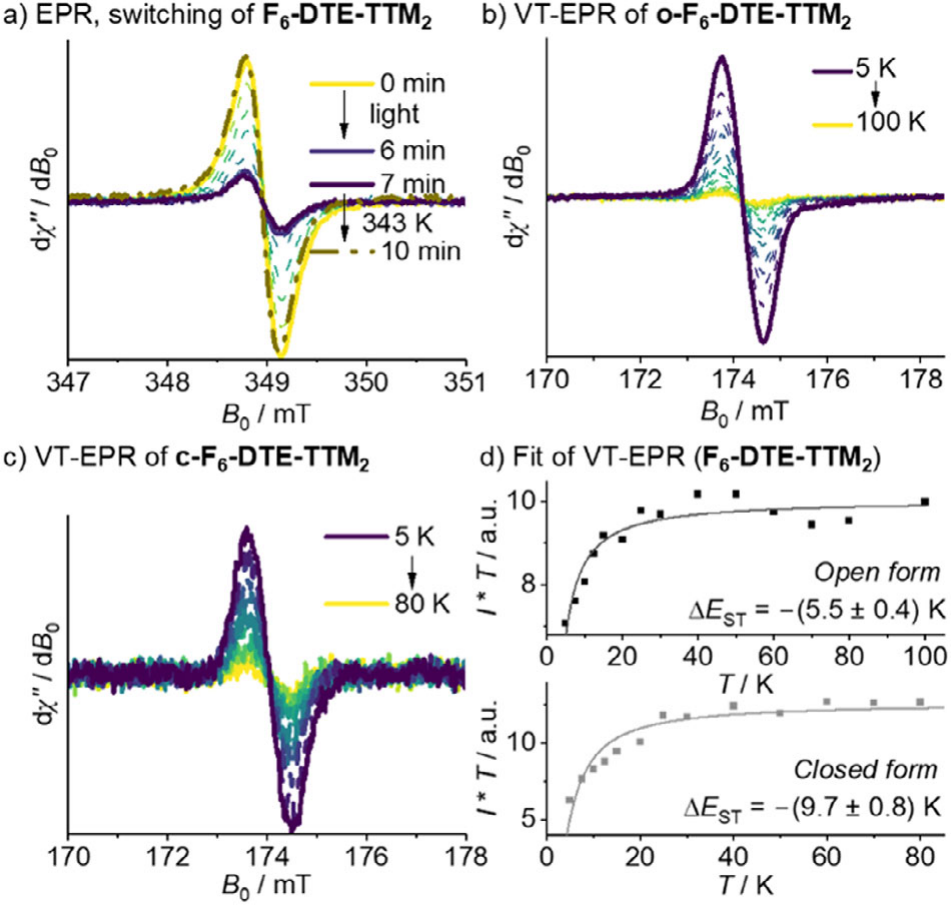
(a) Room‐temperature EPR spectra of **o‐F_6_‐DTE‐TTM_2_** upon irradiation at 430 nm and subsequent thermal recovery at 343 K. (b and c) VT‐EPR in the half‐field transition region of **o‐F_6_‐DTE‐TTM_2_** and **c‐F_6_‐DTE‐TTM2** recorded from 5 K to 100 K and 5 K to 80 K, respectively. (d) Fitting of the VT‐EPR data for **o‐** and **c‐F_6_‐DTE‐TTM_2_** using the Bleaney–Bowers model.

The magnetic properties of the switchable diradicaloids were investigated by variable‐temperature EPR (VT‐EPR) spectroscopy in frozen toluene solutions (Figures 4b,c, S30, S31, S33, and S34). Upon increasing the temperature from 5 K to 100 K for the open forms, or from 5 K to 80 K for the closed forms, the EPR signals of both switches gradually decreased in intensity while retaining their overall spectral shapes. Weaker shoulders can be observed next to the main peak of **F_6_‐DTE‐TTM_2_** in both the o‐ and c‐ forms (Figures S33b and S34b) indicating dipolar and possibly exchange interactions between the radicals, which are slightly less pronounced in **DTE‐TTM_2_** (Figures S30b and S31b). Both switches, in both their open and closed forms, exhibit half‐field transitions at low temperatures, indicating the formation of triplet states. This observation confirms the diradical nature of both switches in both their open and closed forms. Fitting the intensity of the half‐field transition as a function of temperature in accordance with the Bleaney–Bowers equation provided the singlet–triplet energy gaps, Δ*E*_ST_. For the open forms, the experimental Δ*E*_ST_ values were determined to be −(5.6 ± 0.3) K for **o‐DTE‐TTM_2_** and −(5.5 ± 0.4) K for **o‐F_6_‐DTE‐TTM_2_**, indicating nearly identical singlet–triplet gaps. The negative sign of Δ*E*_ST_ follows the −*J* exchange Hamiltonian convention and indicates antiferromagnetic coupling with a singlet ground state. After switching to the closed forms, Δ*E*_ST_ increases to −(9.2 ± 1.0) K for **c‐DTE‐TTM_2_** and −(9.7 ± 0.8) K for **c‐F_6_‐DTE‐TTM_2_**, demonstrating a small but significant modulation of the singlet–triplet energy gap (Table 1). The larger Δ*E*_ST_ values in the closed forms are consistent with enhanced conjugation and stronger antiferromagnetic exchange coupling between the two radical centers after photocyclization. These experimental values of Δ*E*_ST_, as well as the antiferromagnetic character of the exchange coupling, are in good agreement with DFT calculations performed at the UM06‐2X/def2‐TZVP‐D3Zero level of theory (Tables S2 and S3).

Since aggregation effects and irreversible degradation during open‐to‐closed switching could be excluded, and the singlet–triplet energy gap of the closed‐form diradicaloid obtained from VT‐EPR is too small to fully account for the ∼80% decrease in EPR intensity, the observed signal attenuation is likely due to a reduced diradical character in the closed form compared with the open form. This interpretation is plausible in light of the increase of π‐conjugation (optical data) and is also in qualitative agreement with the trend predicted by DFT calculations at different levels of theory (Tables S2 and S3).

## 3 Conclusion

In conclusion, we have incorporated (fluorinated) dithienylethene units into TTM‐based diradicaloids to afford two bistable diradicaloids, **DTE‐TTM_2_** and **F_6_‐DTE‐TTM_2_**, and demonstrated the rapid switching between the open and closed forms upon visible‐light irradiation and heating. As summarized in Tables 1 and S4, the diradicaloids reported herein show singlet–triplet energy gaps comparable to the pioneering diarylethene‐nitronyl nitroxide spin couplers developed by Irie and coworkers [16, 17, 18, 19, 70, 71]. However, π‐conjugation of TTM‐type radicals leads to distinct differences, in the switchable optical and electrochemical behavior as well as the modulation of EPR intensity. Overall, this work demonstrates that DTE linkers allow the external manipulation of TTM‐type diradicaloid properties and may inspire future molecular designs and applications.

## Author Contributions

**Xingmao Chang**: conceptualization, investigation, writing – original draft, writing – review and editing, methodology, data curation. **Sören Schlittenhardt**: investigation, writing – review and editing, data curation, methodology, writing – original draft. **Julia Zolg**: writing – review and editing, investigation, formal analysis. **Maximilian Herm**: investigation, writing – original draft, writing – review and editing, data curation, visualization. **Siyang He**: investigation. **Vivek Chandrakant Wakchaure**: investigation, writing – review and editing. **Dinar Abdullin**: writing – review and editing, investigation. **Robert Göstl**: writing – review and editing, supervision, funding acquisition. **Dirk M. Guldi**: funding acquisition, writing – review and editing, supervision, conceptualization. **Alexander J. C. Kuehne**: conceptualization, supervision, funding acquisition, writing – review and editing. **Sabine Richert**: conceptualization, supervision, funding acquisition, writing – review and editing. **Max von Delius**: conceptualization, supervision, funding acquisition, writing – review and editing, project administration.

## Conflicts of Interest

The authors declare no conflicts of interest.

## Supporting information



Synthesis and characterization data, SCXRD data, cyclic voltammograms, optical properties, and EPR data are included in the Supporting Information. Computational data are openly available via Zenodo under https://doi.org/10.5281/zenodo.20411821. Single‐crystal data of **F_6_‐DTE‐TTM_2_** are available in the CCDC, number 2556067. **Supporting File 1**: anie74404‐sup‐0001‐SuppMat.pdf.

## Data Availability

The data that support the findings of this study are openly available in Zenodo at https://zenodo.org/records/20411821, reference number 20411821.
