# Supplementary material for: Bistable Switching of π‐Conjugation in a Dithienylethene‐Bridged Luminescent Trityl Diradicaloid
Source: Angew Chem Int Ed Engl. 2026 Sep 4;65(43):e5705848. doi: 10.1002/anie.5705848 (PMC13676395; doi:10.1002/anie.5705848)
Supplement: Supplementary file 1 [file ANIE-65-e5705848-s001.pdf]

Supporting Information

Bistable Switching of π-Conjugation in a Dithienylethene-Bridged Luminescent Trityl Diradicaloid

Xingmao Chang, Sören Schlittenhardt, Julia Zolg, Maximilian Herm, Siyang He, Dinar Abdullin, Vivek Chandrakant Wakchaure, Robert Göstl, Dirk M. Guldi, Alexander J. C. Kuehne, Sabine Richert,* and Max von Delius*

Table of Contents

1. Materials and instrumentation 3

2. Synthesis and characterization of compounds 4

2.1 Overview of synthesis 4

2.2 Synthesis of **o-DTE-Bpin$_2$** and **o-F$_6$-DTE-Bpin$_2$** 4

2.3 Synthesis of compounds **o-DTE-TTM$_2$** and **o-F$_6$-DTE-TTM$_2$** 5

3. Recycling GPC and HPLC of radical compounds 7

3.1 Purification of open form diradicals by recycling GPC 7

3.2 HPLC of **o-DTE-TTM$_2$** and **o-F$_6$-DTE-TTM$_2$** 8

4. X-Ray crystallography data 10

5. Cyclic voltammetry measurements 12

6. Optical properties 15

6.1 Concentration-dependent UV-Vis absorption in toluene 15

6.2 Photoluminescence, quantum yield, and lifetime of diradicaloids 16

6.3 Transient absorption spectra of **o-F$_6$-DTE-TTM$_2$** and **c-F$_6$-DTE-TTM$_2$** 17

7. Switchable behaviors 19

7.1 Conversion between **o-** and **c- DTE-TTM$_2$** 19

7.2 Conversion between **o-** and **c- F$_6$-DTE-TTM$_2$** 20

7.3 Kinetics of conversion of closed- to open-form diradicaloids at room temperature 20

7.4 Calculation of the Photostationary State Distribution 21

8. EPR measurements 24

8.1 Room-Temperature EPR 24

8.2 VT- EPR of two switches in both open- and closed- forms 27

9. DFT calculations 31

10. Comparison with diarylethene-nitronyl nitroxide spin couplers 39

11. Supplementary NMR and mass spectra 41

12. References 44

1. Materials and instrumentation

All reagents and solvents were commercially available and used without further purification. Anhydrous solvents were dried prior to use in an MBraun SPS-800 instrument.

NMR data were acquired on a Bruker 400 MHz AVANCE spectrometer in CD_2Cl_2.

HRMS (MALDI-TOF) data were recorded on a Bruker Daltonic Autoflex maX using trans-2-[3-(4-tert-Butylphenyl)-2-methyl-2-propenylidene]malononitrile (DCTB) as matrix.

HPLC experiments were performed on a Shimadzu 2020 HPLC-MS equipped with a Buckyprep M column with flow rate 0.5 mL/min.

The cyclic voltammetry (CV) measurements were performed on Bio-Logic SAS (SP-150).

Recycling GPC experiments were conducted on a LaboACE LC-7080 with a column JAIGEL 2HR Plus + 2.5 HR Plus; Eluent: Dichloromethane or Toluene; Flow rate: 10 mL/min or 8 mL/min; Detector: UV-Vis 4ch LA.

UV-Vis absorption spectra were recorded on a Perkin Elmer Lambda 365 instrument or a Nanodrop OneC (Thermo Fisher Scientific). Photoluminescence measurements were carried out on a PerkinElmer FL6500 instrument. Photoluminescence quantum yields (PLQYs) were obtained from Hamamatsu Quantaurus-QY (C11347).

Room-temperature EPR measurements were performed using an X-band EPR spectrometer (CIQTEK, EPR200M). The EPR spectra of the open- and closed-form diradicals were acquired with a microwave power of 0.1 mW (30 dB), a modulation frequency of 100 kHz, a modulation amplitude of 1 mT, a conversion time of 100 ms, and a time constant of 10 ms. The microwave frequency was around 9.786 GHz. The central field was set to 349.0 mT, the sweep width to 10 mT, the field step to 0.02 mT, and the number of averages to 1.

VT-EPR spectra were measured using a Bruker Elexsys E500 X-band spectrometer equipped with a “Bruker R2” resonator and a helium flow cryostat. At the lowest investigated temperature (5 K) experimental parameters were set up to obtain a good signal-to-noise ratio, while avoiding signal saturation or broadening (see below for actual parameters). The sample was subsequently heated and once the set temperature was reached allowed to rest for 10 to allow full thermal equilibration. Measurements at the increased temperature were then performed using the same measurement parameters determined at 5 K.

The experimental microwave frequency was around 9.443 GHz and the field axes of all shown VT-EPR spectra were normalized to 9.75 GHz. Modulation frequency, conversion time and time constant were set to 100 kHz, 40.96 ms and 40.96 ms, respectively for all VT-EPR measurements. Microwave power (attenuation)/ modulation amplitude/ number of scans were chosen as follows: 0.002 mW (50 dB)/ 0.3 G/ 1 for **o-DTE-TTM$_2$** (full-field), 20.28 mW (10 dB)/ 5.0 G/ 2 for **o-DTE-TTM$_2$** (half-field), 0.02 mW (40 dB)/ 0.8 G/ 1 for **c-DTE-TTM$_2$** (full-field), 20.28 mW (10 dB)/ 10.0 G/ 12 for **c-DTE-TTM$_2$** (half-field), 0.02 mW (40 dB)/ 0.5 G/ 1 for **o-F$_6$-DTE-TTM$_2$** (full-field), 20.28 mW (10 dB)/ 10.0 G/ 4 for **o-F$_6$-DTE-TTM$_2$** (half-field), 0.02 mW (40 dB)/ 1.0 G/ 1 for **c-F$_6$-DTE-TTM$_2$** (full-field), 20.25 mW (10 dB)/ 10.0 G/ 6 for **c-F$_6$-DTE-TTM$_2$** (half-field)..

2. Synthesis and characterization of compounds

2.1 Overview of synthesis

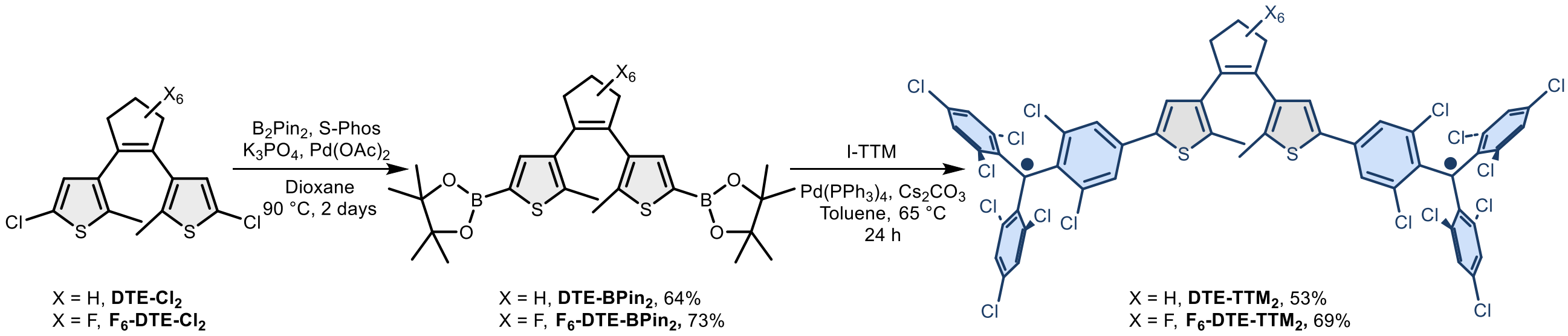

Scheme S1 Synthesis of **o-(F$_6$-)-DTE-TTM$_2$**

2.2 Synthesis of **o-DTE-Bpin$_2$** and **o-F$_6$-DTE-Bpin$_2$**

Compound **DTE-Cl$_2$** was synthesized based on the method as reported.[1]

Synthesis of **o-*DTE-Bpin$_2$***

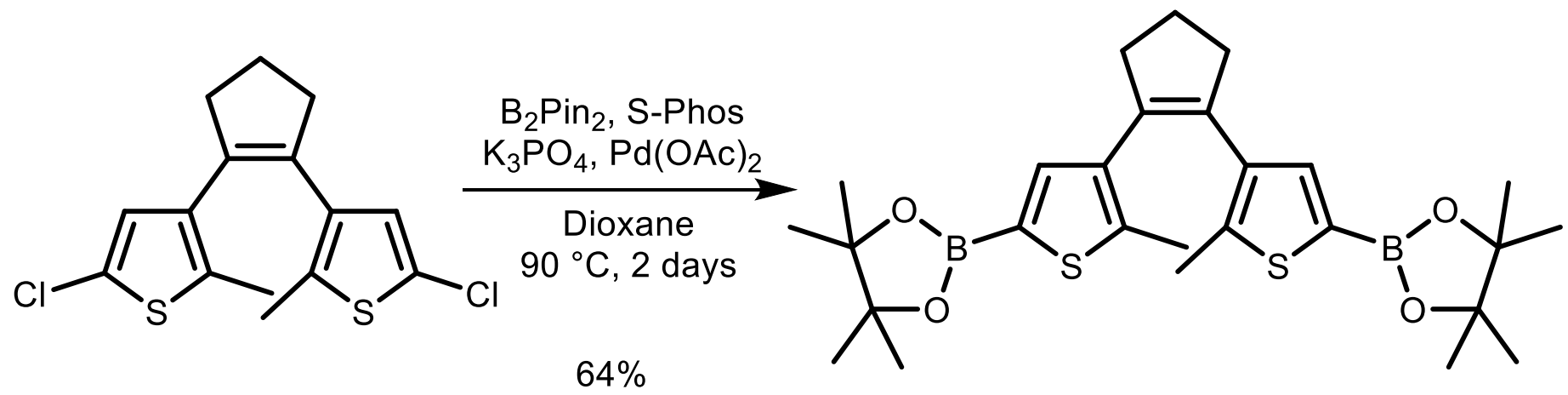

DTE-Cl$_2$ **DTE-BPin$_2$**

DTE-Cl$_2$ (150 mg, 0.46 mmol, 1 equiv.), Bis(pinacolato)diboron (1.16 g, 45 mmol, 10 equiv.), K_3PO_4 (583 mg, 2.75 mmol, 6 equiv.), S-Phos (37.5 mg, 0.09 mmol, 0.2 equiv.), and $Pd(OAc)_2$ (10.3 mg, 0.05 mmol, 0.1 equiv.) were mixed in a Schlenk flask under argon atmosphere. The anhydrous dioxane (20 mL) was added to the flask, and the mixture was stirred at 100 °C under argon for two days. After cooling to room temperature, the reaction mixture was passed through a short column of active charcoal. The solvent was removed under reduced pressure, and the residue was washed with methanol (3 mL x 3), yielding compound **o-DTE-Bpin$_2$** as a white solid (152 mg, 64%).

^{1}H NMR (400 MHz, CD_2Cl_2, 298 K): δ (ppm) = 7.29 (s, 2H), 2.78 (t, 4H), 2.03 (m, 2H), 1.87 (s, 6H), 1.30 (s, 24H).

^{13}C NMR (101 MHz, CD_2Cl_2, 298 K): δ (ppm) = 142.74, 138.79, 138.05, 134.82, 84.28, 39.24, 24.97, 23.32, 14.66.

Synthesis of **o-*F6-DTE-Bpin$_2$***

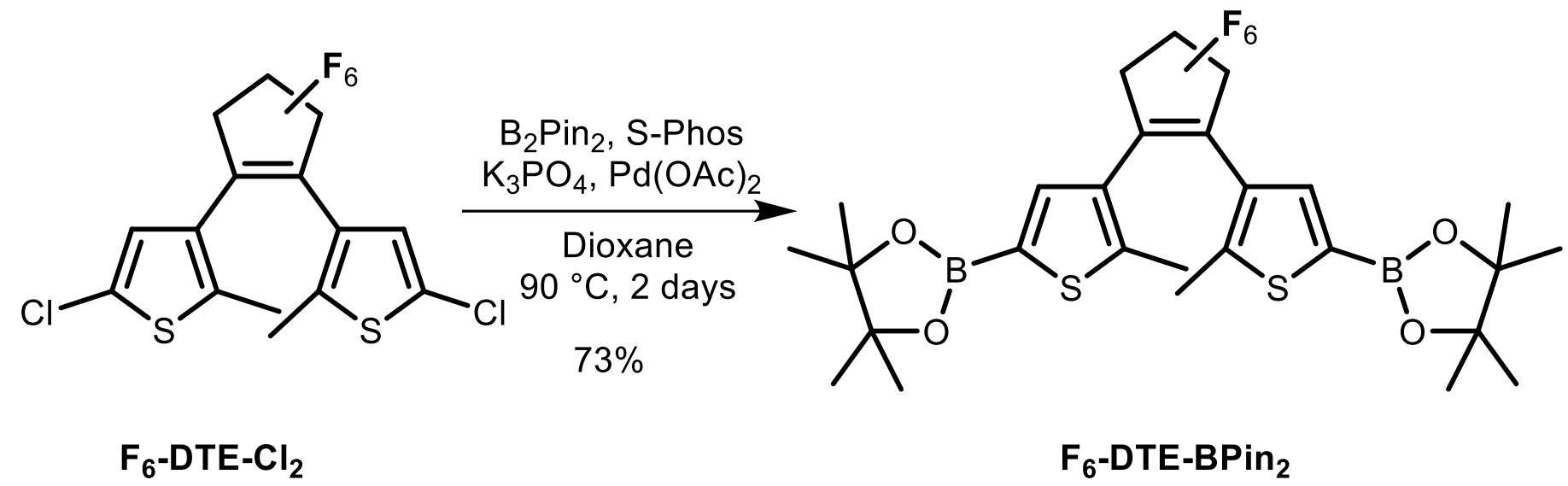

F_6-DTE-Cl_2 was synthesized according to the literature. [2]

F_6-DTE-Cl_2 (300 mg, 0.69 mmol, 1 equiv.), Bis(pinacolato)diboron (1.0 g, 4.11 mmol, 3 equiv.), K_3PO_4 (0.87 g, 4.11 mmol, 3 equiv.), S-Phos (56 mg, 0.14 mmol, 0.2 equiv.), and $Pd(OAc)_2$ (56 mg, 0.14 mmol, 0.1 equiv.) were mixed in a Schlenk flask under argon atmosphere. The anhydrous dioxane (34 mL) was added to the flask, and the mixture was stirred at 90 °C under argon for two days. After cooling to room temperature, the reaction mixture was passed through a short column of active charcoal. The solvent was removed under reduced pressure, and the residue was washed with methanol (2 mL x 3), yielding **o-F_6-DTE-$Bpin_2$** (310 mg, 73%).

^{1}H NMR (400 MHz, CD_2Cl_2, 298 K): δ (ppm) = 7.55 (s, 2H), 1.87 (s, 6H), 1.32 (s, 24H).

^{13}C NMR (101 MHz, CD_2Cl_2, 298 K): δ (ppm) = 149.41, 137.15, 126.71, 84.79, 24.96, 14.85.

^{19}F NMR (376 MHz, CD_2Cl_2, 298 K): δ (ppm) = -110.49 (t, 4F), -132.22 (p, 2F).

2.3 Synthesis of compounds **o-DTE-TTM_2** and **o-F_6-DTE-TTM_2**

TTM-I was synthesized according to our previous paper. [3]

Synthesis of compound ***o-DTE-TTM_2***

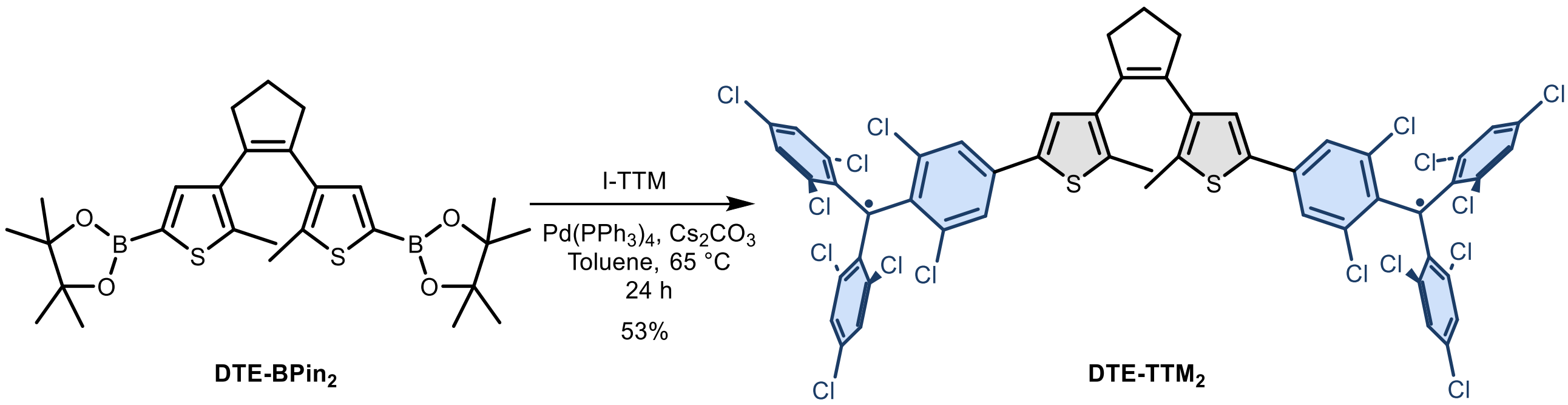

Compound **DTE-$Bpin_2$** (12 mg, 0.023 mmol, 1.0 equiv.), compound **TTM-I** (37 mg, 0.058 mmol, 2.5 equiv.), $Pd(PPh_3)_4$ (6.8 mg, 0.006 mmol, 0.25 equiv.), and Cs_2CO_3 (19 mg, 0.058 mmol, 2.5 equiv.) were mixed in a Schlenk flask under argon atmosphere. Degassed toluene (7.5 mL) with one drop of water were added to the flask, and the mixture was stirred at 70 °C under argon for two days. After cooling to the room temperature, the mixture was passed through a short aluminum oxide column. The solvent was removed under reduced pressure, and the residue were purified by silica column (Hexane:DCM,10:90) and recycling GPC (Toluene, 8 mL/min), yielding **o-DTE-TTM_2** (16 mg, 53%) as dark green solid.

Recycling GPC and **HPLC**: see section 3.

HRMS (MALDI): Calculated for $C_{53}H_{26}Cl_{16}S_2$: 1293.6374; Found 1293.6324.

Synthesis of compound ***o-F₆-DTE-TTM₂***

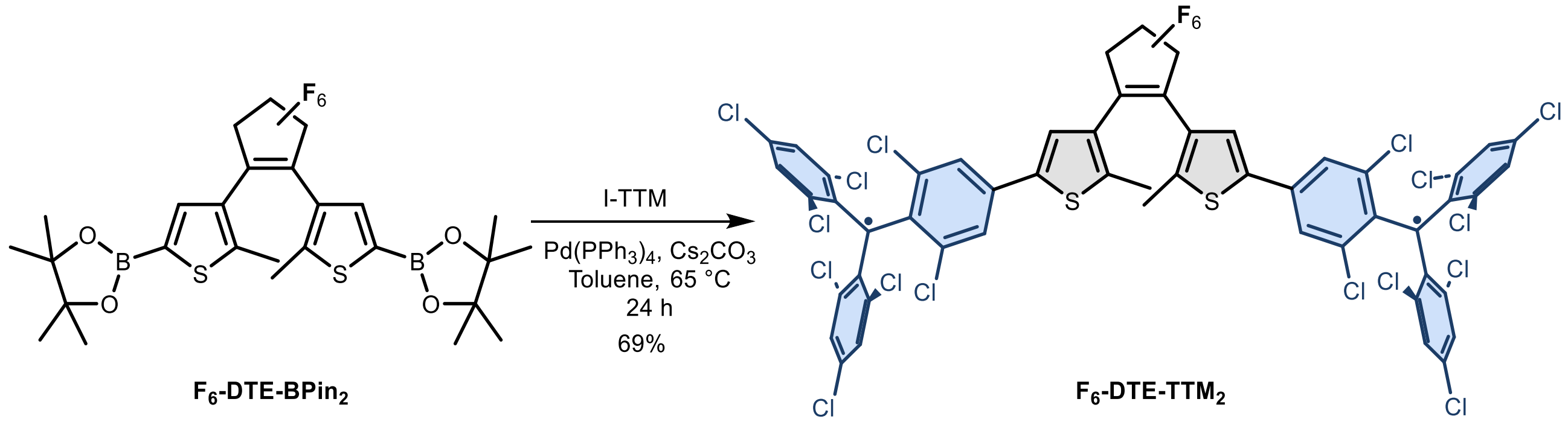

Compound **F_6-DTE-Bpin$_2$** (20 mg, 0.032 mmol, 1.0 equiv.), compound **TTM-I** (44 mg, 0.068 mmol, 2.1 equiv.), $Pd(PPh_3)_4$ (9.6 mg, 0.008 mmol, 0.25 equiv.), and Cs_2CO_3 (27 mg, 0.08 mmol, 2.5 equiv.) were mixed in a Schlenk flask under argon atmosphere. Degassed toluene (10 mL) with one drop of water were added to the flask, and the mixture was stirred at 70 °C under argon for 24 h. After cooling to the room temperature, the solvent was removed under reduced pressure, and the residue were purified by silica column (Hexane:DCM,10:90), and later by recycling GPC (DCM, 10 mL/min), yielding **o-F_6-DTE-TTM$_2$** (31 mg, 69%) as dark brown solid.

Recycling GPC and **HPLC**: see section 3.

HRMS (MALDI): Calculated for $C_{53}H_{20}Cl_{16}F_6S_2$: 1401.5809; Found 1401.5813.

3. Recycling GPC and HPLC of radical compounds

Two diradical compounds (**o-DTE-TTM$_2$** and **o-F$_6$-DTE-TTM$_2$**) are paramagnetic and therefore could not be characterized using ^{1}H NMR and ^{13}C NMR spectroscopy. Instead, they were characterized by the combination of the recycling GPC, HPLC, and HR-MS.

3.1 Purification of open form diradicals by recycling GPC

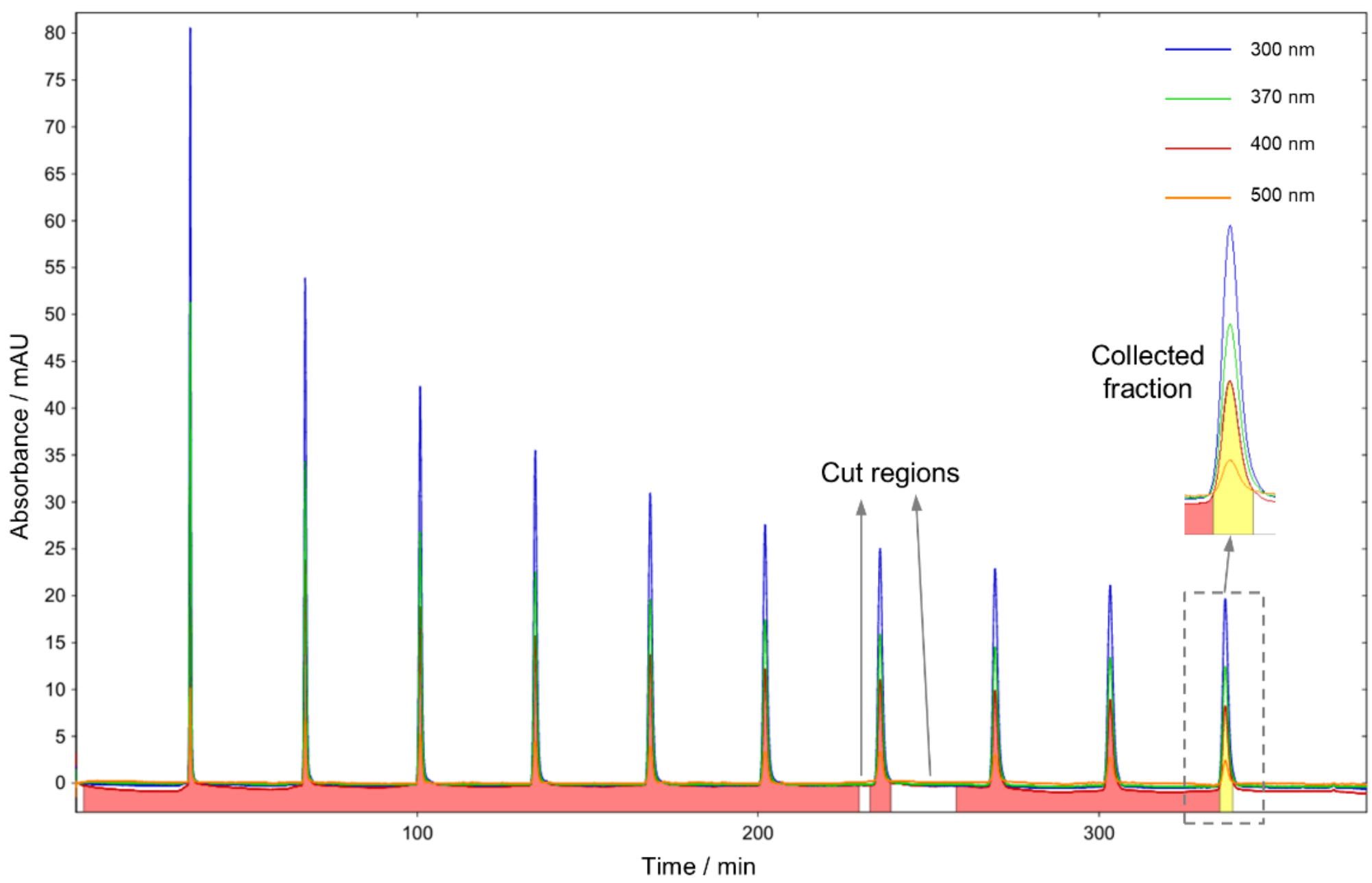

Figure S1 Recycling GPC traces of **o-DTE-TTM$_2$**. Eluent: Toluene Flow rate: 8 mL/min

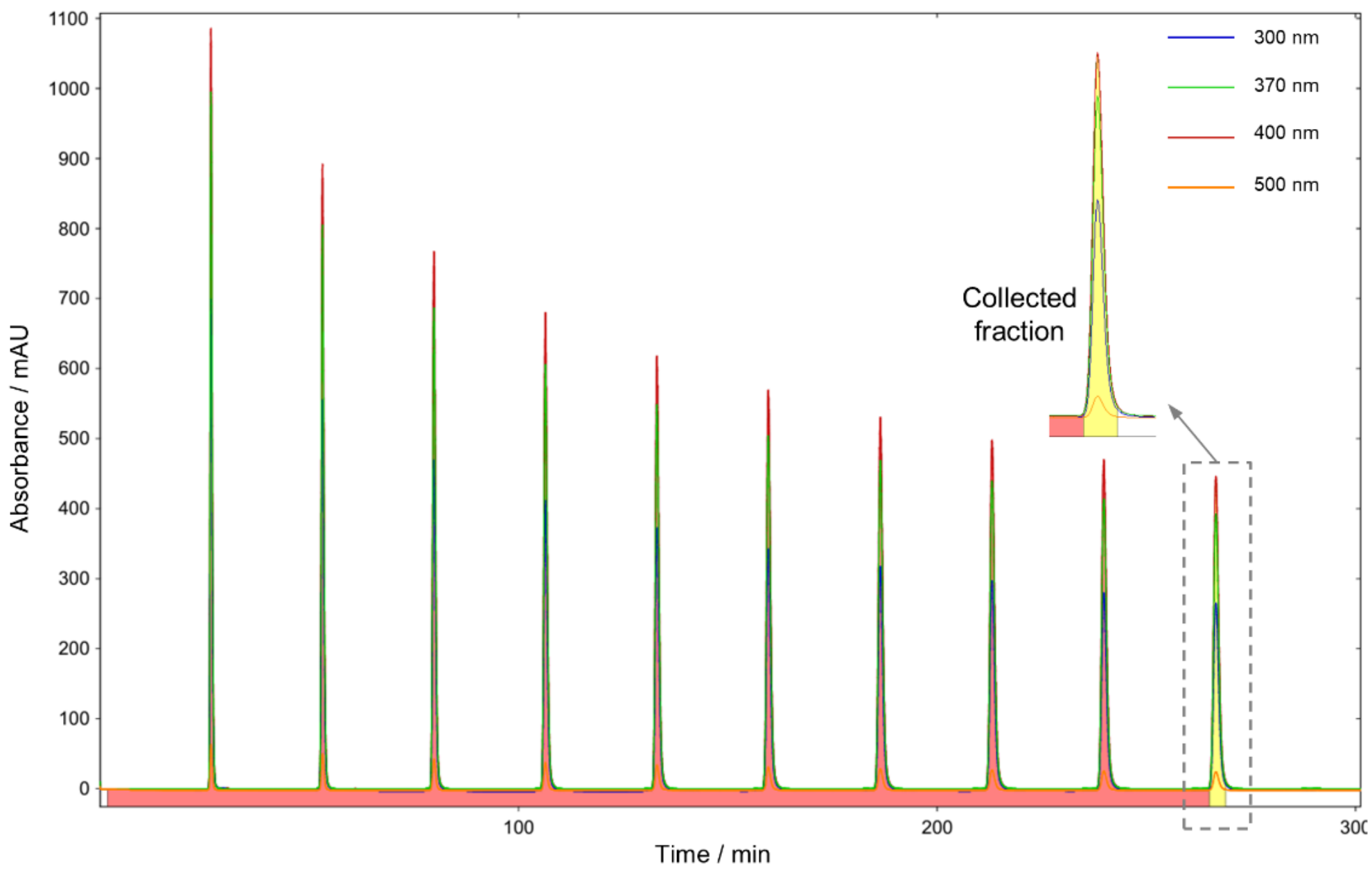

Figure S2 Recycling GPC traces of **o-F$_6$-DTE-TTM$_2$**. Eluent: Dichloromethane Flow rate: 10 mL/min

3.2 HPLC of **o-DTE-TTM$_2$** and **o-F$_6$-DTE-TTM$_2$**

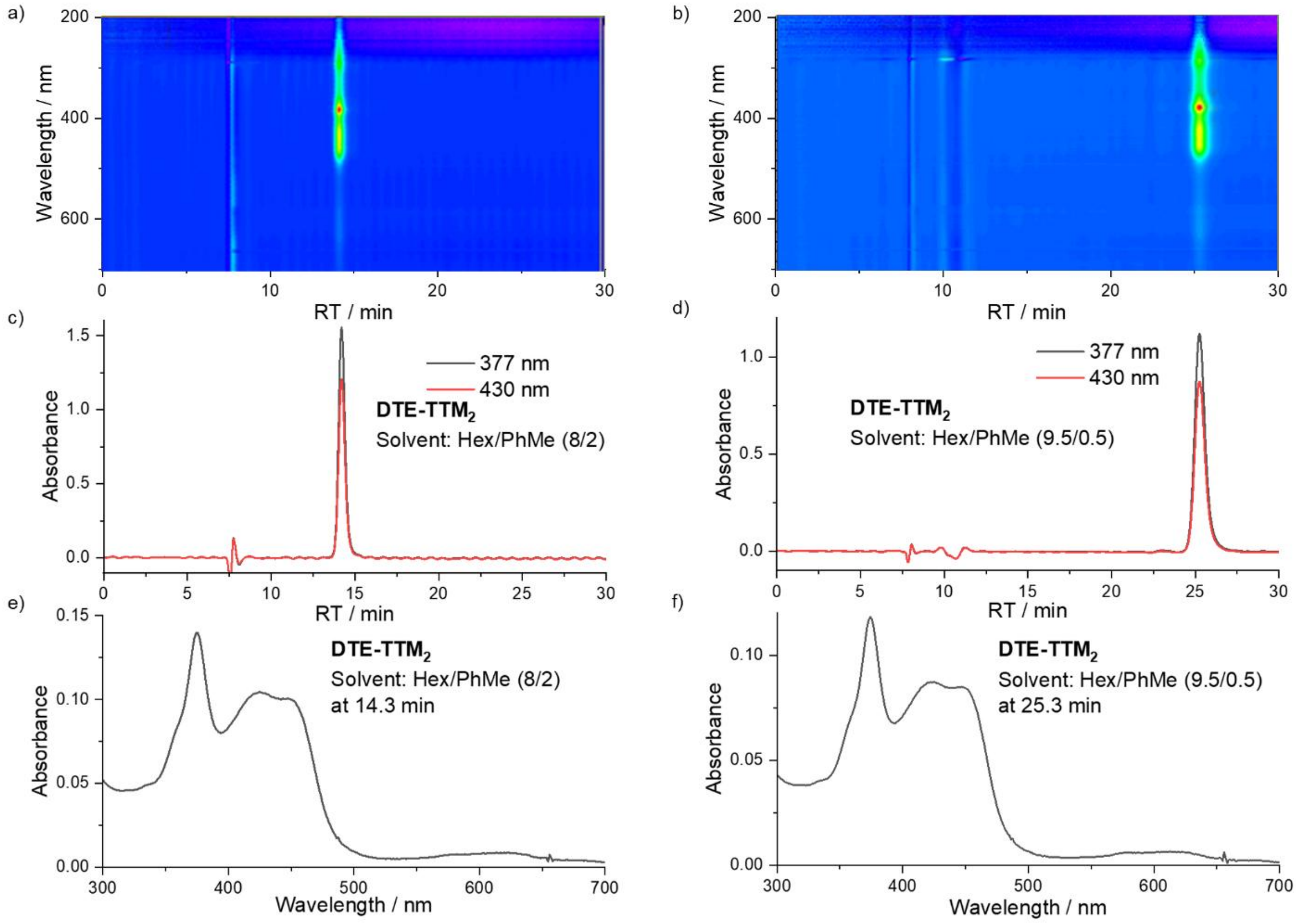

Figure S3 HPLC characterization of **o-DTE-TTM$_2$** with mixture of Hexane and as mobile phases. HPLC photodiode array mapping (a and b) and chromatogram (c and d) of **o-DTE-TTM$_2$**. The UV-Vis absorption spectra at 14.3 min (e) in mixture of hexane and toluene (8/2) and 25.3 min (f) in mixture of hexane and toluene (9.5/0.5).

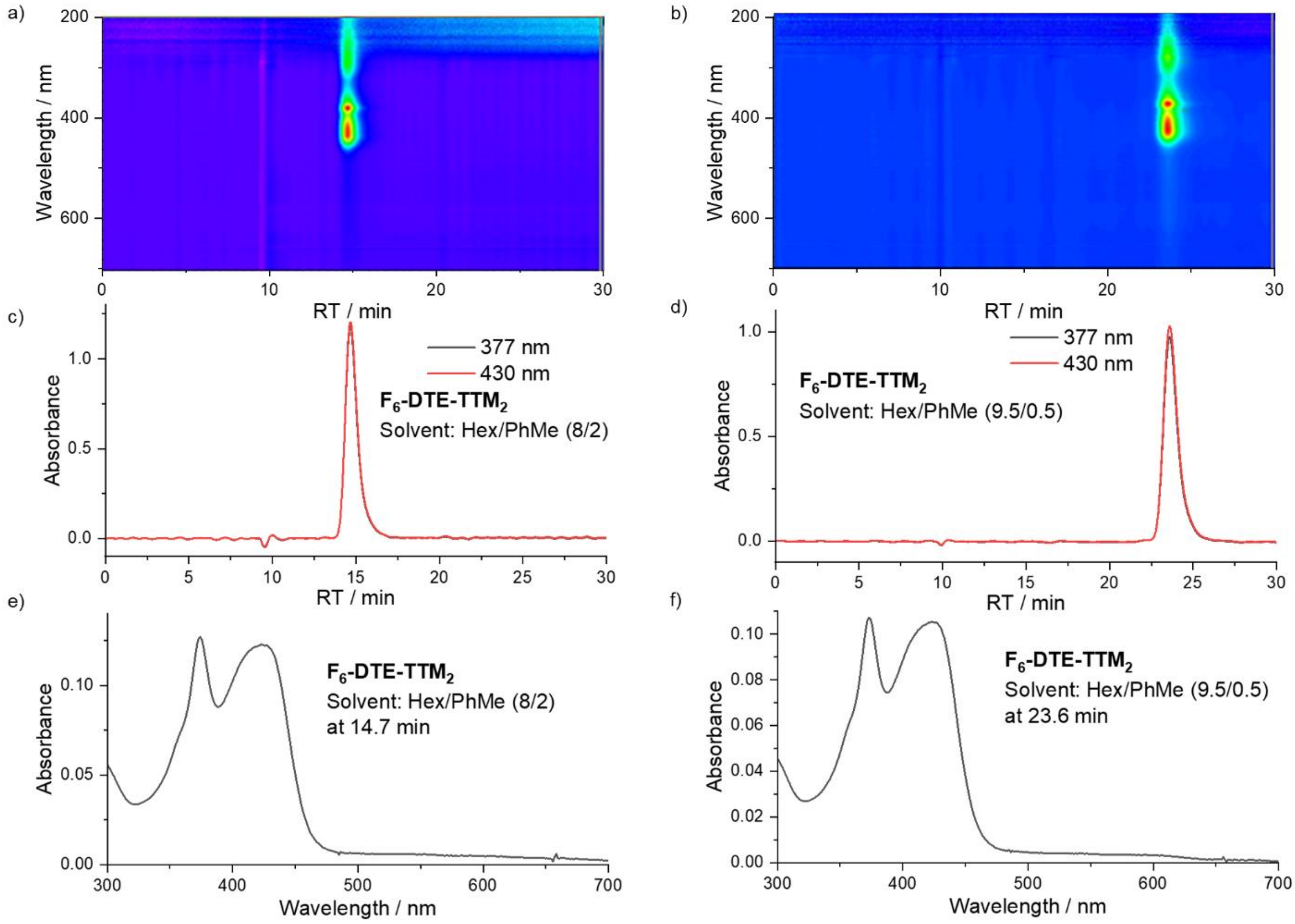

Figure S4 HPLC characterization of **o-F_6-DTE-TTM_2** with mixture of Hexane and as mobile phases. HPLC photodiode array mapping (a and b) and chromatogram (c and d) of **o-F_6-DTE-TTM_2**. The UV-Vis absorption spectra at 14.7 min (e) in mixture of hexane and toluene (8/2) and 23.6 min (f) in mixture of hexane and toluene (9.5/0.5).

4. X-Ray crystallography data

Single-crystal of **o-F_6-DTE-TTM_2** was obtained by slow diffusion of hexane/DCM.
The X ray crystallographic data was refined in Olex2 with the XT structure solution program using Intrinsic Phasing and refined with the XL refinement package using Least Squares minimization.[4-6]

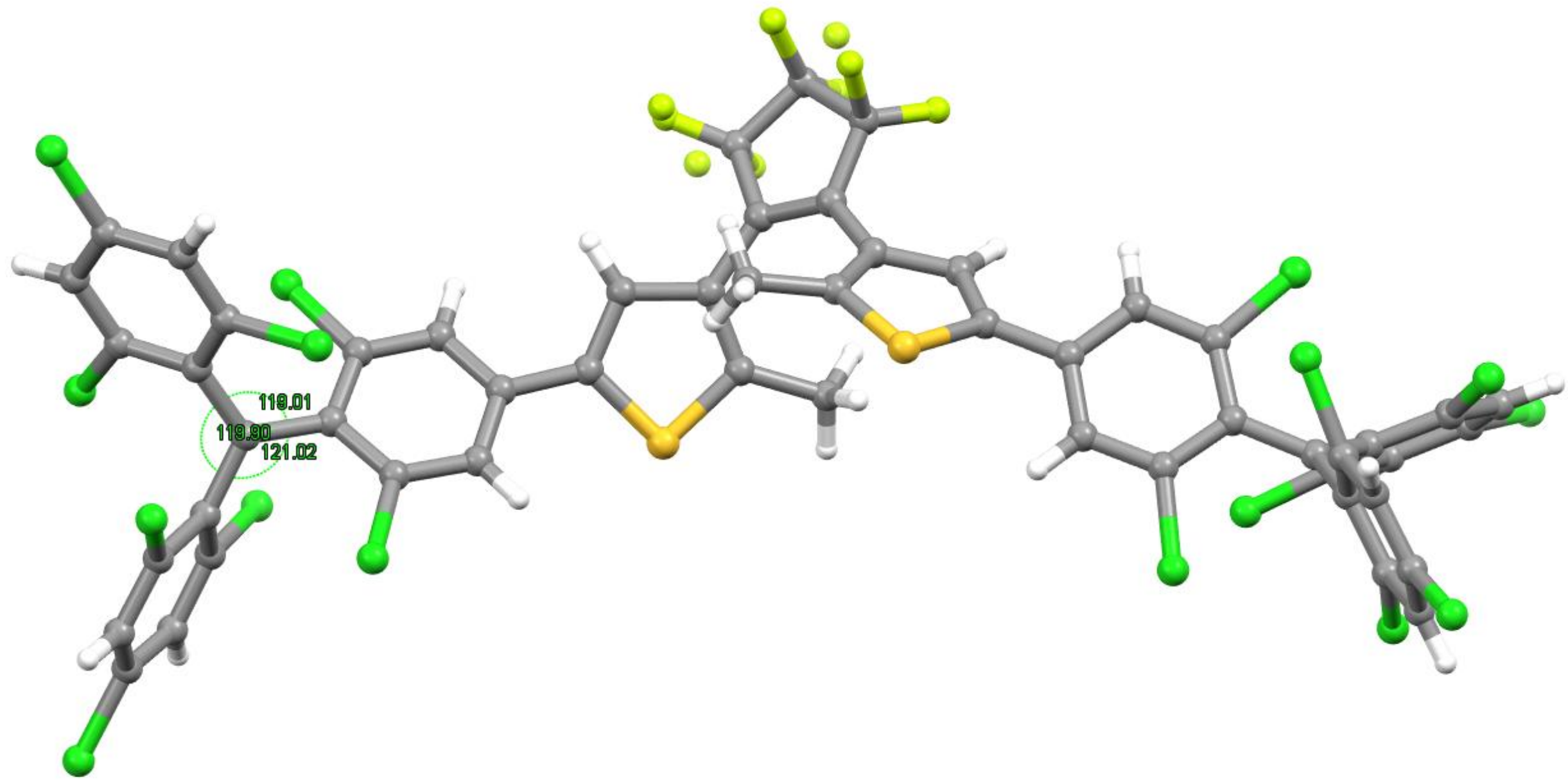

Figure S5 Single-crystal X-ray structure of **o-F_6-DTE-TTM_2** showing selected bond angles

Table S1 Crystallographic data collection and structure refinement statistics for **o-F_6-DTE-TTM_2**

CCDC numbers	**2556067**	
Bond precision	C-C = 0.0085 Å	
Wavelength	1.54184	
a / Å	43.212(3)	
b / Å	8.3039(8)	
c / Å	16.4296(10)	
α /°	90	
β / °	95.831(5)	
γ / °	90	
Temperature	100 K	
	**Calculated**	**Reported**
Volume / $Å^3$	5864.9(8)	5864.9(8)
Space group	C2/*c*	C 1 2/*c* 1

Hall group	-C 2yc	-C 2yc
Moiety formula	$C_{53}H_{20}Cl_{16}F_6S_2$ [+ solvent]	$C_{53}H_{20}Cl_{16}F_6S_2$, 1[CH2Cl2]
Sum formula	$C_{53}H_{20}Cl_{16}F_6S_2$ [+ solvent]	$C_{54}H_{22}Cl_{18}F_6S_2$
Mr / g mol^{-1}	1402.01	1486.93
Dx / g cm^{-3}	1.588	1.684
z	4	4
μ / mm^{-1}	8.017	8.874
F(000)	2784.0	2952.0
F(000’)	2815.87	
h, *k*, l_{max}	54,10,20	53,10,20
Nref	6209	5964
*T*min, *T*max	0.852,0.931	0.636,0.996
*T*min’	0.450	
Correction method	# Reported *T* Limits: *T*min=0.636 *T*max=0.996 AbsCorr = Gaussian	
Data completeness	0.961	
θmax / °	76.915	
R(reflections)	0.0764 (3416)	
*w*R2(reflections)	0.2150 (5964)	
S	1.039	
Npar	377	

5. Cyclic voltammetry measurements

A glass carbon disk and a platinum wire were used as the working electrode and counter electrode, respectively. Ag/Ag^+ was employed as the reference electrode, and redox couple Fc^+/Fc was used as the internal standard. All cyclic voltammograms were conducted in DCM (Tetrabutylammonium hexafluorophosphate, 0.1 M). The concentrations of **DTE-TTM$_2$** and **F$_6$-DTE-TTM$_2$** before and after irradiation were 1 mg/mL.

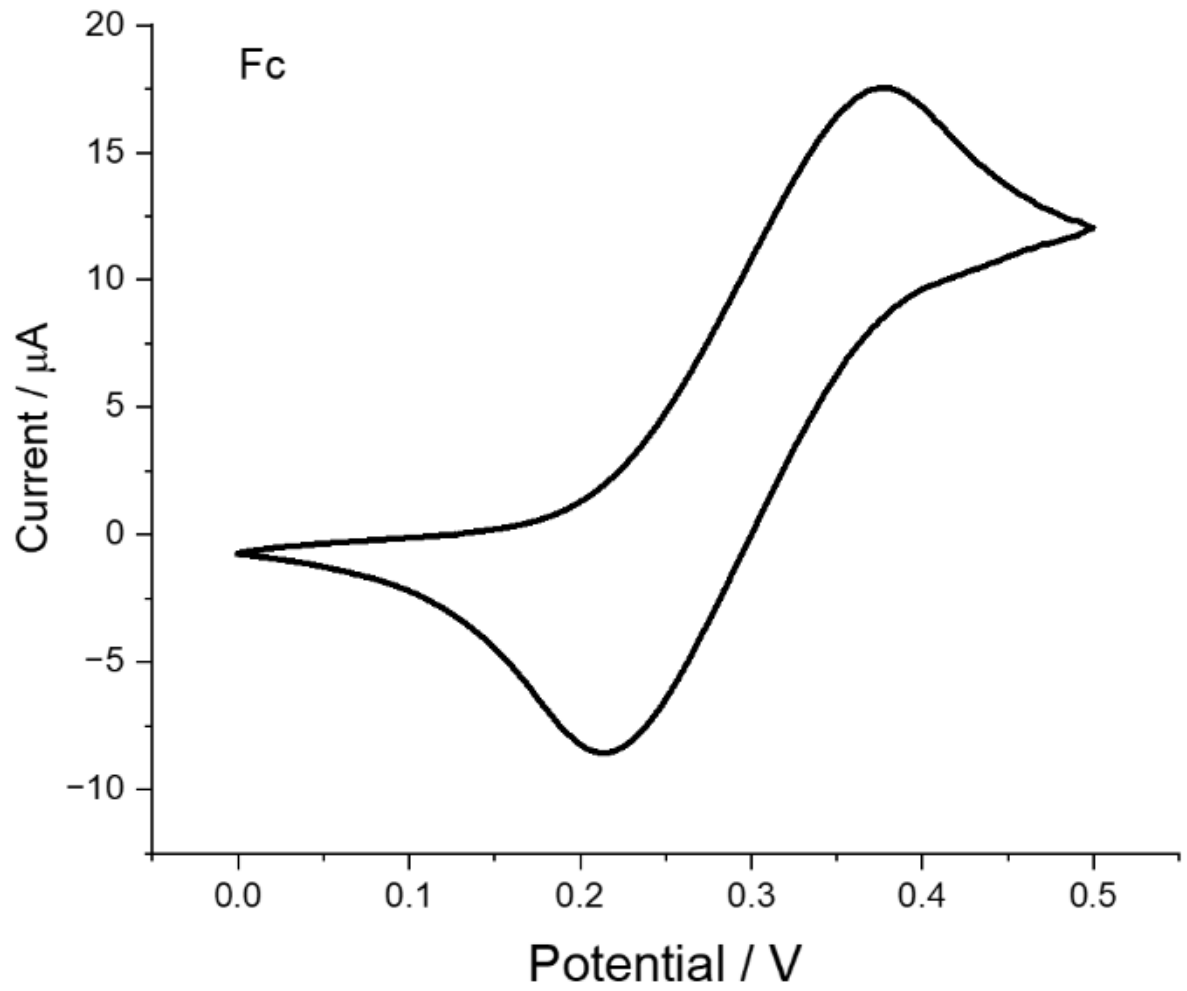

Figure S6 Cyclic voltammogram of Reference Fc at a scan rate of 100 mV/s.

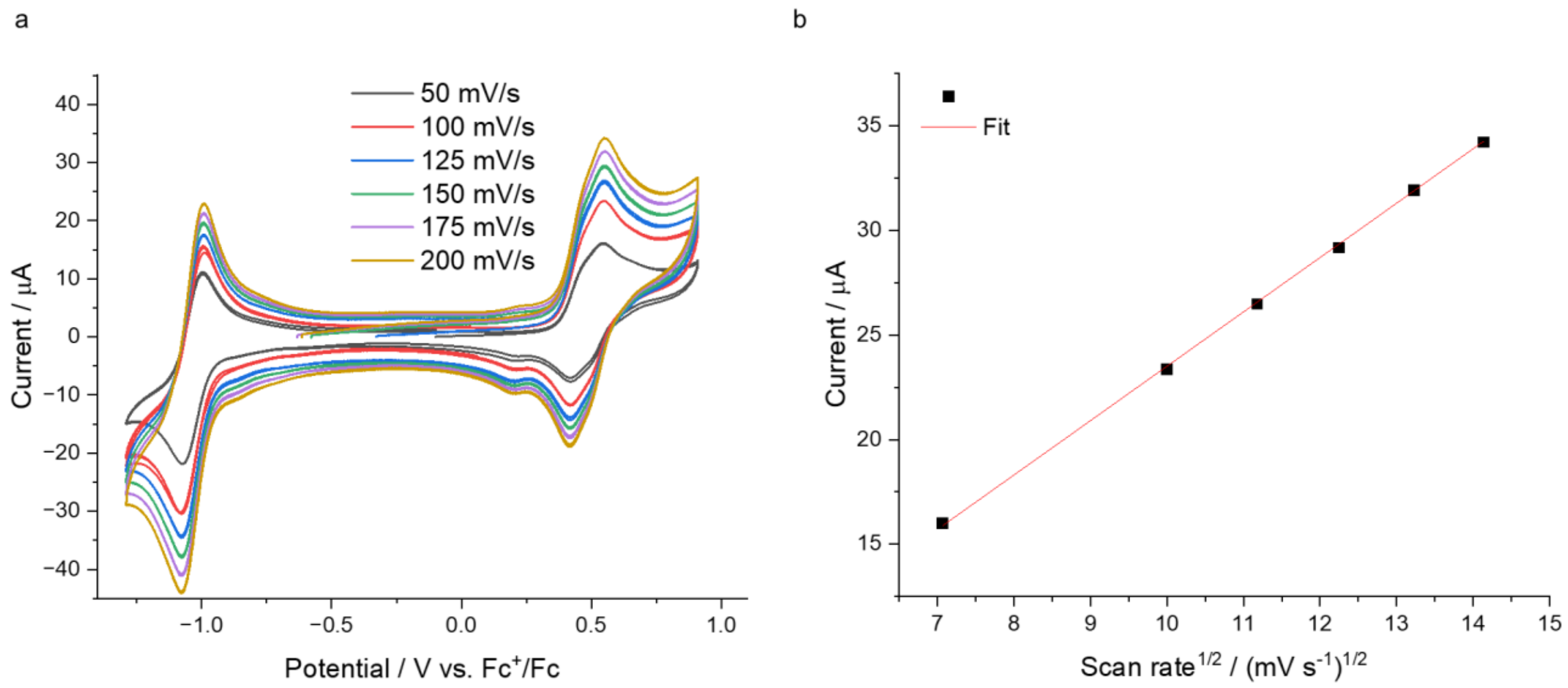

Figure S7 a) Cyclic voltammograms at different scan rates (50 - 200 mV/s) and b) linear dependence between peak anodic current (Ox) and scan rate$^{1/2}$ of **o-DTE-TTM$_2$**

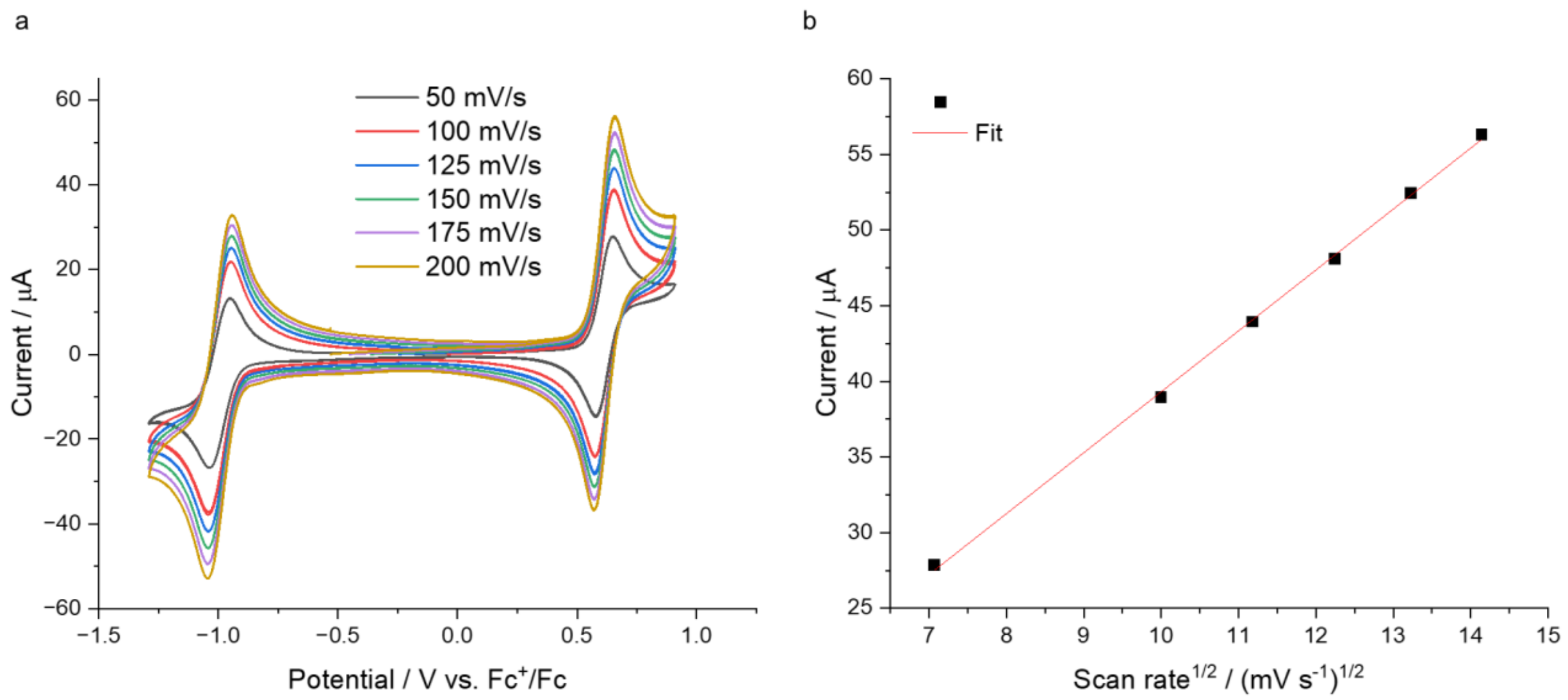

Figure S8 a) Cyclic voltammograms at different scan rates (50 - 200 mV/s) and b) linear dependence between peak anodic current (Ox) and scan rate$^{1/2}$ of **o-F_6-DTE-TTM_2**

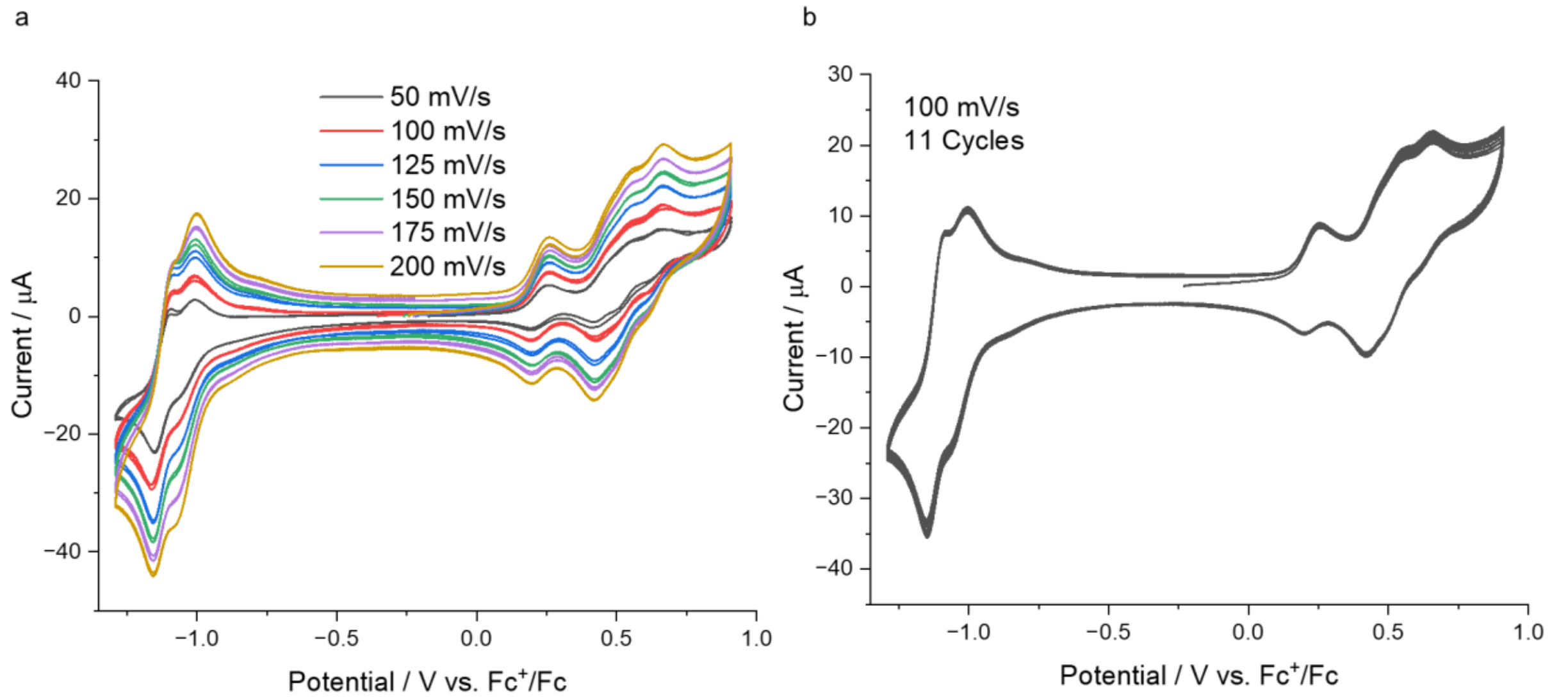

Figure S9 a) Cyclic voltammograms at different scan rates (50 - 200 mV/s) and b) 11 cycles with a rate of 100 mV/s of **Irradiated DTE-TTM_2**

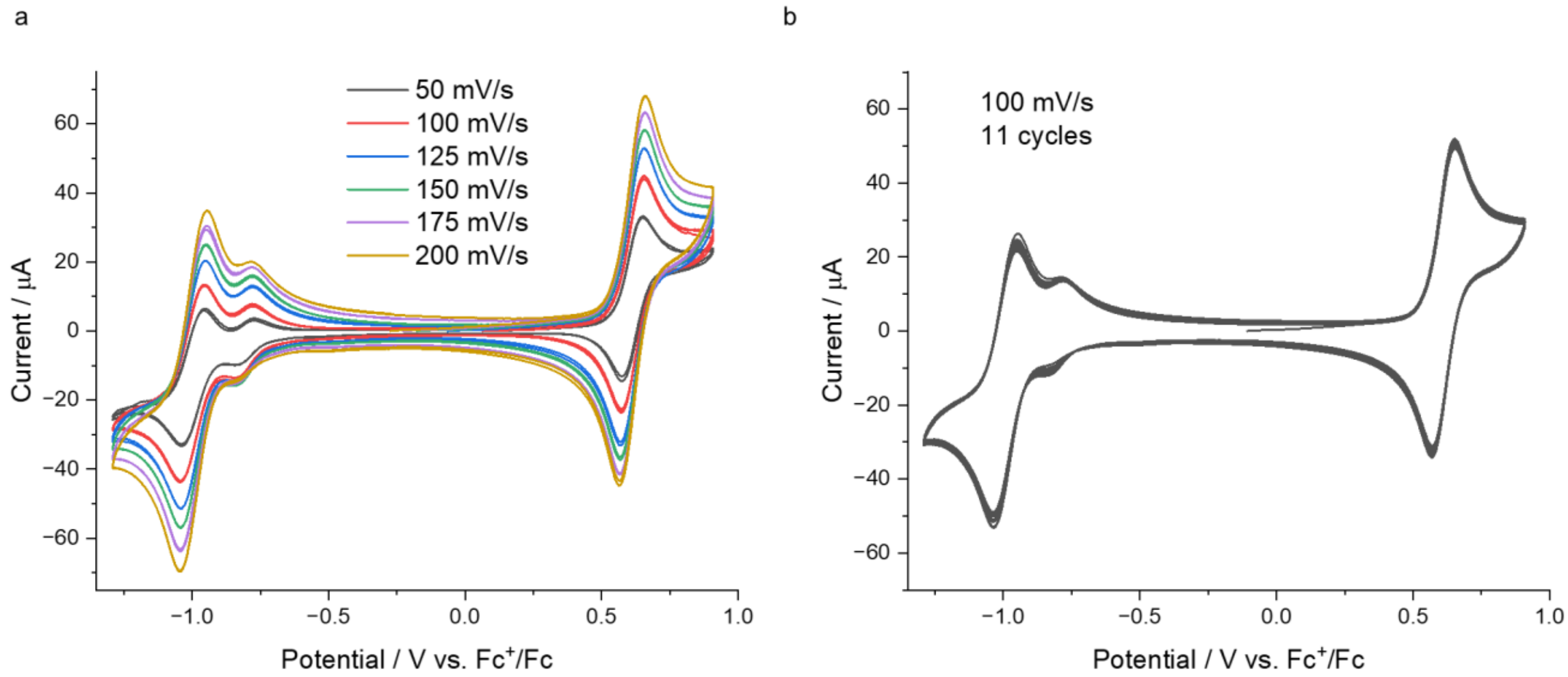

Figure S10 a) Cyclic voltammograms at different scan rates (50 - 200 mV/s) and b) 11 cycles with a rate of 100 mV/s of **Irradiated F_6-DTE-TTM$_2$**

Direct open-to-closed photoisomerization was not observed in DCM. Therefore, to obtain closed-form samples for CV analysis, the compounds were first irradiated in toluene, followed by solvent removal and redissolution in DCM. The fractions of the closed-form species in the resulting mixtures were then determined by UV–vis absorption calculated according to the Section 7.4 in SI, giving values of 69% for **c-DTE-TTM$_2$** and 87% for **c-F_6-DTE-TTM$_2$**.

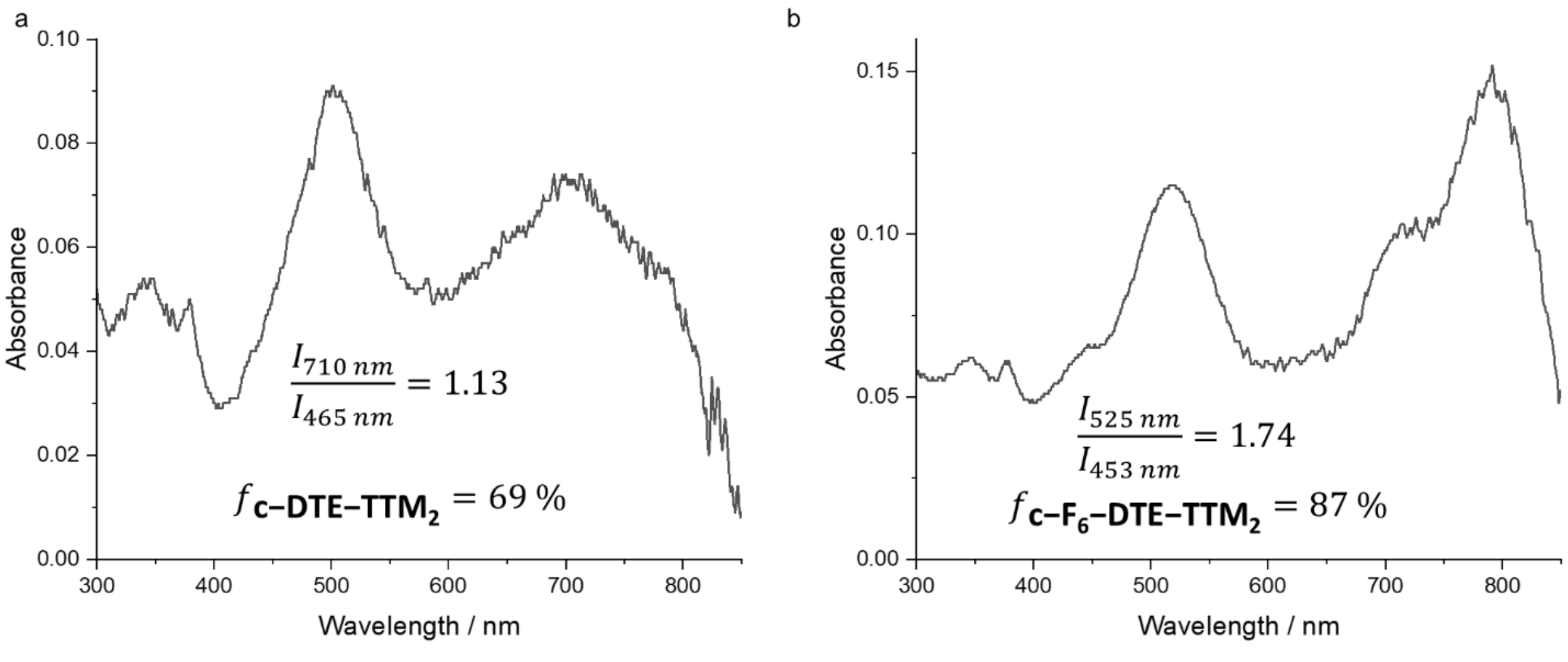

Figure S11 UV-Vis absorption spectra of irradiated samples in toluene of **DTE-TTM$_2$** (a) and **F_6-DTE-TTM$_2$** (b)

6. Optical properties

All UV-Vis absorption and photoluminescence spectra of diradicaloids (**o-DTE-TTM$_2$** and **o-F$_6$-DTE-TTM$_2$**) were measured in degassed toluene.

6.1 Concentration-dependent UV-Vis absorption in toluene

To avoid concentration effects arising from the presence of two diradicaloids during the switching process, UV–Vis absorption measurements of the two compounds (**o-DTE-TTM$_2$** and **o-F$_6$-DTE-TTM$_2$**) were performed over a concentration range from 2.5 × 10^{-7} M to 5.2 × 10^{-6} M. Within this range, the absorption bands at 377 nm and 430 nm exhibit a linear dependence on concentration.

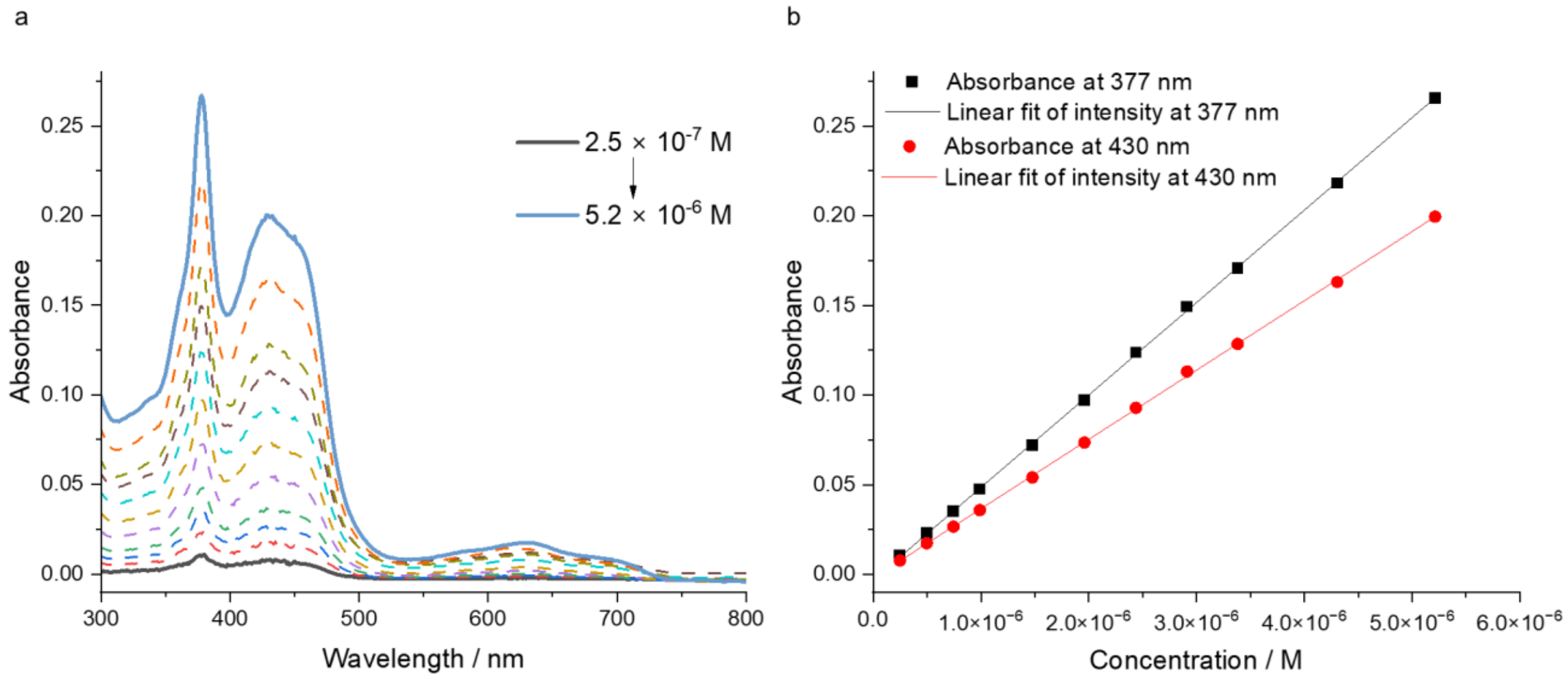

Figure S12 a) UV-Vis absorption spectra of **o-DTE-TTM$_2$** in toluene with different concentrations, b) linear fit of absorption at 377 nm and 430 nm.

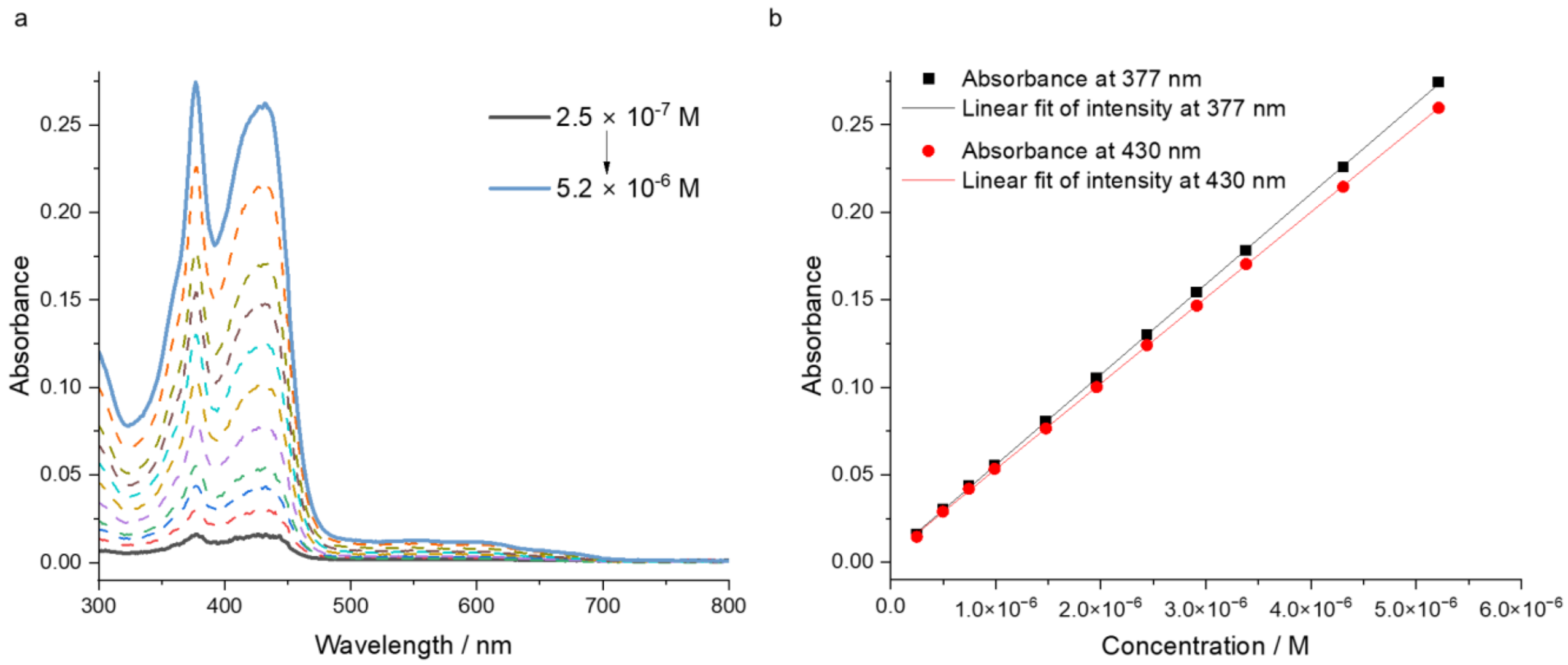

Figure S13 a) UV-Vis absorption spectra of **o-F$_6$-DTE-TTM$_2$** in toluene with different concentrations, b) linear fit of absorption at 377 nm and 430 nm.

6.2 Photoluminescence, quantum yield, and lifetime of diradicaloids

a b

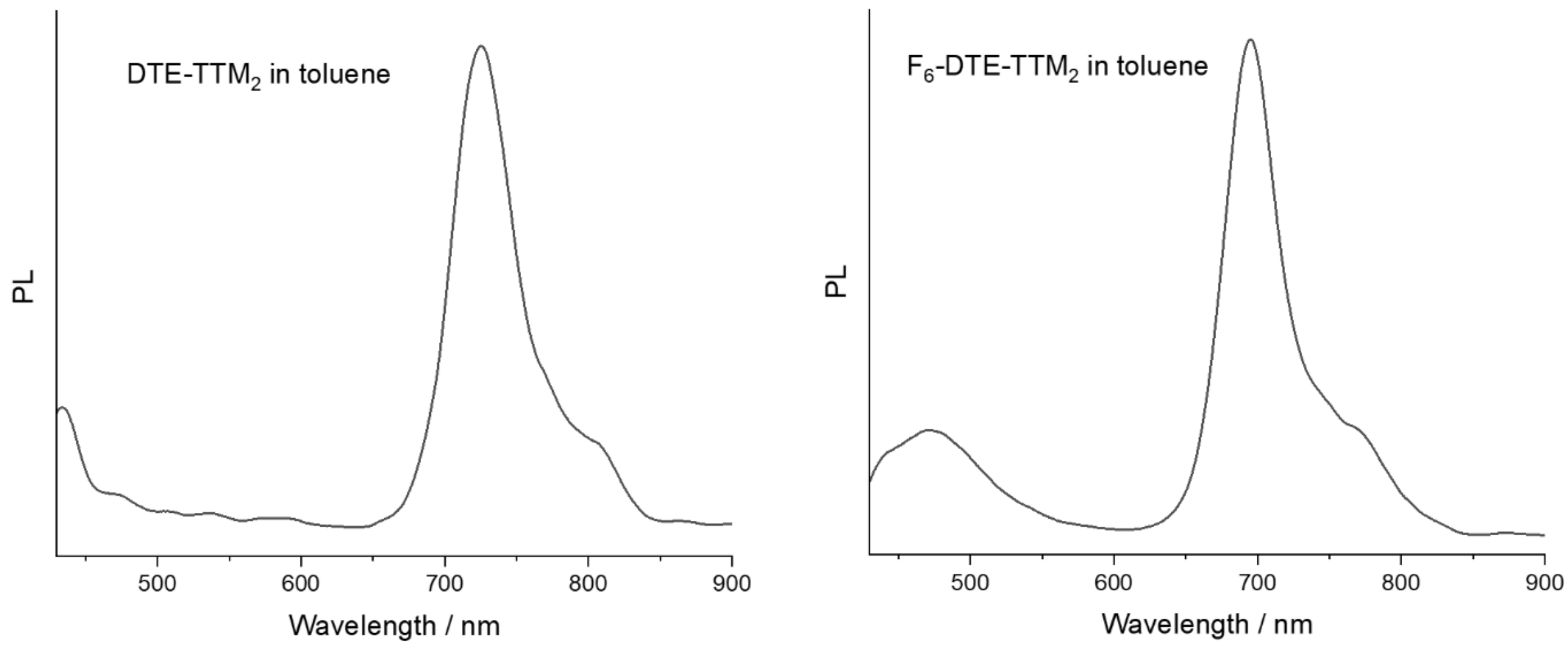

Figure S14 Photoluminescence and quantum yield of **o-DTE-TTM$_2$** and **o-F$_6$-DTE-TTM$_2$** in toluene (Concentration: 2.5 ×10^{-6} M) with excitation at 375 nm.

a b

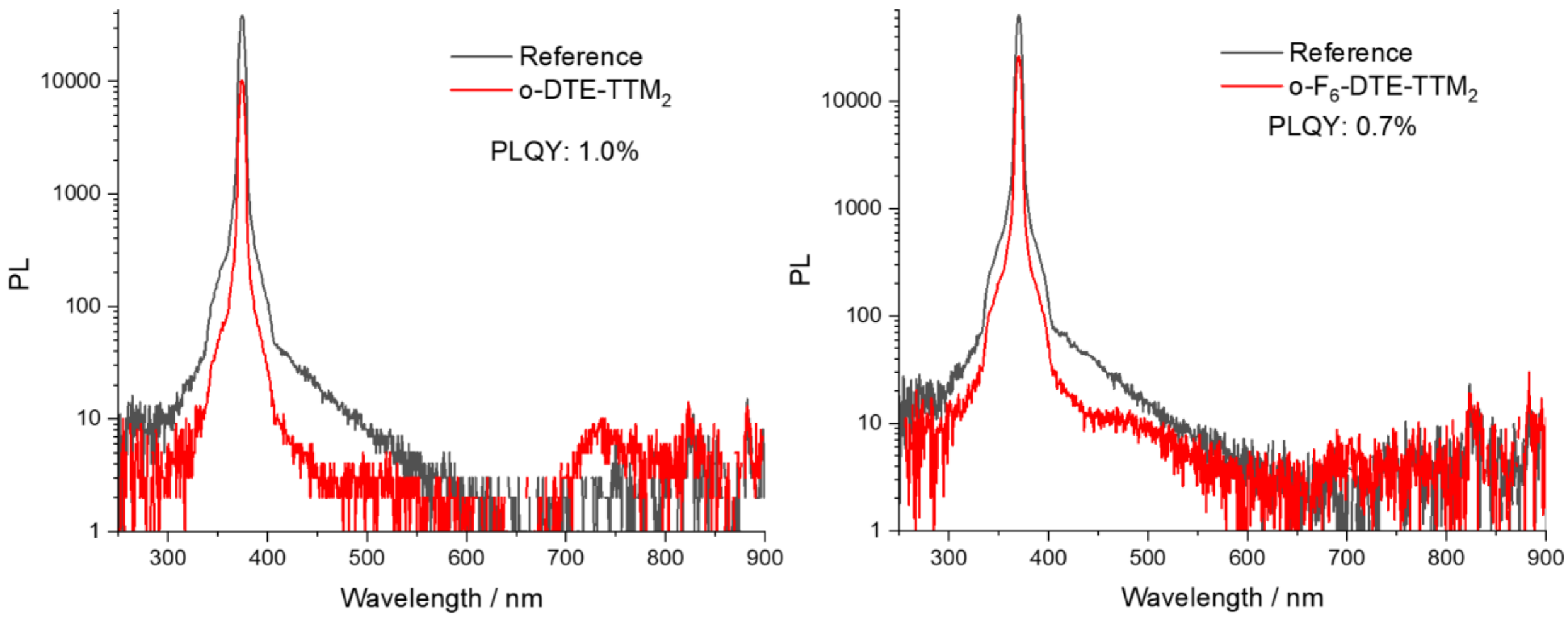

Figure S15 Photoluminescence quantum yield of **o-DTE-TTM$_2$** and **o-F$_6$-DTE-TTM$_2$** in toluene (Concentration: 2.5 ×10^{-6} M) with excitation at 375 nm.

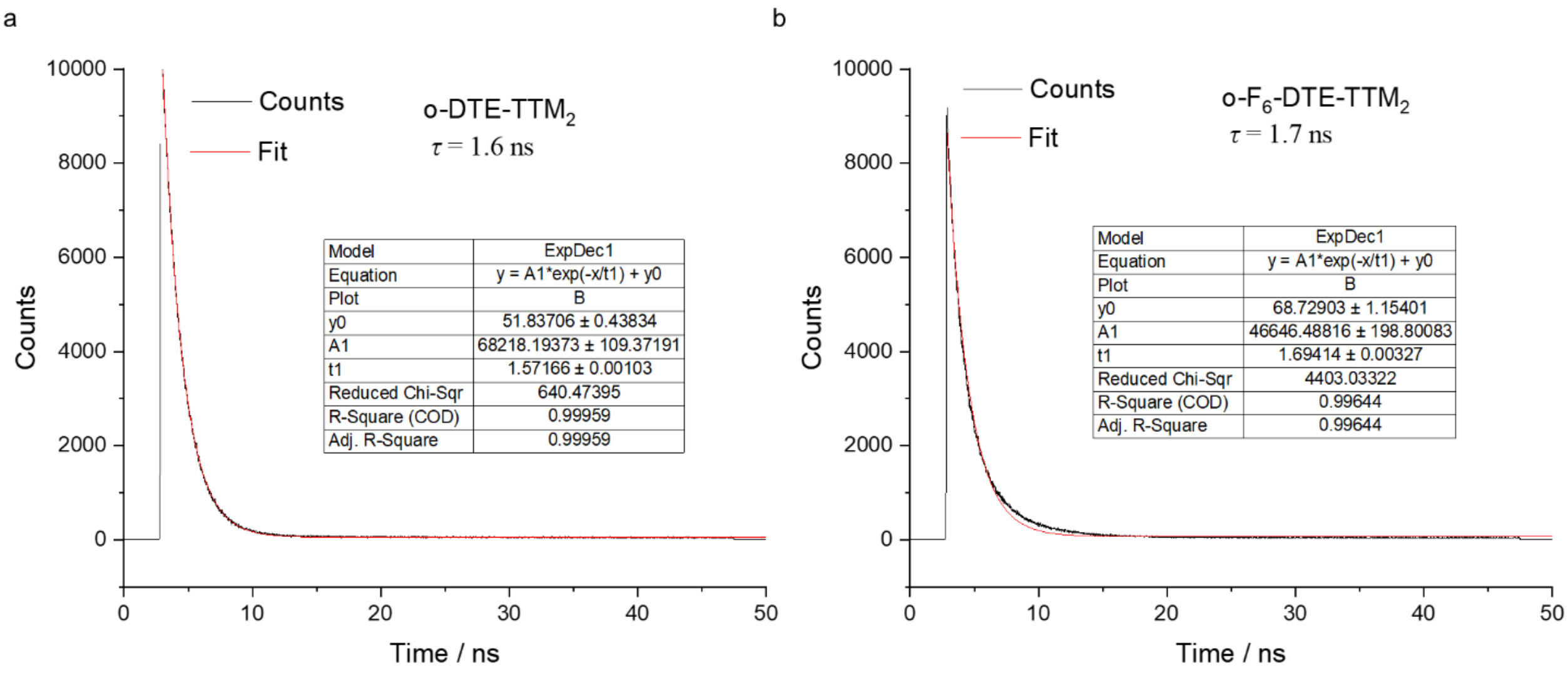

Figure S16 Photoluminescence lifetime of **o-DTE-TTM$_2$** and **o-F$_6$-DTE-TTM$_2$** in toluene (Concentration: 2.5 ×10^{-6} M) with excitation at 375 nm.

6.3 Transient absorption spectra of **o-F$_6$-DTE-TTM$_2$** and **c-F$_6$-DTE-TTM$_2$**

Ultrafast pump-probe transient absorption spectroscopy (TAS) was performed using a an Astrella-F-1K amplified Ti:sapphire femtosecond laser system from Coherent, operating at a repetition rate 1kHz, 5.5 W power (5 mJ pulse energy), pulse duration of 80 fs. To acquire the time resolved transient absorption spectra on a sub-ps resolution, an Ultrafast Systems HELIOS fs transient absorption spectrometer was used with time delays from 0 to 72 00 ps. For sub-ps, white light for the probing pulse in the visible region of the optical spectrum (~420-770 nm) was generated by focusing part of the fundamental 800 nm output onto a 2 mm sapphire disk. For (near) IR (800-1350 nm) white light, a 10 mm sapphire was used. The excitation wavelength was generated via a TOPAS Prime from Light Conversion with standard Nir/UV/Vis extension. For the fsTA measurements, global as well as global target analysis were employed for the fitting of the data, utilizing the open-source software GloTarAn (Version 1.5.1), which is a free, Java-based graphical user interface to the R-package TIMP. [7] The instrument response function (IRF) and dispersion (chirp of the white light pulse) were modelled and considered during the fitting procedure. For the measurements the energy per pump pulse was tuned to 500 nJ. All samples were prepared at room temperature in 2 x 10 mm quartz cuvettes, purged with argon for 20 min and stirred during the measurements.

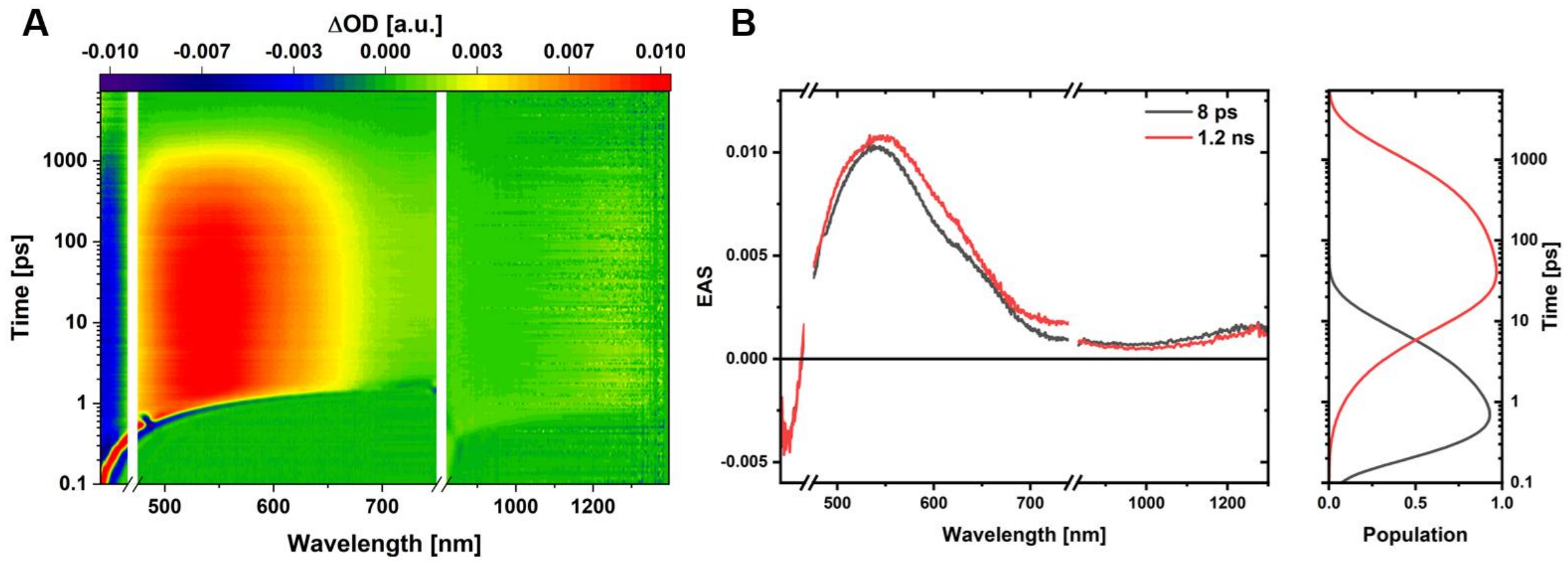

Figure S17 (**A**) Fs-differential absorption spectra of **o-F$_6$-DTE-TTM$_2$** at room temperature in argon purged toluene at time delays between 1 and 7200 ps after 420 nm photo-excitation. (**B**) Evolution-associated spectra (EAS) obtained via global analysis, applying a sequential deactivation model, and corresponding populations.

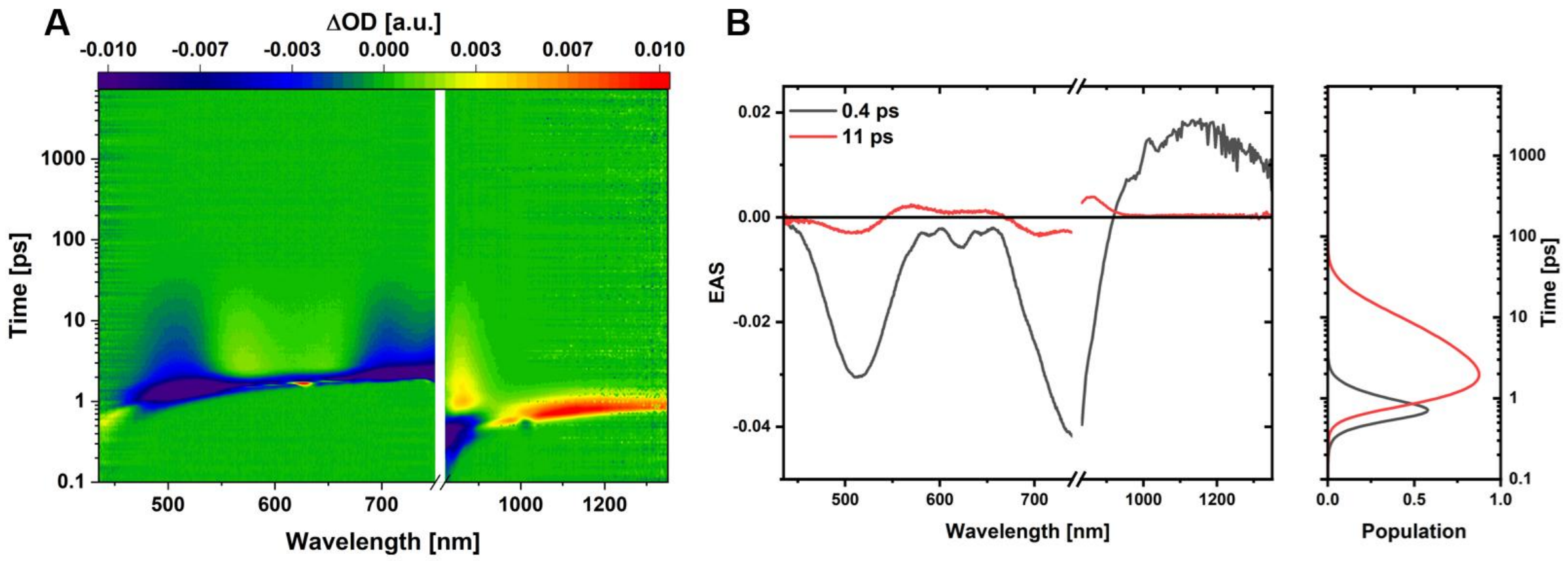

Figure S18 (**A**) Fs-differential absorption spectra of **c-F$_6$-DTE-TTM$_2$** at room temperature in argon purged toluene at time delays between 1 and 7200 ps after 780 nm photo-excitation. (**B**) Evolution-associated spectra (EAS) obtained via global analysis, applying a sequential deactivation model, and corresponding populations.

7. Switchable behaviors

The open-to-closed switching kinetics shown in Figures S15–S17 were monitored under 430 nm irradiation (lamp power: 70 mW). The closed-to-open reverse switching was first examined by irradiating the closed-form diradicaloids for 30 min with LEDs at 510 and 720 nm with different lamp power (70 mW and 1 W), which match the newly generated absorption bands of the closed forms, but the UV-Vis absorption spectra of both solutions remained essentially unchanged.For the fatigue-resistance experiments in Figure 3d and the room-temperature thermal recovery studies of the closed forms in Figures S22 and S23, the samples were first irradiated with a relatively high-power blue lamp (power: 12 W) for 5 min.

7.1 Conversion between **o-** and **c- DTE-TTM$_2$**

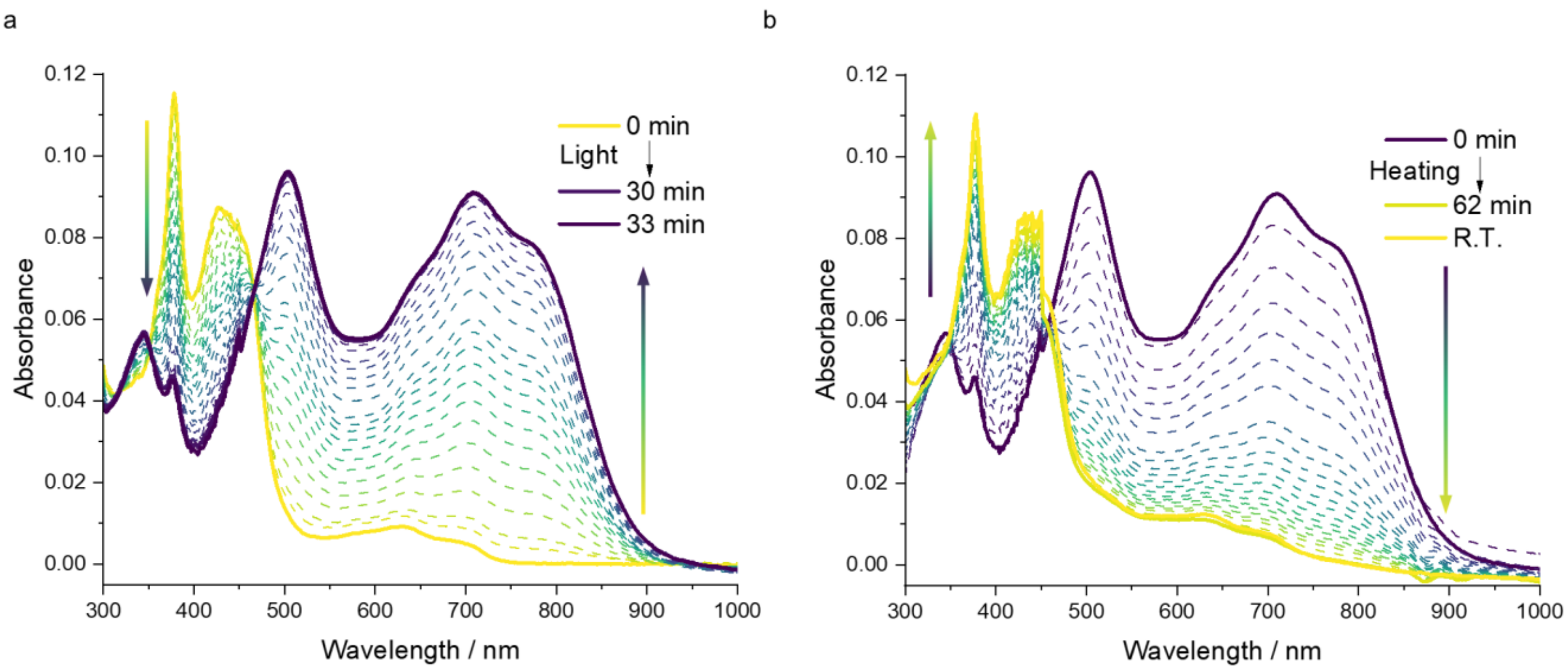

Figure S19 UV–vis absorption spectra of **o-DTE-TTM$_2$** upon irradiation at 430 nm (a) and **c-DTE-TTM$_2$** at 343 K (b), recorded at a concentration of 2.5 μM in toluene.

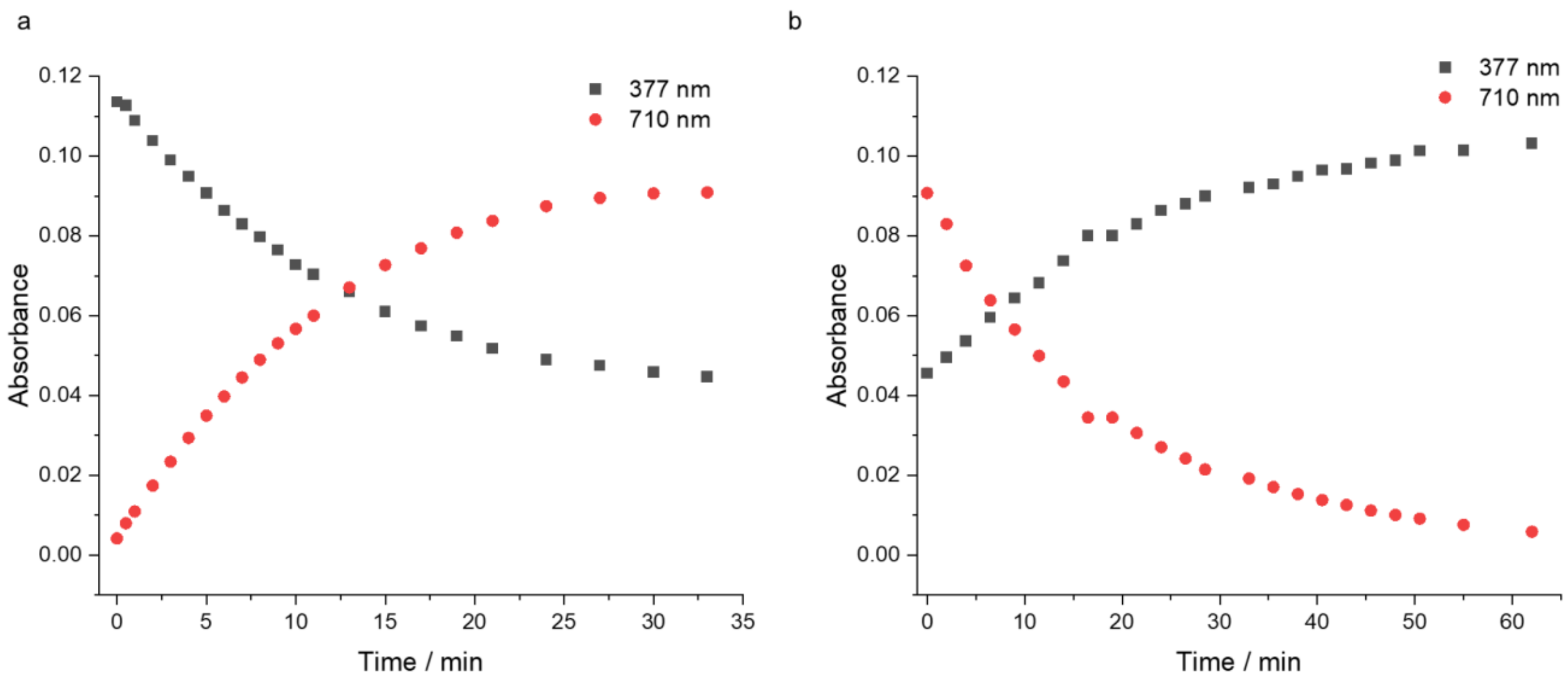

Figure S20 Kinetics of **DTE-TTM$_2$** for the open-to-closed (a) and closed-to-open (b) isomerization processes, monitored at 377 nm and 710 nm.

7.2 Conversion between **o-** and **c- F_6-DTE-TTM$_2$**

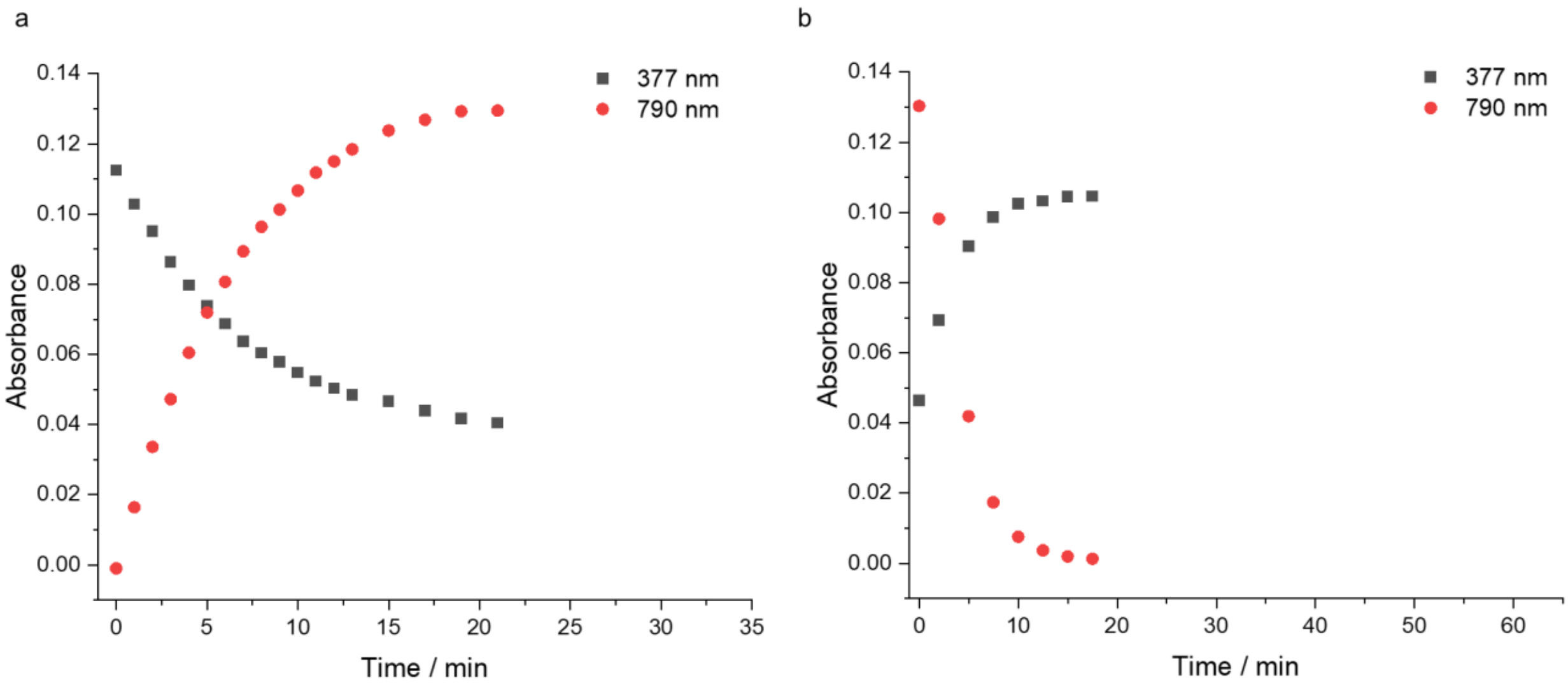

Figure S21 Kinetics of **F_6-DTE-TTM$_2$** for the open-to-closed (a) and closed-to-open (b) isomerization processes, monitored at 377 nm and 790 nm.

7.3 Kinetics of conversion of closed- to open-form diradicaloids at room temperature

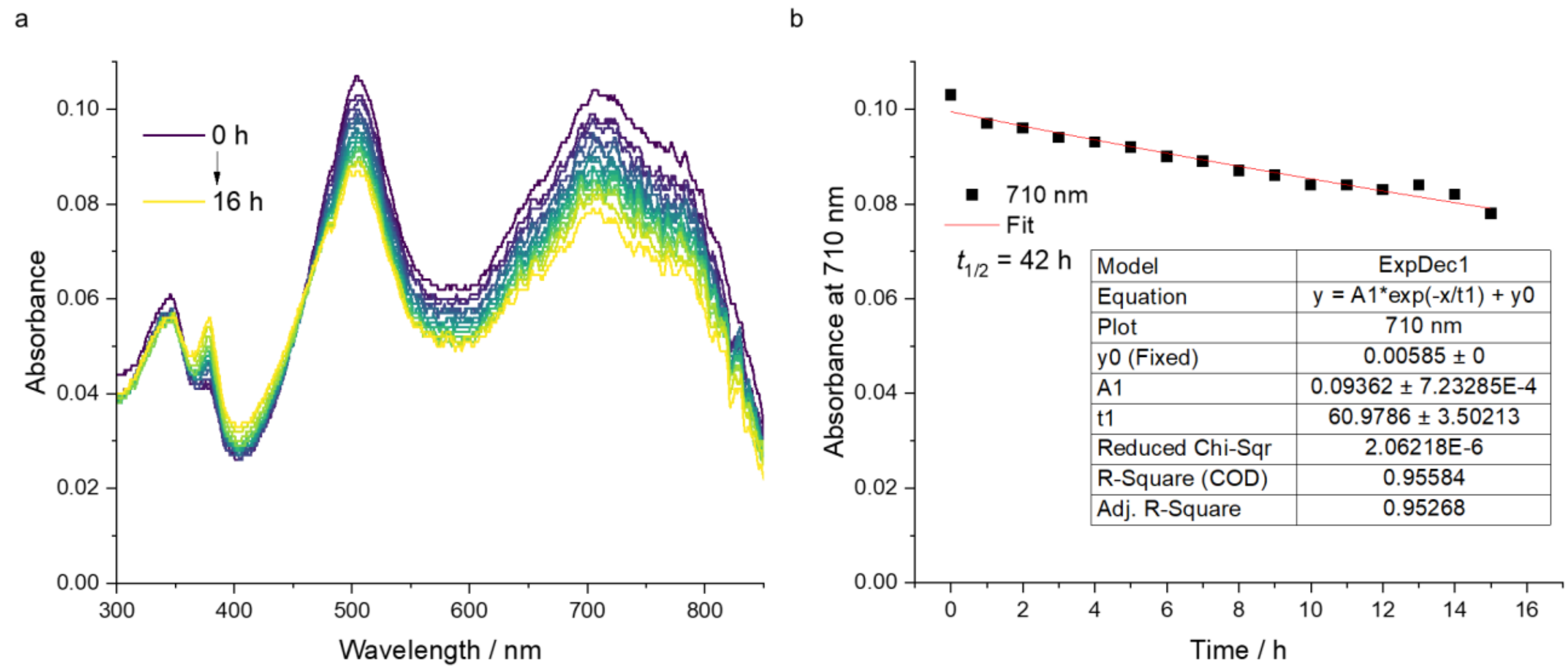

Figure S22 a) UV–vis absorption spectra of **c-DTE-TTM$_2$** at room temperature and b) decay at 710 nm, recorded at a concentration of 2.5 μM in toluene.

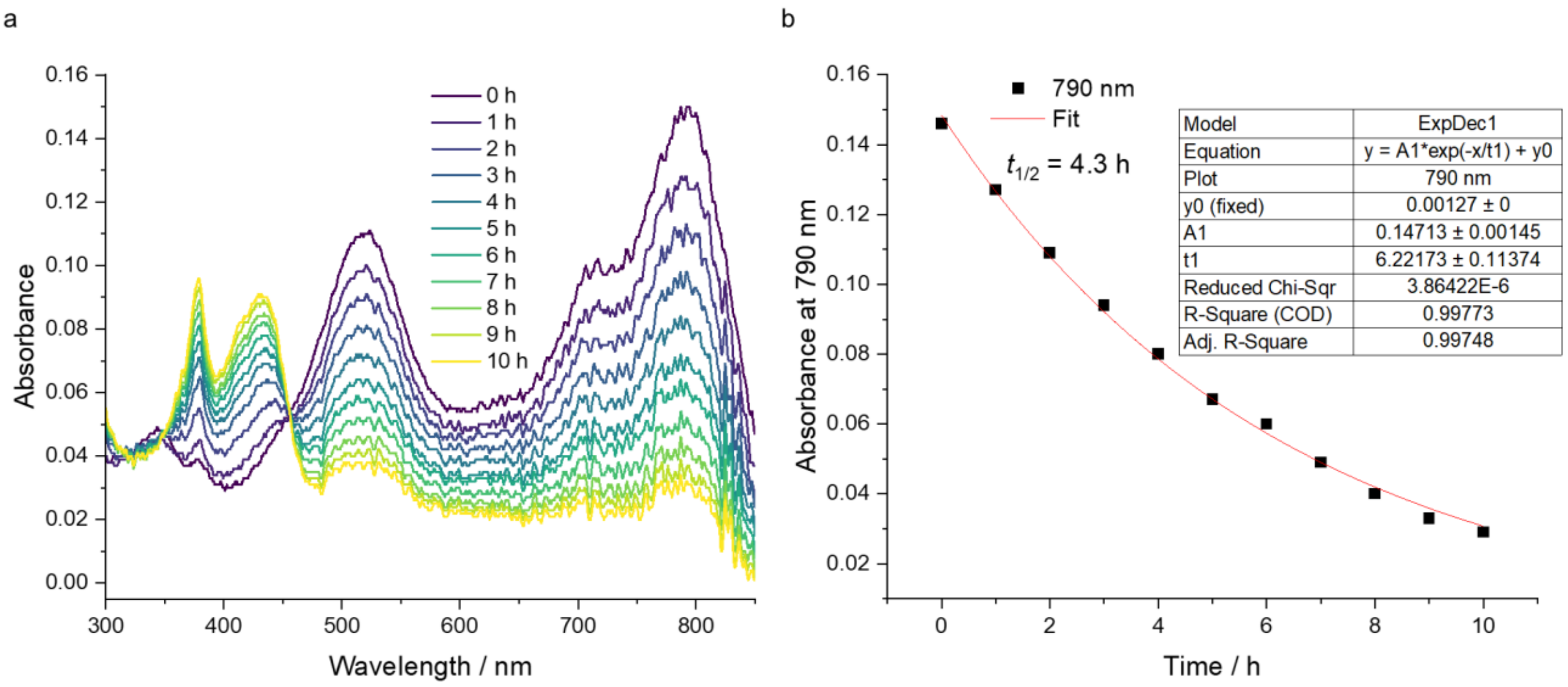

Figure S23 a) UV–vis absorption spectra of **c-F$_6$-DTE-TTM$_2$** at room temperature and b) decay at 790 nm, recorded at a concentration of 2.5 μM in toluene.

7.4 Calculation of the Photostationary State Distribution

The photostationarystate distribution (PSD) of the two diradicaloids was estimated from the absorbance ratios of the irradiated samples and compared with those of the corresponding pure closed-form species. The pure closed-form compounds were isolated by recycling GPC for **DTE-TTM$_2$** and by HPLC for **F$_6$-DTE-TTM$_2$**. For **DTE-TTM$_2$**, the absorbance ratio at 710 nm relative to that at the isosbestic point (465 nm) was used. For **F$_6$-DTE-TTM$_2$**, the absorbance ratio at 525 nm relative to that at the isosbestic point (452 nm) was used.

DTE-TTM$_2$ as an example:

$$\mathbf{A} \rightleftharpoons \mathbf{B}$$

$$PSD = \frac{[B]}{[B+A]}$$

Let f_B be the fraction of species **B** at the photostationary state (PSS), i.e.,

$$PSD_B = f_B$$

At any irradiation time, the absorbances at 465 and 710 nm can be written as

$$A_t^{710} = \varepsilon_A^{710} \times b \times c_A^t + \varepsilon_B^{710} \times b \times c_B^t$$
$$A_t^{465} = \varepsilon_A^{465} \times b \times c_A^t + \varepsilon_B^{465} \times b \times c_B^t$$

where A_t^{710} and $A_t^{465}$are the absorbances at 710 nm and 465 nm at time *t*, respectively. And ε_A^{λ} and ε_B^{λ} are the molar absorptivities of species A and B at wavelength λ.

Since 465 nm is an isosbestic point,

$$\varepsilon_A^{465} = \varepsilon_B^{465}$$

and the total concentration is conserved:

$$c^t = c_A^t + c_B^t$$

The ratio of absorptance at 710 nm to 465 nm could be written as

$$\frac{A_t^{710}}{A_t^{465}} = \frac{\varepsilon_A^{710} \times b \times c_A^t}{\varepsilon^{465} \times b \times c} + \frac{\varepsilon_B^{710} \times b \times c_B^t}{\varepsilon^{465} \times b \times c}$$

And the f_B could be written as

$$f_B = \frac{c_B^t}{c}$$

Thus, the ratio could be written as

$$\frac{A_t^{710}}{A_t^{465}} = \frac{\varepsilon_A^{710}}{\varepsilon^{465}} \times (1 - f_B) + \frac{\varepsilon_A^{710}}{\varepsilon^{465}} \times f_B$$

Defining the absorptance ratio as

$$R = \frac{A^{710}}{A^{465}}$$

The ratio at any time could be

$$R_t = R_A \times (1 - f_B) + R_B \times f_B$$

Then the f_B at PSS sate could be written as

$$f_B = \frac{R_{PSS} - R_A}{R_B - R_A}$$

In the system of DTE-TTM$_2$:

The values of R_A and R_B were obtained from the separated open-form and closed-form species by recycling GPC, using the integrated detector responses at 710 and 465 nm (Figure S24). The following values were obtained:

$$R_A = 0.05$$

$$R_B = 1.62$$

And the ratio at PSS could be obtained from the UV-Vis absorption spectrum

$$R_{PSS} = 1.37$$

Thus, the PSD of **DTE-TTM$_2$** for the open-to-closed form is **84%**.

In the system of F$_6$-DTE-TTM$_2$:

The same procedure was applied using 525 nm and isosbestic point at 453 nm. The values of R_A and R_B determined from the separated open-form and closed-form species by HPLC using the integrated detector responses at the two wavelengths (Figure S25). The ratio for the irradiated sample at the PSS was obtained from the UV–Vis absorption spectrum.

$$R_A = 0.20$$

$$R_B = 1.97$$

$$R_{PSS} = 1.92$$

Thus, the PSD of **F_6-DTE-TTM$_2$** for the open-to-closed photoisomerization is **97%**.

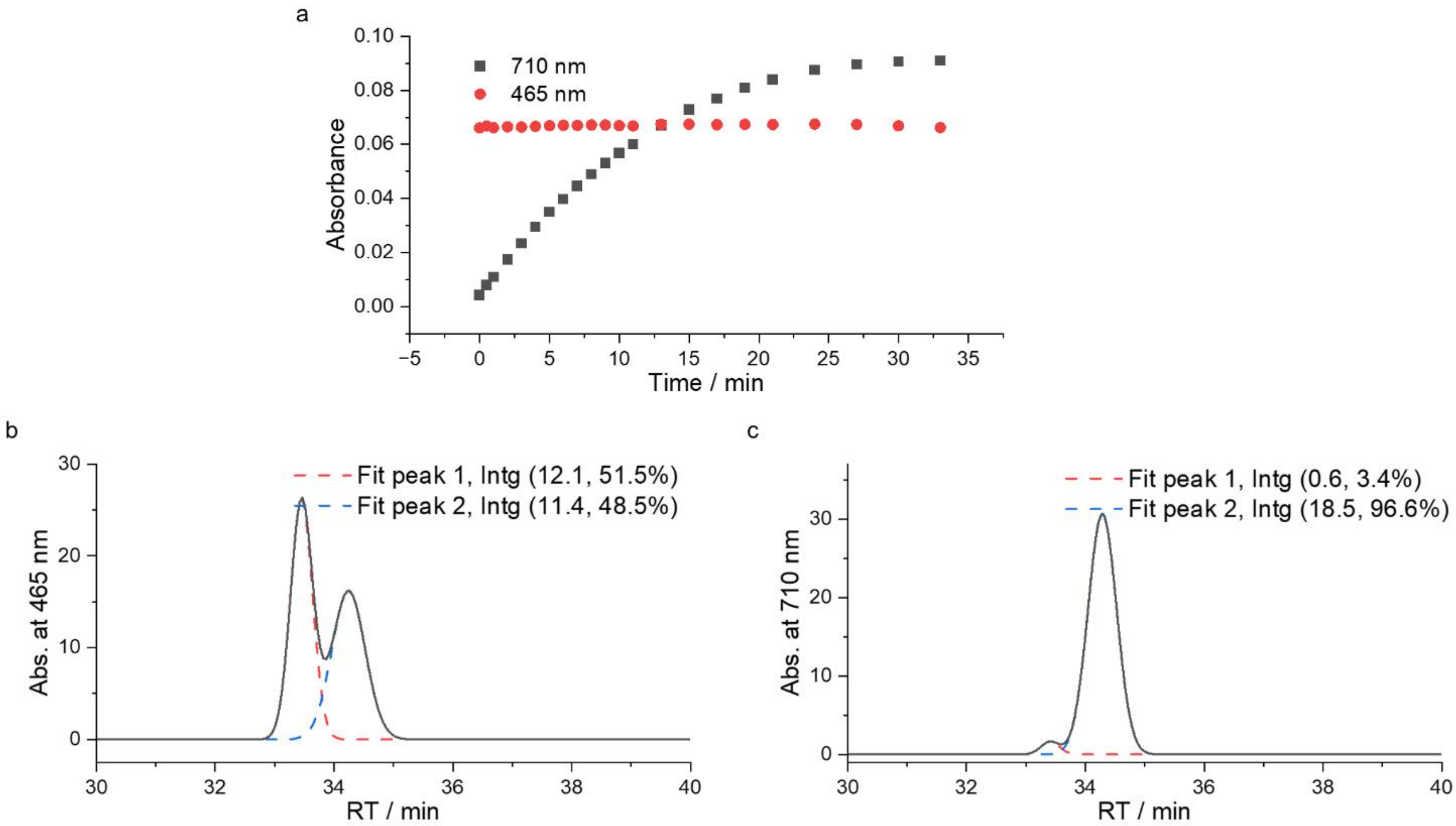

Figure S24 a) Time-dependent absorbance changes with light irradiation of **DTE-TTM$_2$** monitored at 710 and 465 nm. b and c) Extracted recycling GPC traces of **DTE-TTM$_2$** and corresponding peak fitting at 465 nm and 710 nm, respectively.

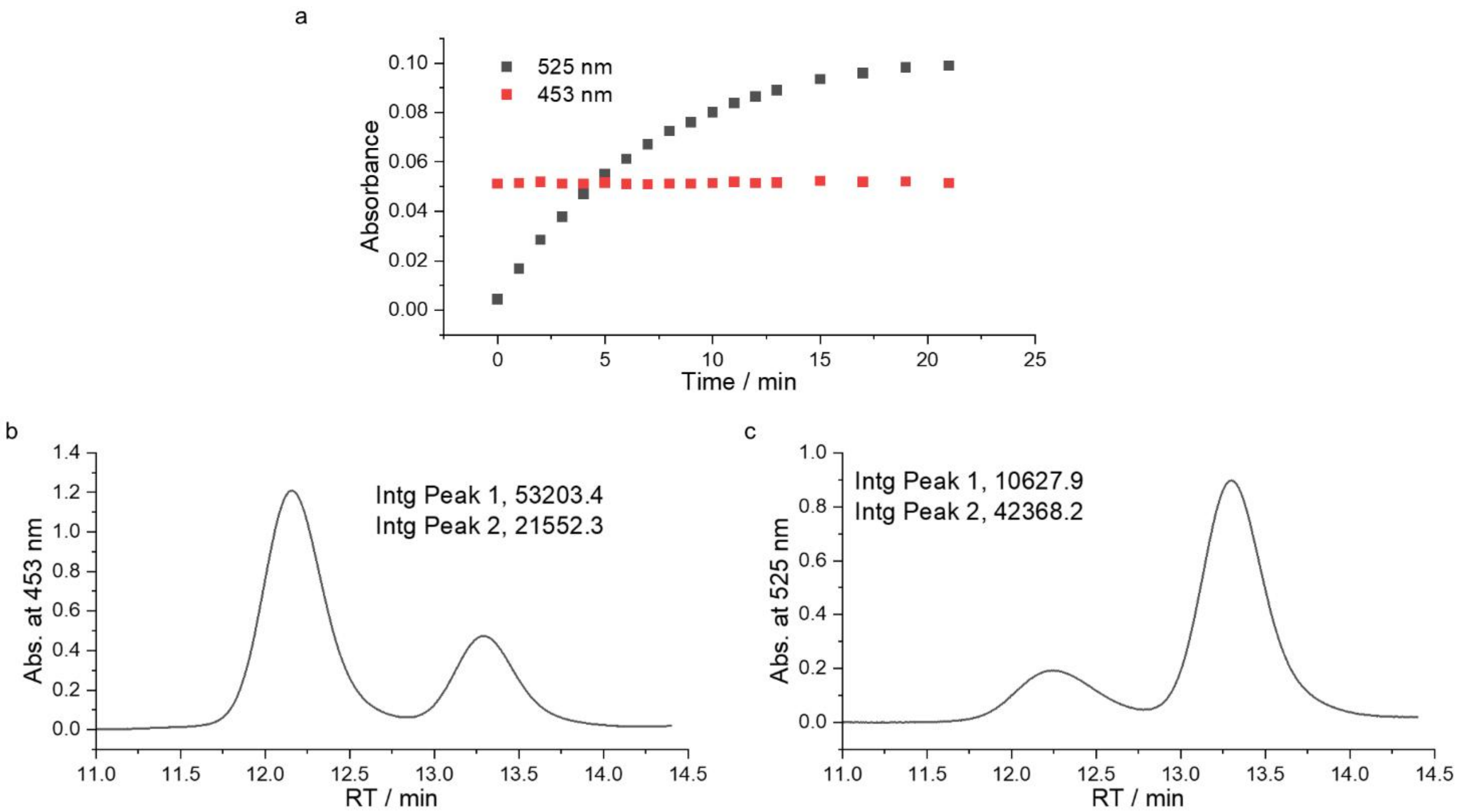

Figure S25 a) Time-dependent absorbance changes associated with the open-to-closed conversion of **F_6-DTE-TTM$_2$** monitored at 525 and 453 nm. b and c) Extracted HPLC traces of **F_6-DTE-TTM$_2$** and corresponding peak fitting at 453 nm and 525 nm, respectively.

8. EPR measurements

8.1 Room-Temperature EPR

To monitor the EPR spectra of two diradicaloids during the conversion from the open to the closed form, EPR measurements of **DTE-TTM$_2$** and **F$_6$-DTE-TTM$_2$** in degassed toluene (c = 1.0 × 10^{-4} M) were performed under 430 nm (power: 12 W) irradiation, with spectra recorded at 1 min intervals. The thermally induced conversion from the closed form back to the open form were performed upon heating at 343 K.

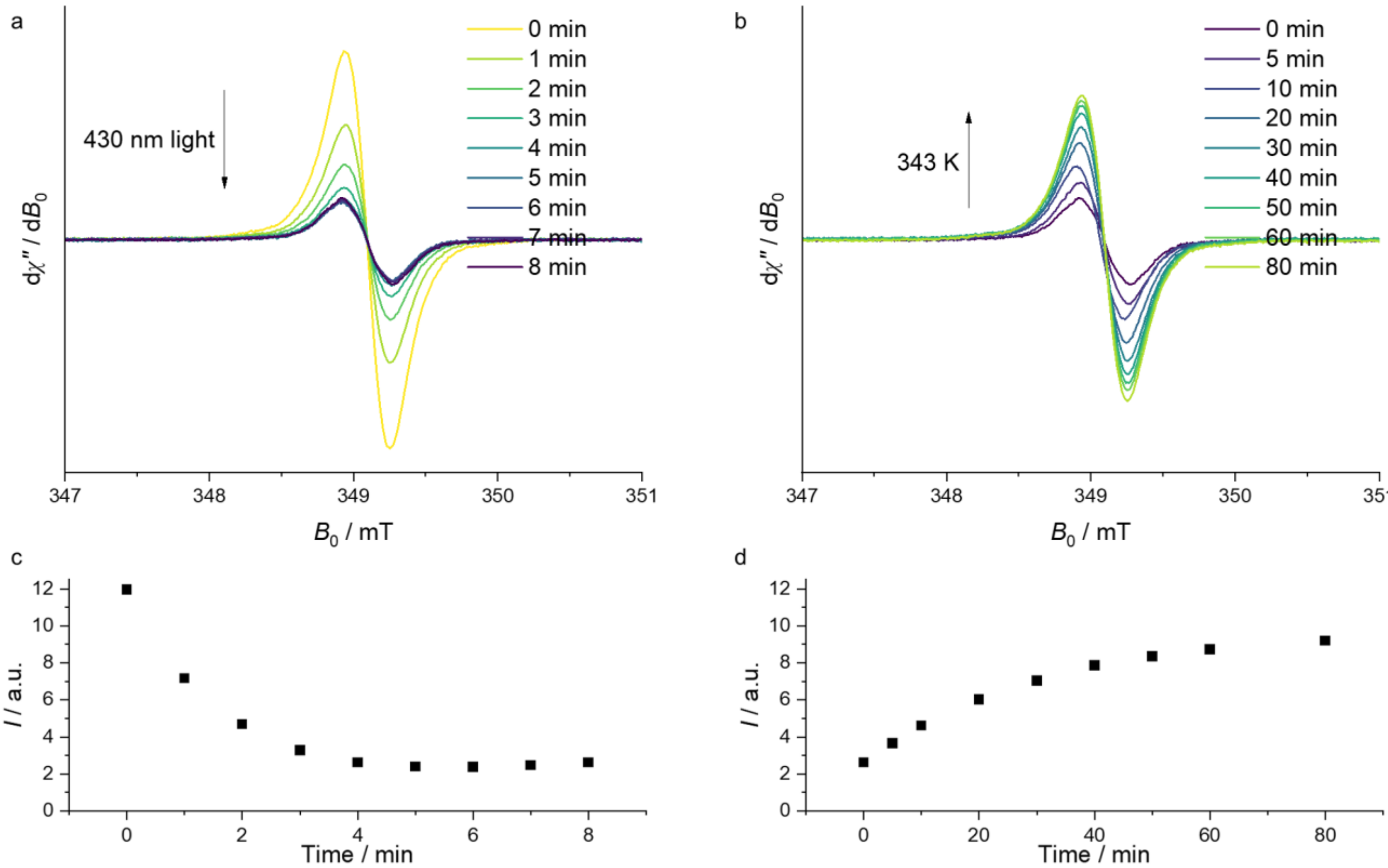

Figure S26 EPR of **o-DTE-TTM$_2$** upon irradiation at 430 nm (a) and the corresponding peak-to-peak intensity kinetics (c), and EPR of **c-DTE-TTM$_2$** upon heating at 343 K (b) and the corresponding peak-to-peak intensity kinetics (d), recorded at room temperature.

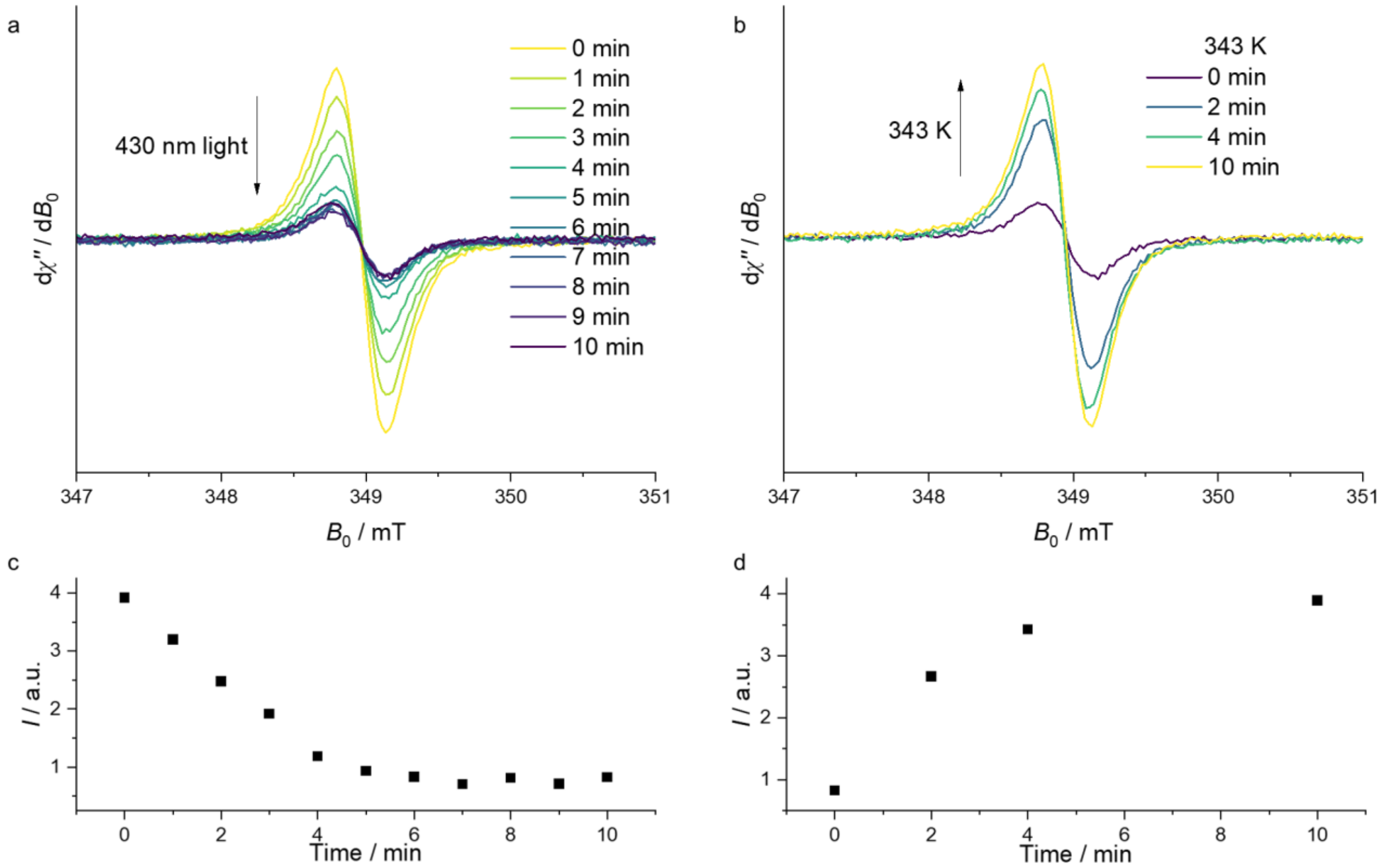

Figure S27 EPR of **o-F$_6$-DTE-TTM$_2$** upon irradiation at 430 nm (a) and the corresponding peak-to-peak intensity kinetics (c), and EPR of **c-F$_6$-DTE-TTM$_2$** upon heating at 343 K (b) and the corresponding peak-to-peak intensity kinetics (d), recorded at room temperature.

Room-temperature EPR spectra of **o-F$_6$-DTE-TTM$_2$** and irradiated **F$_6$-DTE-TTM$_2$** were recorded at concentrations ranging from 10 to 300 μM. The spectra retain the same overall shape across this concentration range, and the EPR signal intensity shows a linear dependence on concentration. These results rule out significant aggregation of the compound within the investigated concentration range.

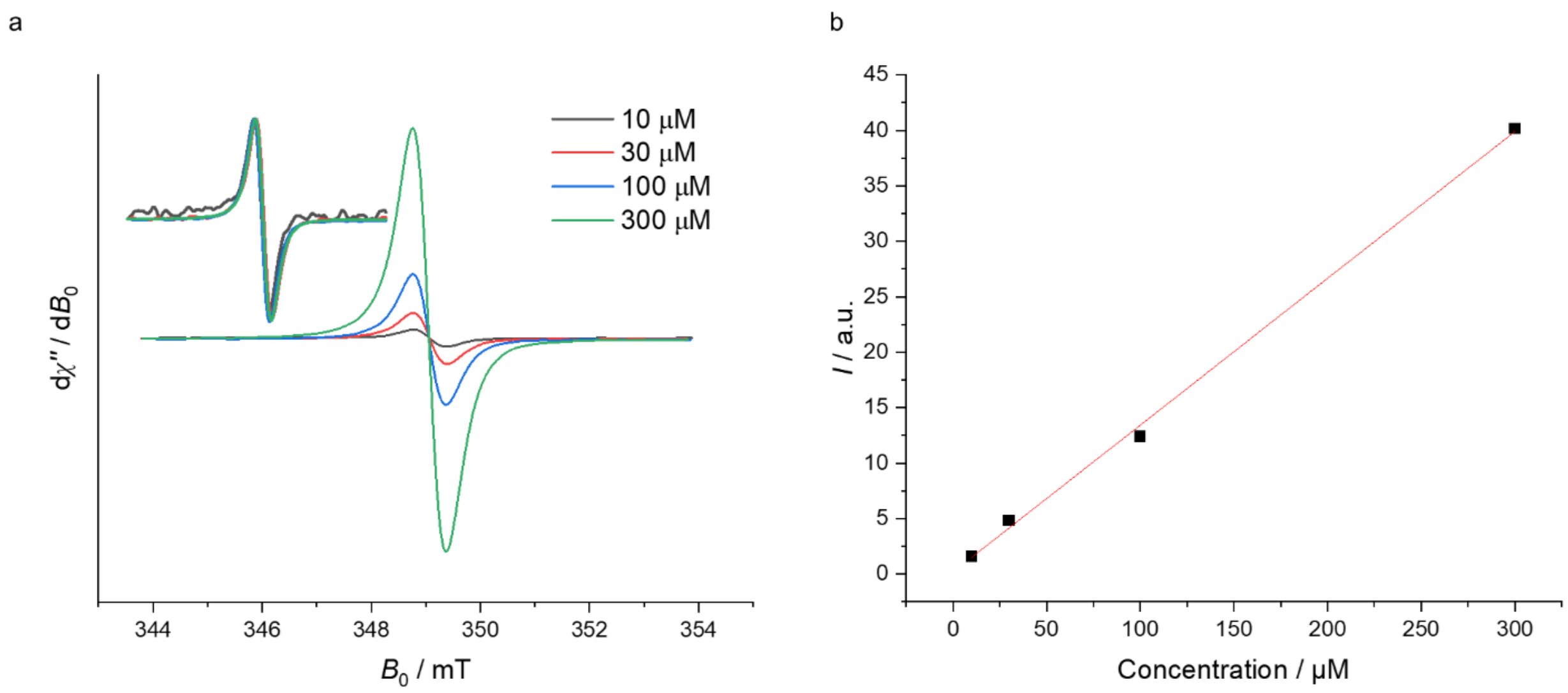

Figure S28 a) EPR of **o-F$_6$-DTE-TTM$_2$** at concentrations from 10 μM to 300 μM. b) Linear fit of the EPR peak-to-peak intensity versus concentration.

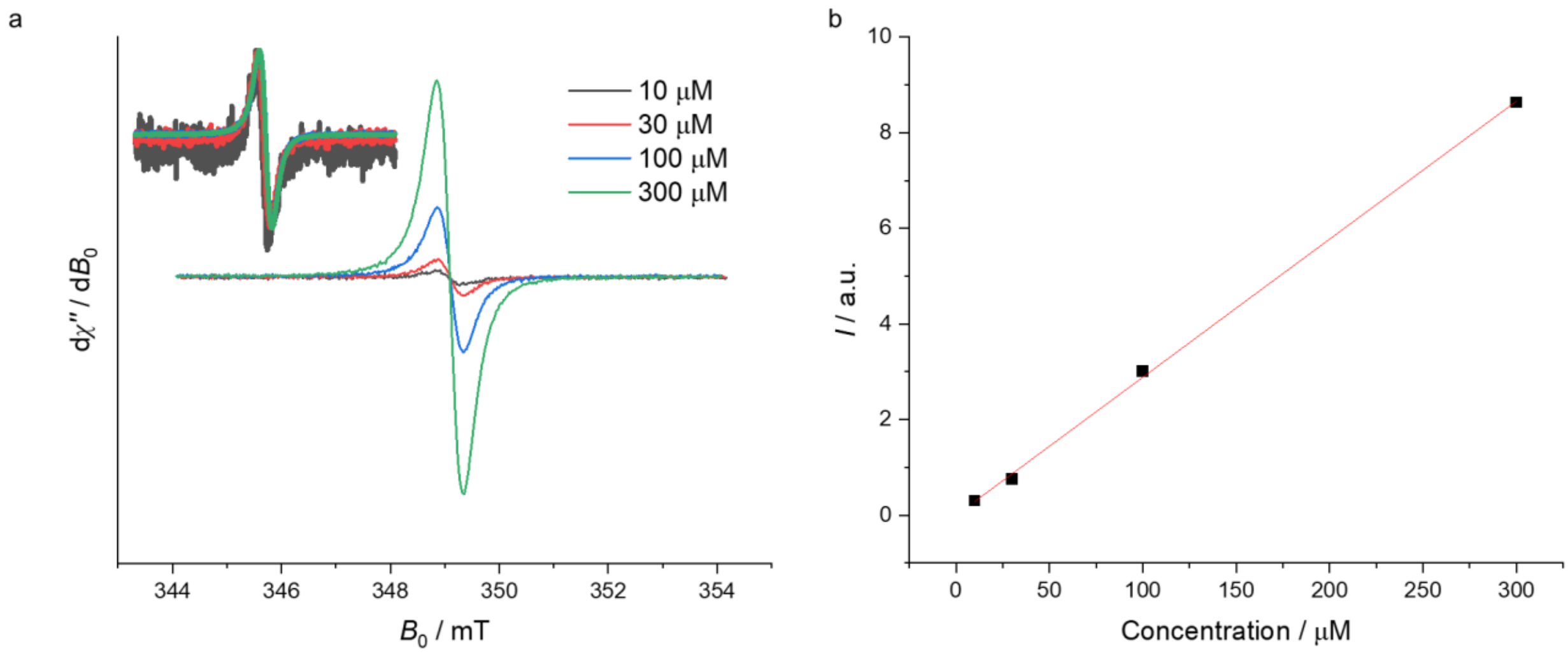

Figure S29 a) EPR of **irradiated F$_6$-DTE-TTM$_2$** at concentrations from 10 μM to 300 μM. b) Linear fit of the EPR peak-to-peak intensity versus concentration.

8.2 VT- EPR of two switches in both open- and closed- forms

VT-EPR Measurements were performed on approx. 0.1 mM solutions of the respective diradicaloid molecule in toluene. The sample was rapidly frozen in liquid nitrogen and quickly inserted into the precooled cryostat. Closed-form samples were prepared by taking out the open-form sample and irradiating with 490 nm (**DTE-TTM$_2$**) and 430 nm (**F$_6$-DTE-TTM$_2$**) light, respectively, until no further changes were observed in the UV–vis absorption spectra. The UV-Vis spectra were recorded on a sample solution of the same batch which was illuminated in a UV-Vis cuvette (1 mm pathlength) alongside the sample inside the EPR tube. From these spectra, the absorption ratios of 525 nm to 465 nm for **F$_6$-DTE-TTM$_2$** and 710 nm to 452 nm for **DTE-TTM$_2$** were obtained. According to the PSD calculations described in Section 7.4 of the SI, the closed-form contents of the samples used for the measurements were estimated to be 75% for **DTE-TTM$_2$** and 94% for **F$_6$-DTE-TTM$_2$**.

Simulations of the spectra obtained in frozen toluene solution at 5 K have been simulated, as shown in Figures S32 and S35. Within the frame of the point-dipole-model, the obtained ZFS parameters D of 14.2 MHz, 7.3 MHz, 14.8 MHz and 6.9 MHz correspond to radical-radical distances of 15.4 Å, 19.2 Å, 15.2 Å and 19.6 Å, respectively. Considering the electron delocalization of TTM diradicaloids, these values are in acceptable agreement to the ~17.4 Å obtained through XRD measurements.

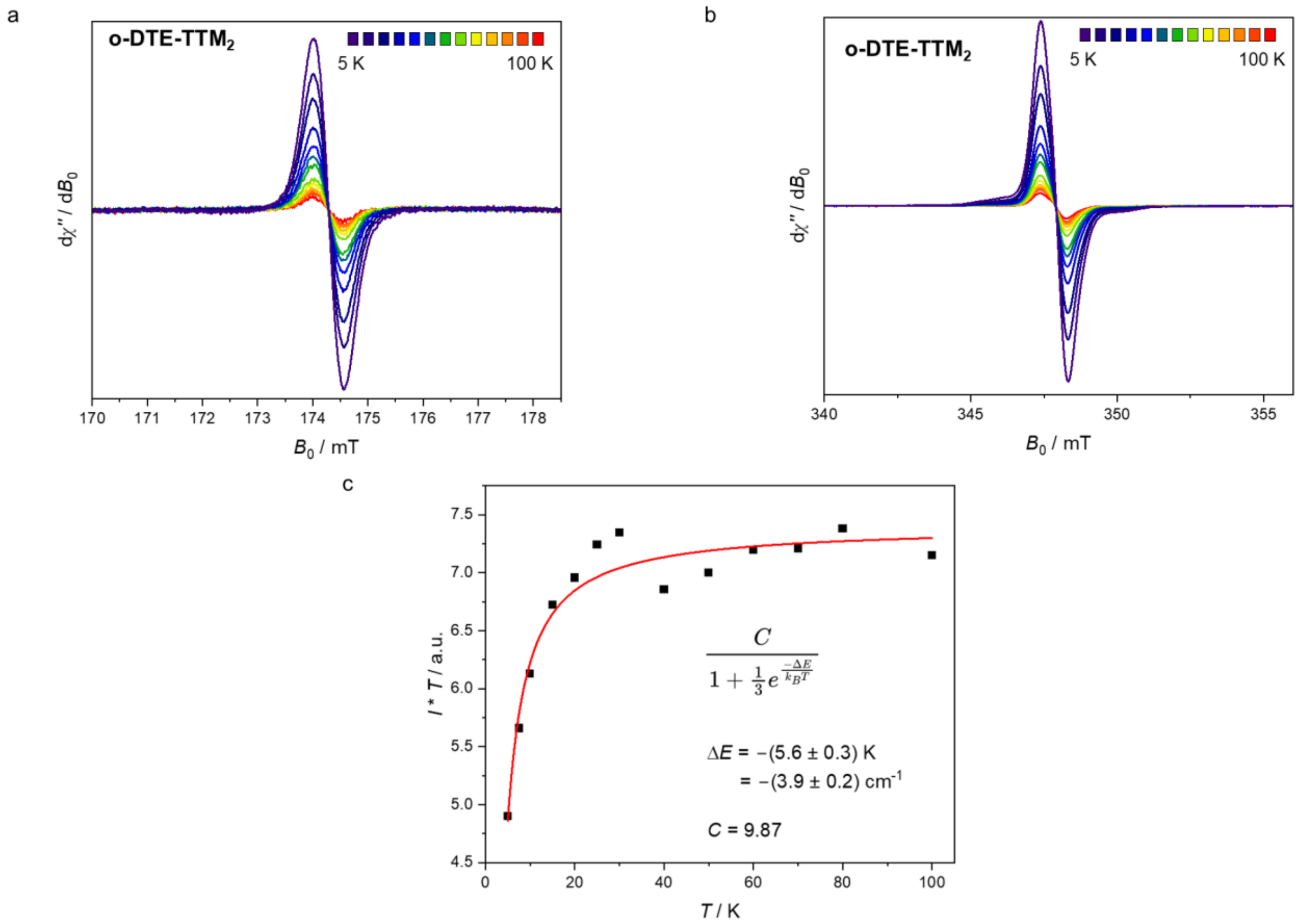

Figure S30 VT-EPR spectra of **o-DTE-TTM$_2$** in toluene recorded from 5 to 100 K in the half-field transition region a) and full-field region b). c) Experimental I*T versus T plots fitted using the Bleaney–Bowers model.

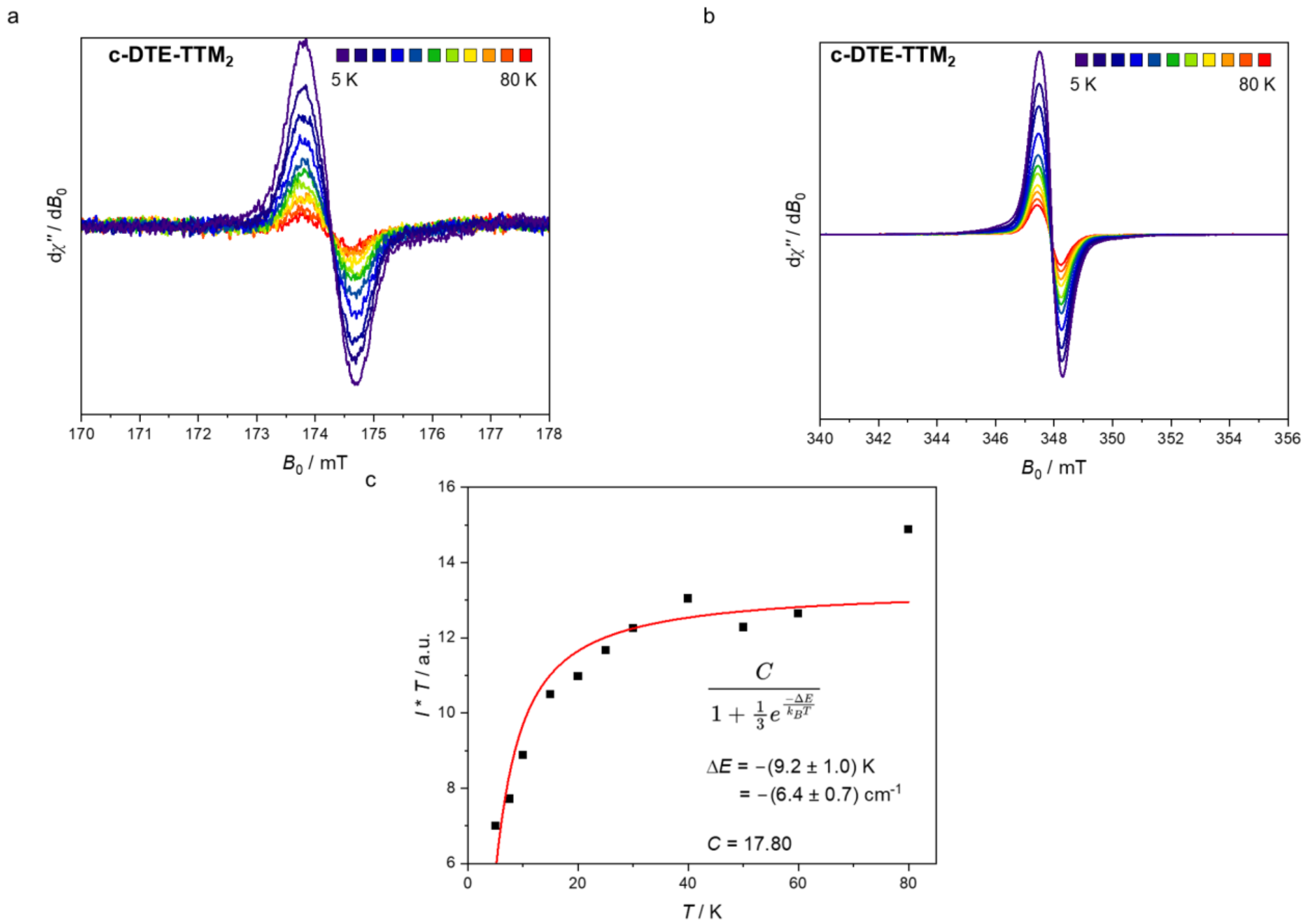

Figure S31 VT-EPR spectra of **c-DTE-TTM$_2$** in toluene recorded from 5 to 80 K in the half-field transition region a) and full-field region b). c) Experimental I*T versus T plots fitted using the Bleaney–Bowers model.

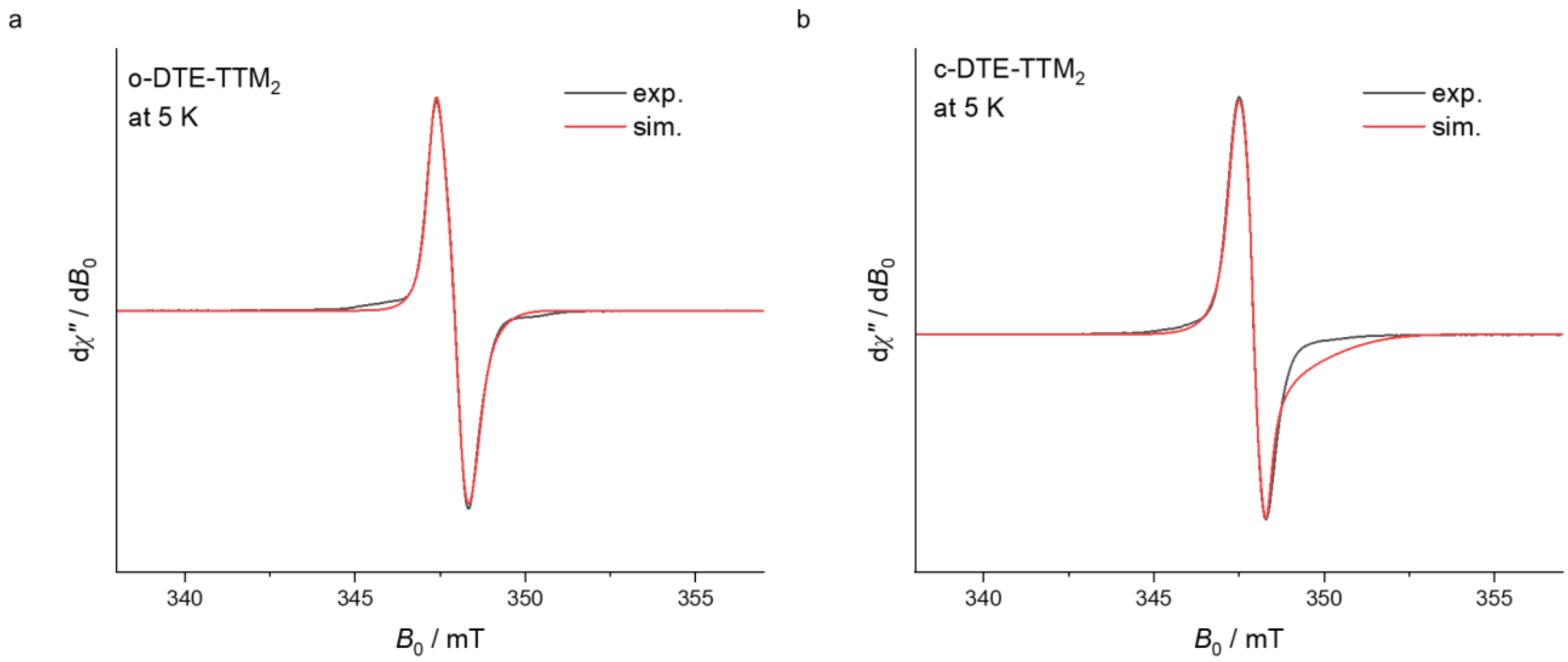

Figure S32 Experimental (black lines) and simulated (red lines) EPR spectra of **o-DTE-TTM$_2$** (a) and **c-DTE-TTM$_2$** (b) in toluene at 5 K.

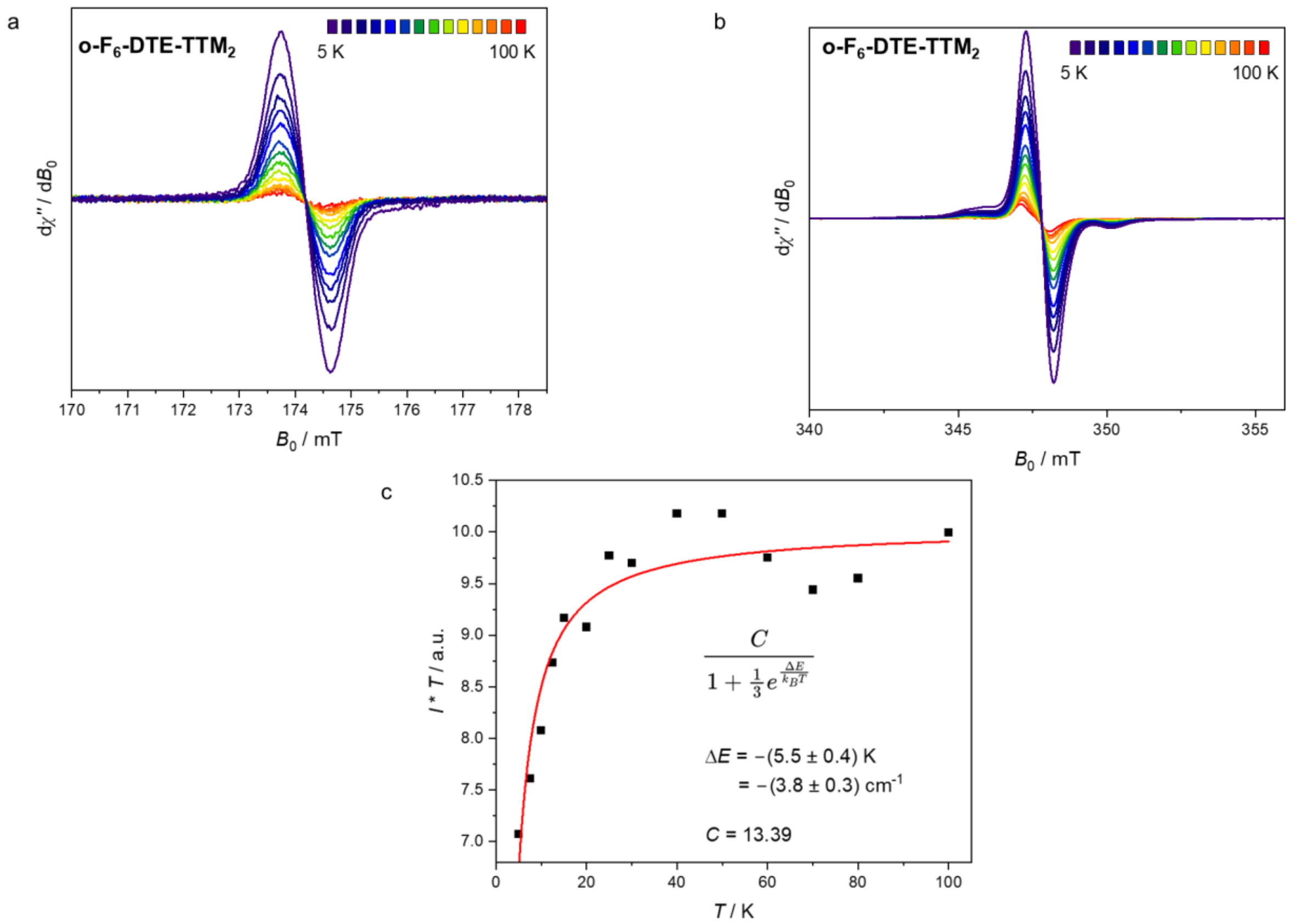

Figure S33 VT-EPR spectra of **o-F_6-DTE-TTM_2** in toluene recorded from 5 to 100 K in the half-field transition region a) and full-field region b). c) Experimental I*T versus T plots fitted using the Bleaney–Bowers model.

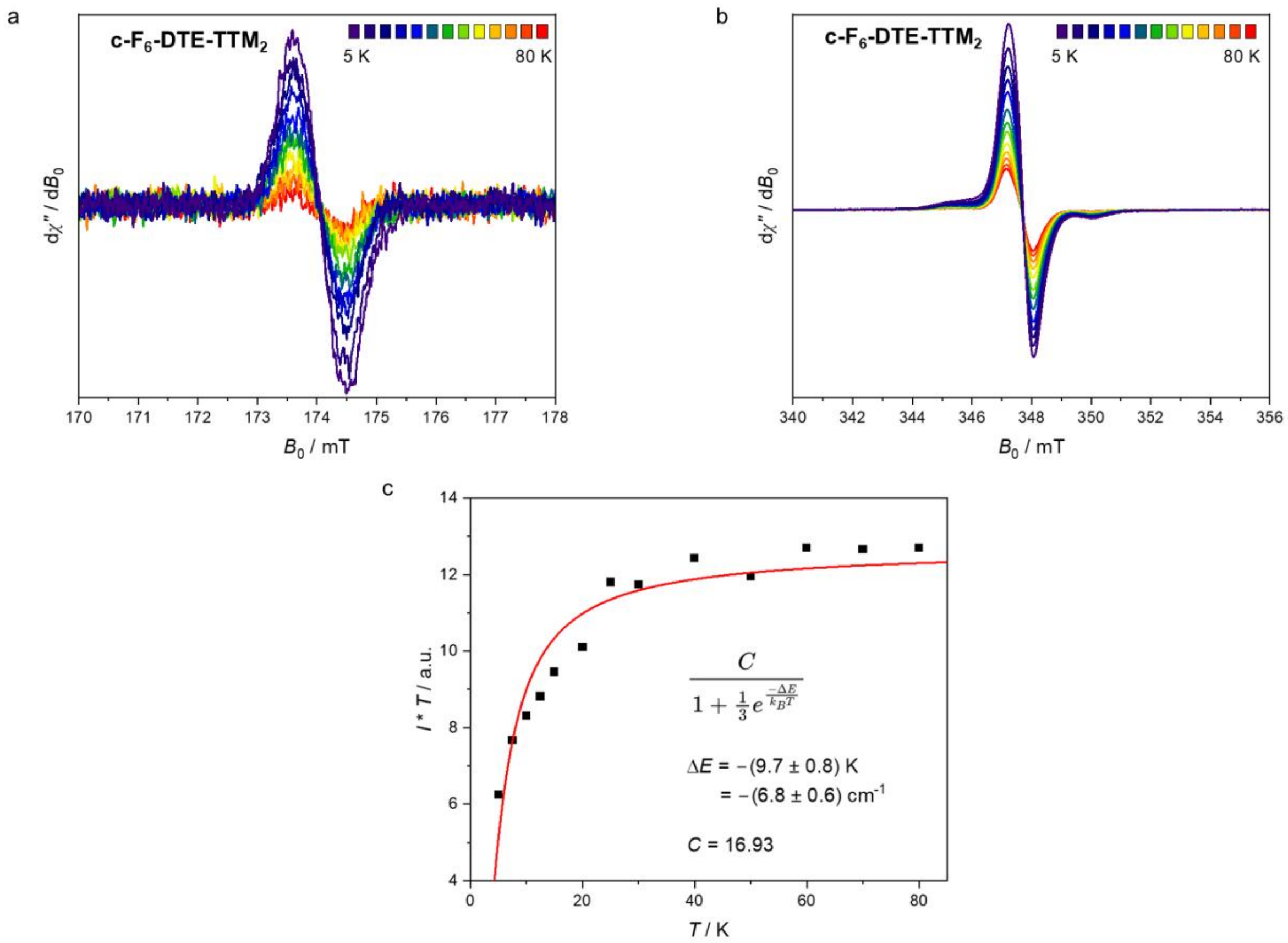

Figure S34 VT-EPR spectra of **c-F$_6$-DTE-TTM$_2$** in toluene recorded from 5 to 80 K in the half-field transition region a) and full-field region b). c) Experimental **I***T versus T plots fitted using the Bleaney–Bowers model.

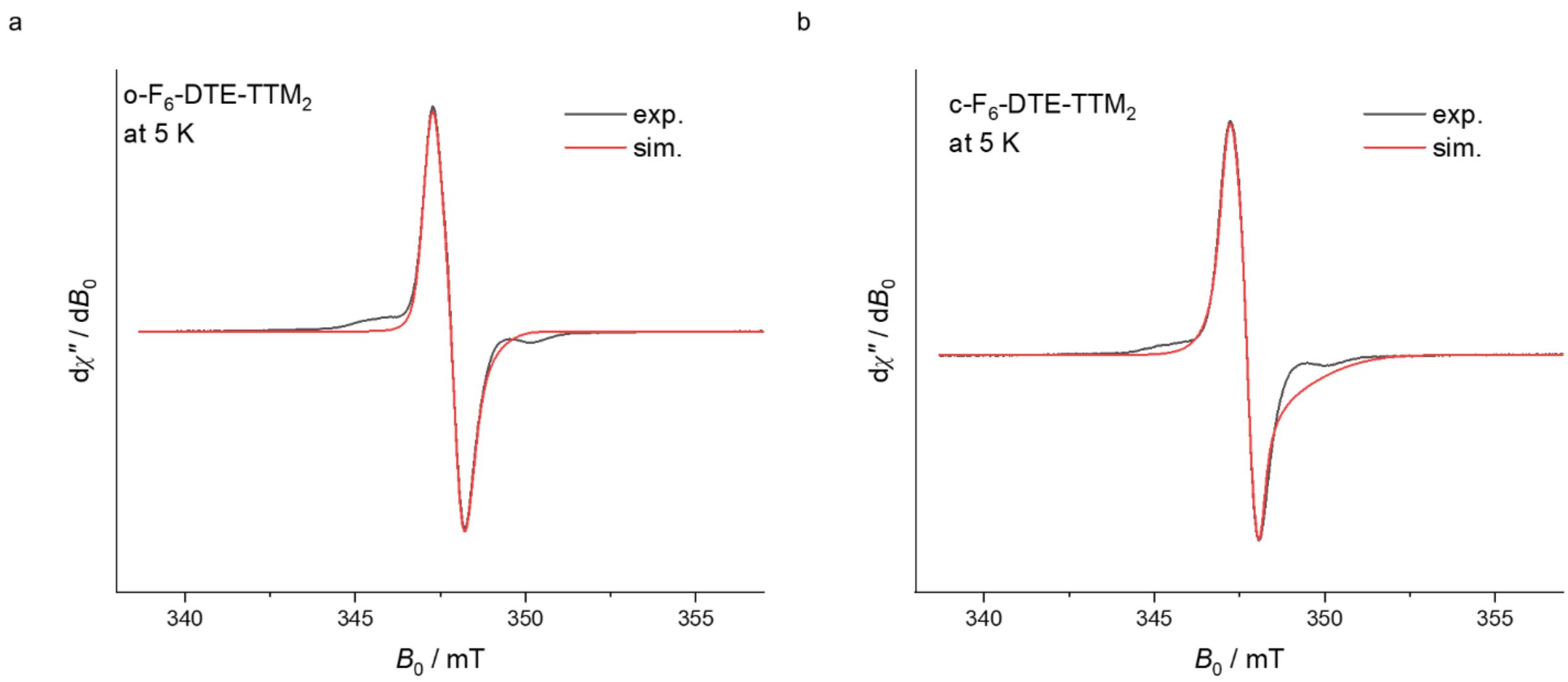

Figure S35 Experimental (black lines) and simulated (red lines) EPR spectra of **o-F$_6$-DTE-TTM$_2$** (a) and **c-F$_6$-DTE-TTM$_2$** (b) in toluene at 5 K.

9. DFT calculations

All quantum chemical calculations were performed using ORCA 6.0.0. [8] The geometry optimizations and the single-point calculations were carried out using the unrestricted B3LYP and M06-2X functionals at the def2-TZVP level of theory together with dispersion correction using the Becke-Johnson damping scheme (D3BJ) and the zero damping scheme (D3zero), respectively.[9-13]

We note that the B3LYP functional suffers from significant spin contamination when calculating the closed-form compounds, leading to substantial errors in both the singlet-triplet gap ΔE_{ST} and the diradical character y_0. By employing the M06-2X functional, the spin contamination was successfully minimized, yielding ΔE_{ST} values that qualitatively agree with the experimental VT-EPR results.
To evaluate the qualitative electronic description of the frontier orbitals manifolds, the B3LYP functional was retained for the molecular orbital analysis. While spin contamination is known to shift absolute unrestricted eigenvalues, the spatial topologies and relative energy trends of the resulting orbitals remain robust. Crucially, the B3LYP calculated SOMO-SUMO (singly (un)occupied molecular orbitals) gaps closely match the lowest absorption energy of both the open and the closed form obtained from UV-vis spectroscopy. Consequently, a dual-level approach was used, where the spin state properties were derived from the M06-2X functional while B3LYP was exclusively used for qualitative orbital analysis.

The singlet-triplet energy gap (ΔE_{ST}) was calculated with the Yamaguchi spin decontamination correction according to the following equation[14-15]:

$$\Delta E_{\mathrm{ST}} = 2J = -\frac{2(E_{\mathrm{T}} - E_{\mathrm{BS}})}{\langle S^2\rangle_{\mathrm{T}} - \langle S^2\rangle_{\mathrm{BS}}}$$

Here, J corresponds to the intramolecular exchange interaction within the diradicals, E_{T} and E_{BS} represent the total energy of the calculated triplet and broken-symmetry state and $\langle S^2\rangle_{\mathrm{T}}$ and $\langle S^2\rangle_{\mathrm{BS}}$ correspond to the respective expectation value of the total spin squared operator. The diradical character y_0 was calculated from the occupation number of the highest occupied natural orbital (n_{HONO}) and of the lowest unoccupied natural orbital (n_{LUNO}) according to Yamaguchi[16]:

$$y_0 = 1 - \frac{2T_0}{1 + T_0^2}$$

$$T_0 = \frac{n_{\mathrm{HONO}} - n_{\mathrm{LUNO}}}{2}$$

y_0 can be can be rather different and functional dependent.[17-18] Here we included y_0 obtained from M06-2X and B3LYP as shown in **Tables S2 and S3.**
Geometry optimizations of the closed-shell (quinoidal) structures of **c-DTE-TTM$_2$** and **c-DTE-F$_6$-TTM$_2$** reveal that they exhibit a significant higher planarity than their corresponding open-shell (diradical) counterparts. (Figures S39-S41) Consequently, we conclude that a representative diradical character y_0 cannot be reliably determined using the standard single-point method. Because this approach relies exclusively on the optimized open-shell geometry, it fails to provide a representative value for y_0 when the

system undergoes profound structural relaxation i.e. pronounced planarization as observed here, upon shifting between the open- and closed-shell potential energy surfaces.

Table S2: Calculated energies and properties of o-DTE-TTM$_2$ and c-DTE-TTM$_2$

	M06-2X			B3LYP		
	**o-DTE-TTM$_2$**	**c-DTE-TTM$_2$** (open-shell)	**c-DTE-TTM$_2$** (closed-shell)	**o-DTE-TTM$_2$**	**c-DTE-TTM$_2$** (open-shell)	**c-DTE-TTM$_2$** (closed-shell)
E_T (Eh)	-10194.7705	-10194.7482	-	-10193.9062	-10193.8883	-
E_S (Eh)	-10194.7705	-10194.7484	-10194.74638	-10193.9062	-10193.8907	-10193.8894
ΔE_{ST} (kcal/mol) [K]	-0.0009 [0.45]	-0.088 [44.3]	-	-0.0041 [20.6]	-0.896 [450.9]	-
$<S^2>_T$	2.033	2.034	-	2.045	2.048	-
$<S^2>_{BS}$	1.033	1.043	-	1.045	1.113	-
y_0	0.99	0.96	-	0.98	0.35	-

Table S3: Calculated energies and properties of o-F$_6$-DTE-TTM$_2$ and c-F$_6$-DTE-TTM$_2$

	M06-2X			B3LYP		
	**o-F$_6$-DTE-TTM$_2$**	**c-F$_6$-DTE-TTM$_2$** (open-shell)	**c- F$_6$-DTE-TTM$_2$** (closed-shell)	**o-F$_6$-DTE-TTM$_2$**	**c-F$_6$-DTE-TTM$_2$** (open-shell)	**c-F$_6$-DTE-TTM$_2$** (closed-shell)
E_T (Eh)	-10790.3055	-10790.2793	-	-10789.4204	-10789.3995	-
E_S (Eh)	-10790.3055	-10790.2794	-10790.2718	-10789.4204	-10789.4009	-10789.3963
ΔE_{ST} (kcal/mol) [K]	-0.0009 [0.45]	-0.073 [36.7]	-	-0.0037 [1.86]	-0.688 [346.2]	-
$<S^2>_T$	2.032	2.033	-	2.044	2.042	-
$<S^2>_{BS}$	1.032	1.041	-	1.044	1.105	-
y_0	0.99	0.96	-	0.98	0.65	-

o-DTE-TTM$_2$

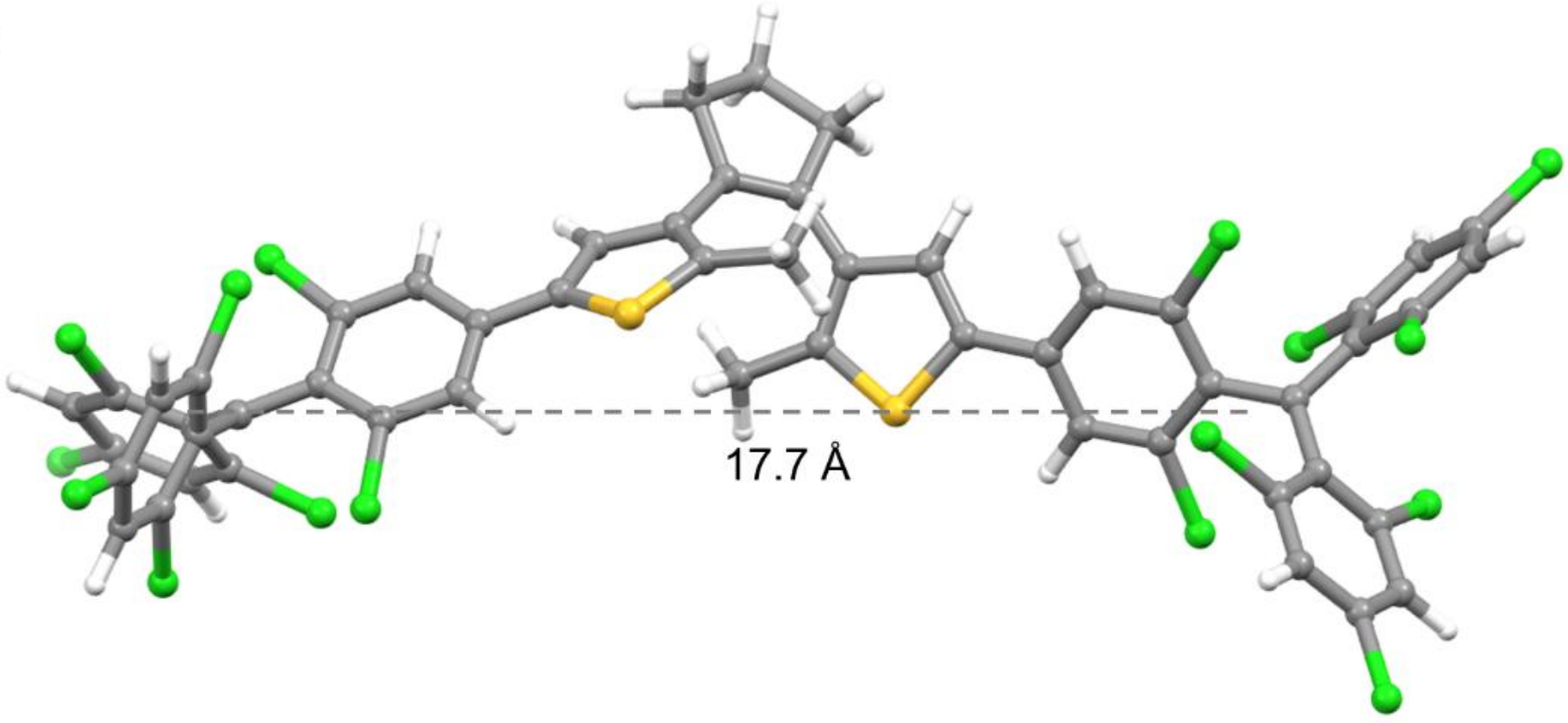

Figure S36 DFT-optimized structure of **o-DTE-TTM$_2$** (*UM06-2X/def2-TZVP D3zero*)

o-F$_6$-DTE-TTM$_2$

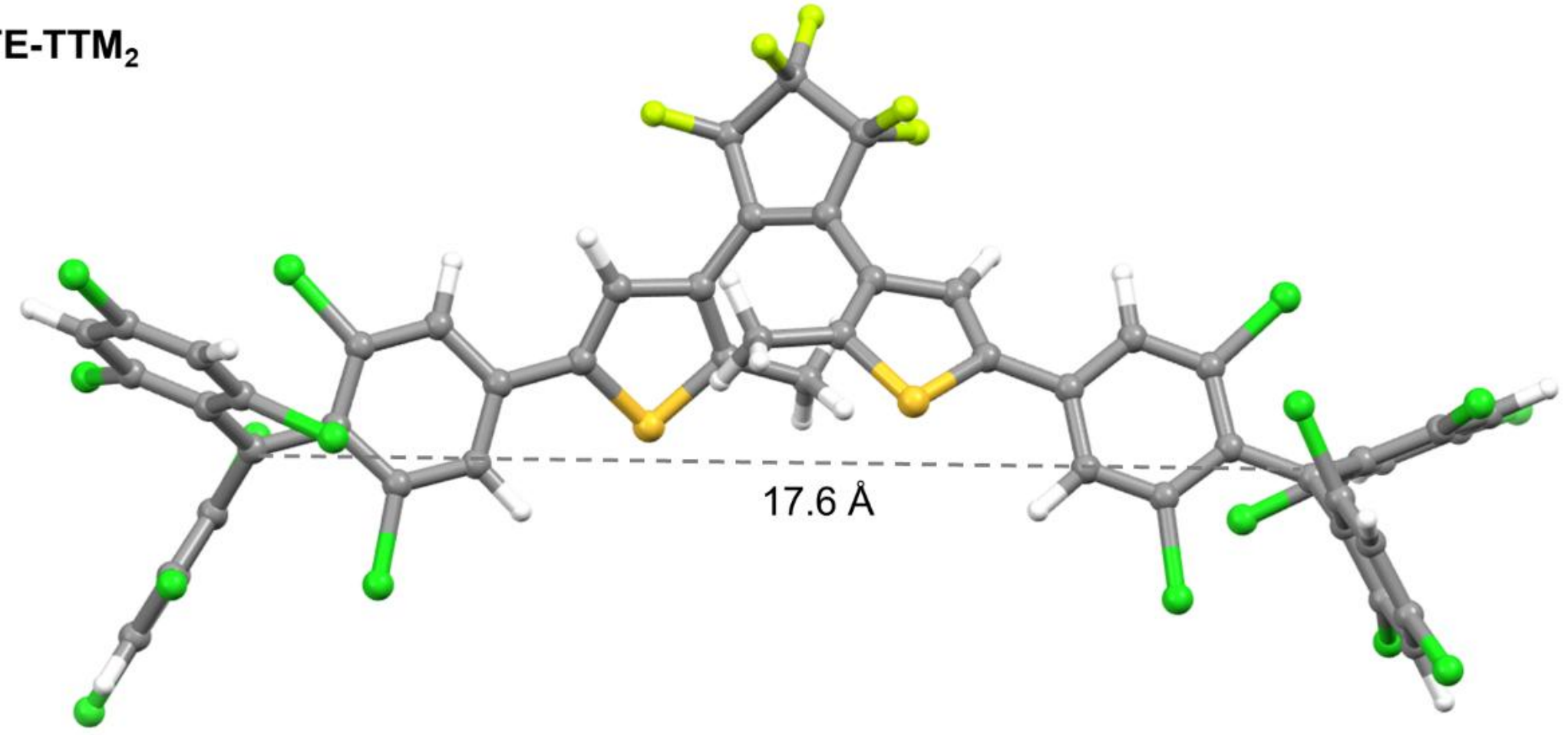

Figure S37 DFT-optimized structure of **o-F$_6$-DTE-TTM$_2$** (*UM06-2X/def2-TZVP D3zero*)

c-DTE-TTM$_2$ (Open shell)

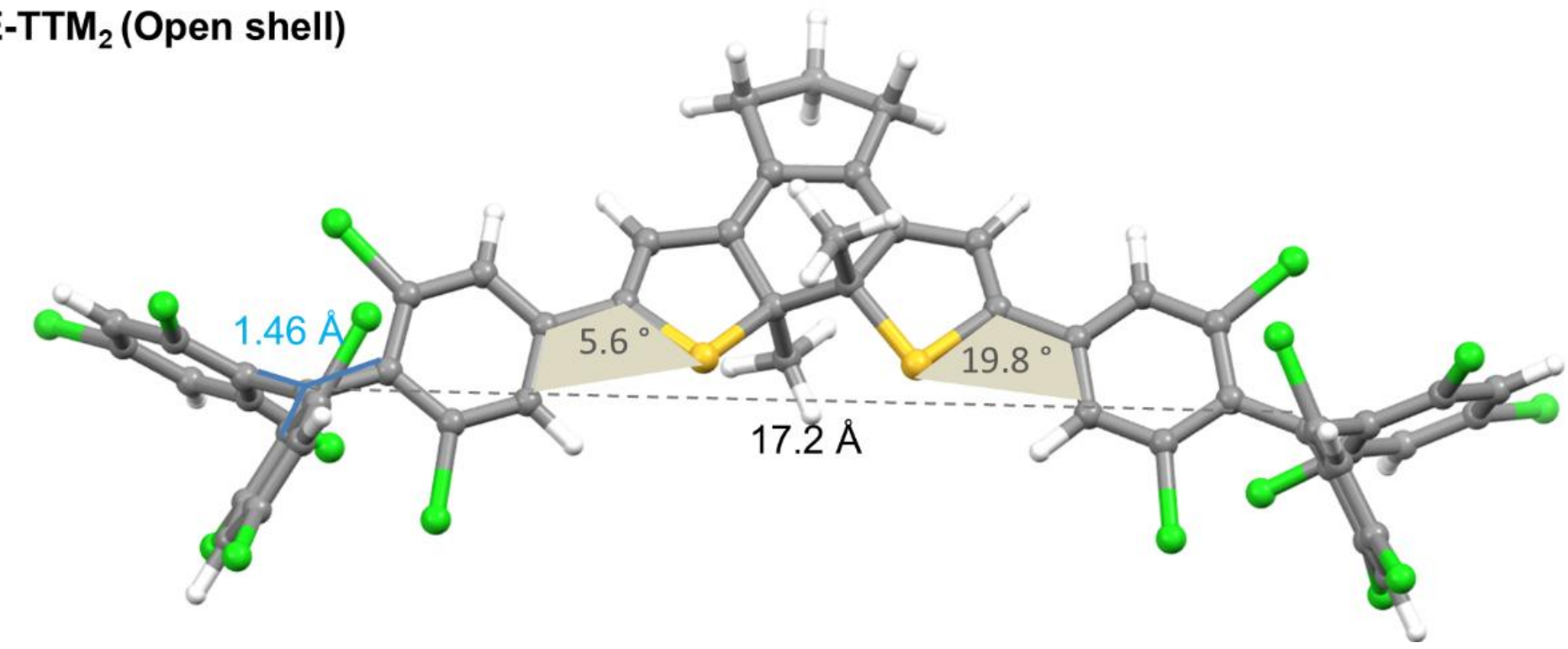

Figure S38 DFT-optimized structure of **c-DTE-TTM$_2$** in open shell (*UM06-2X/def2-TZVP D3zero*)

c-DTE-TTM$_2$ (Closed shell)

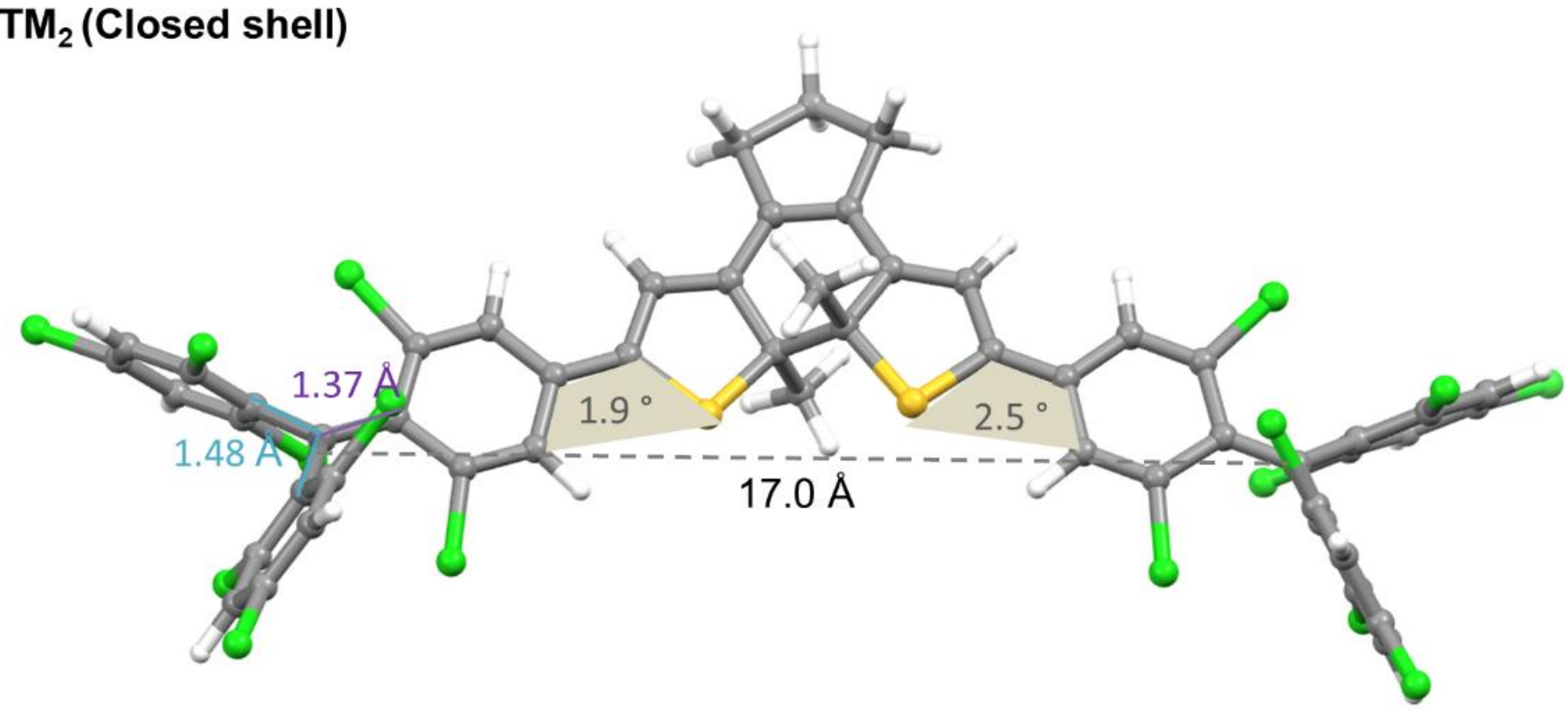

Figure S39 DFT-optimized geometries of **c-DTE-TTM$_2$** in close shell with key bond lengths and angels

c-F$_6$-DTE-TTM$_2$ (Open shell)

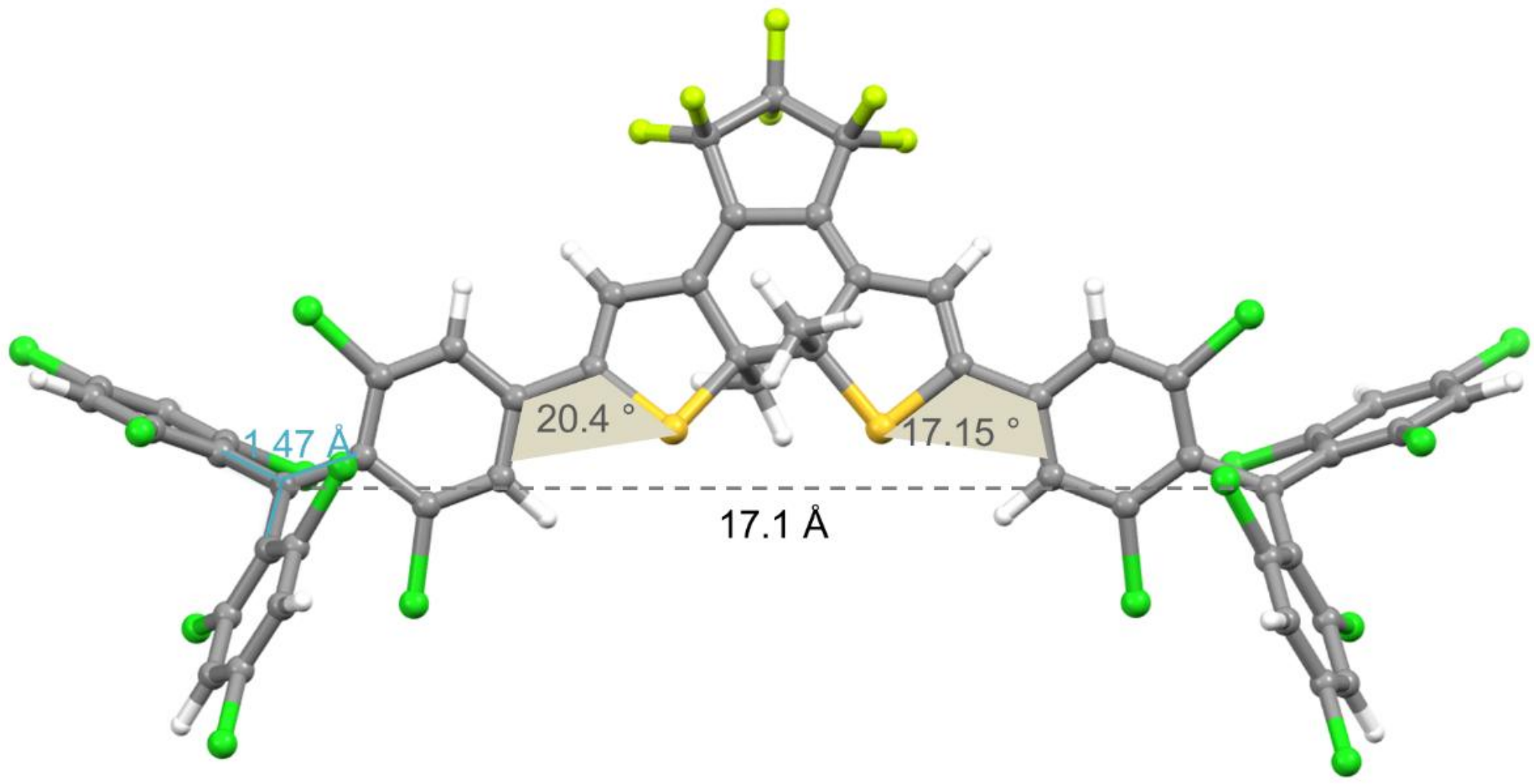

Figure S40 DFT-optimized geometries of **c-F$_6$-DTE-TTM$_2$** in open shell with key bond lengths and angels

c-F$_6$-DTE-TTM$_2$ (Closed shell)

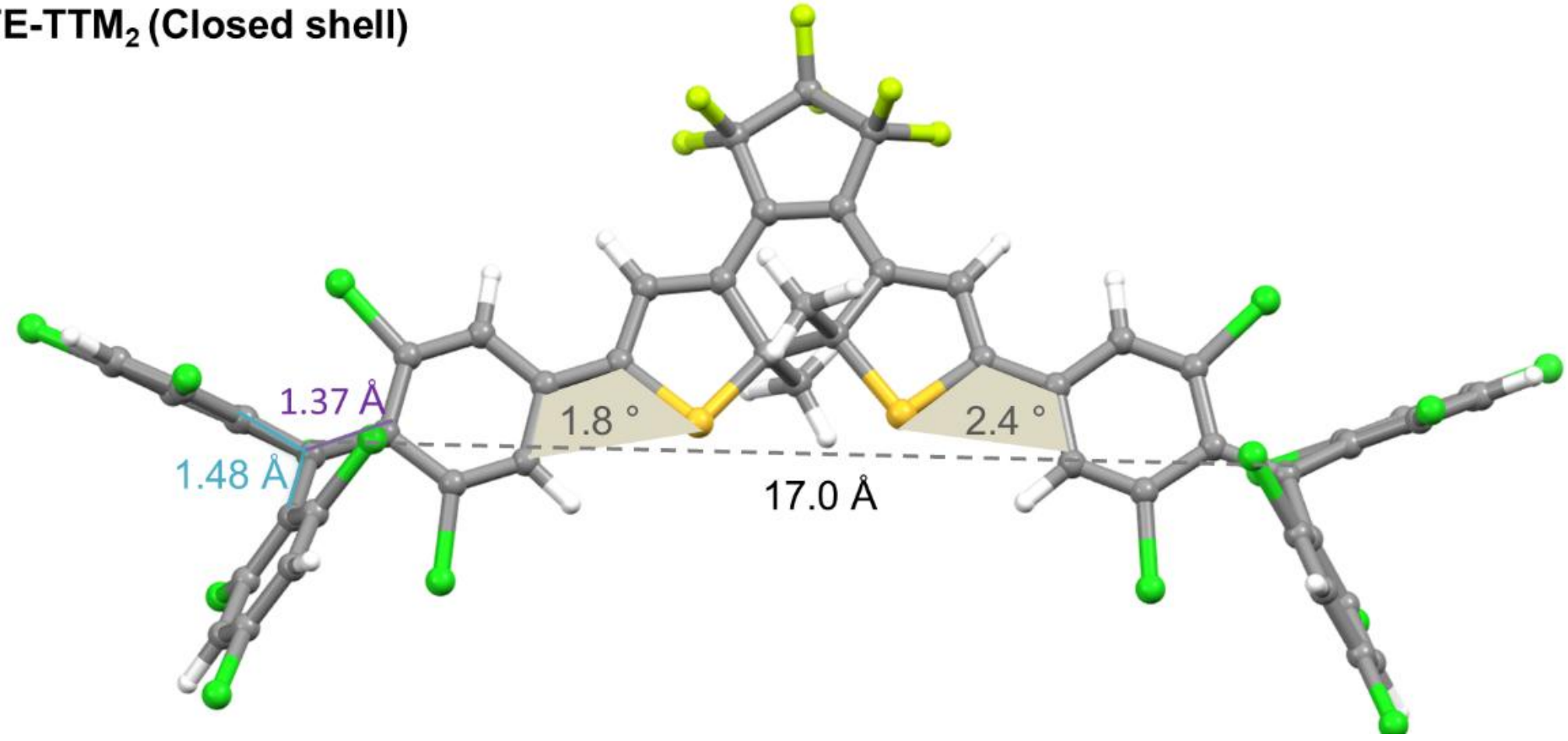

Figure S41 DFT-optimized geometries of **c-F$_6$-DTE-TTM$_2$** in close shell with key bond lengths and angels

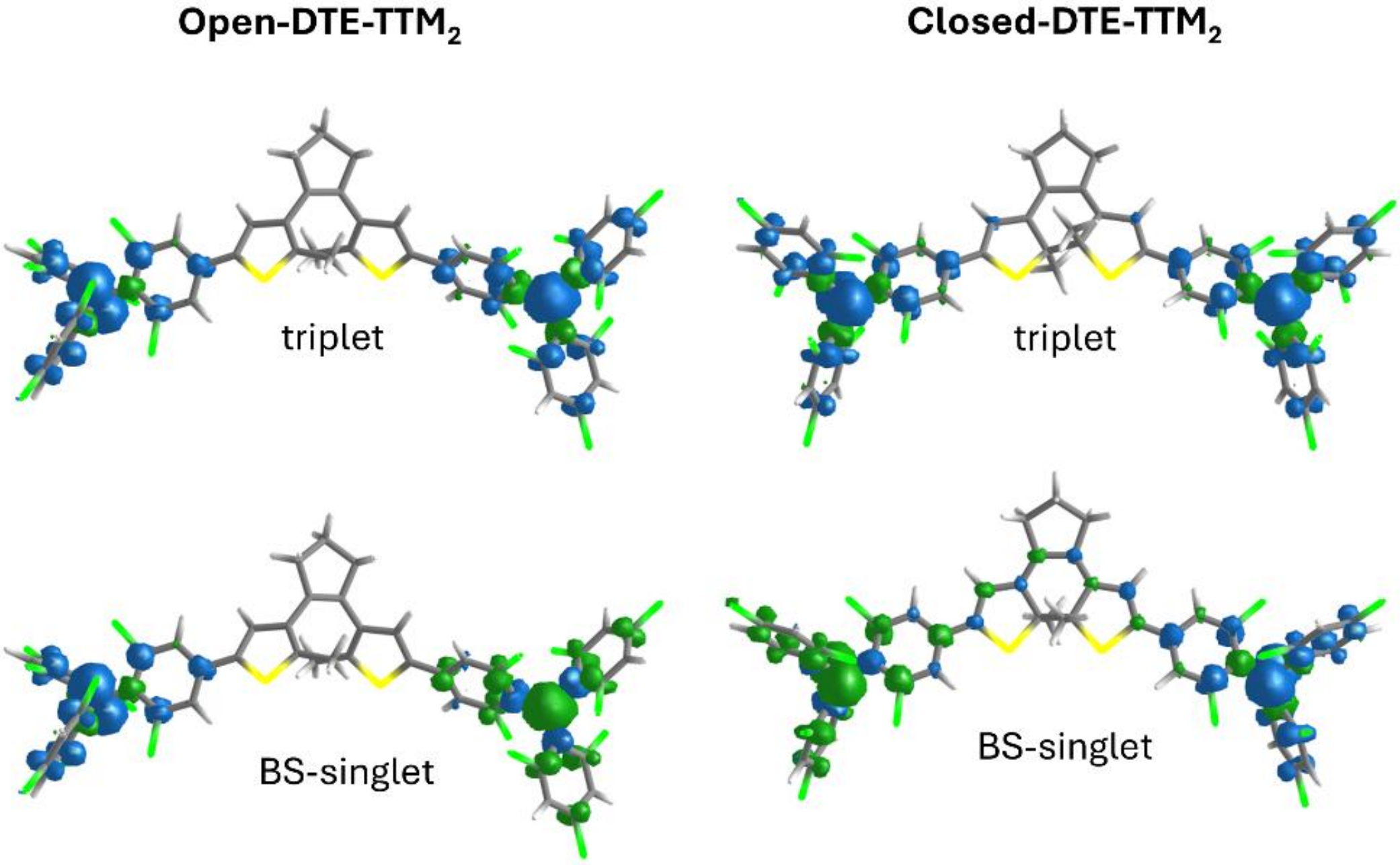

Figure S42 Spin density of **DTE-TTM$_2$** (*Geometry optimization: UM06-2X/def2-TZVP D3zero, isovalue: 0.003*)

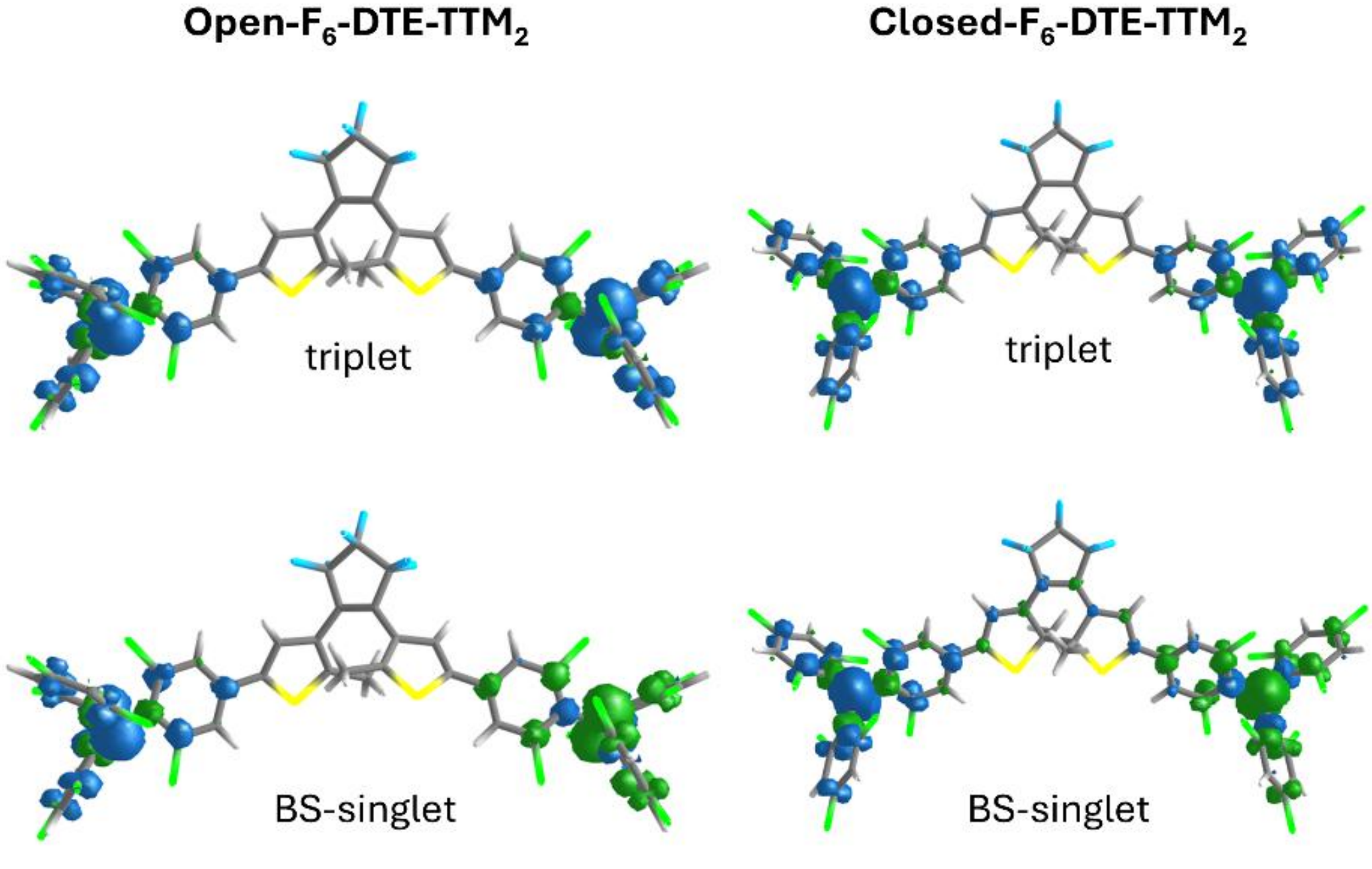

Figure S43 Spin density of **F$_6$-DTE-TTM$_2$** (*Geometry optimization: UM06-2X/def2-TZVP D3zero, isovalue: 0.003*)

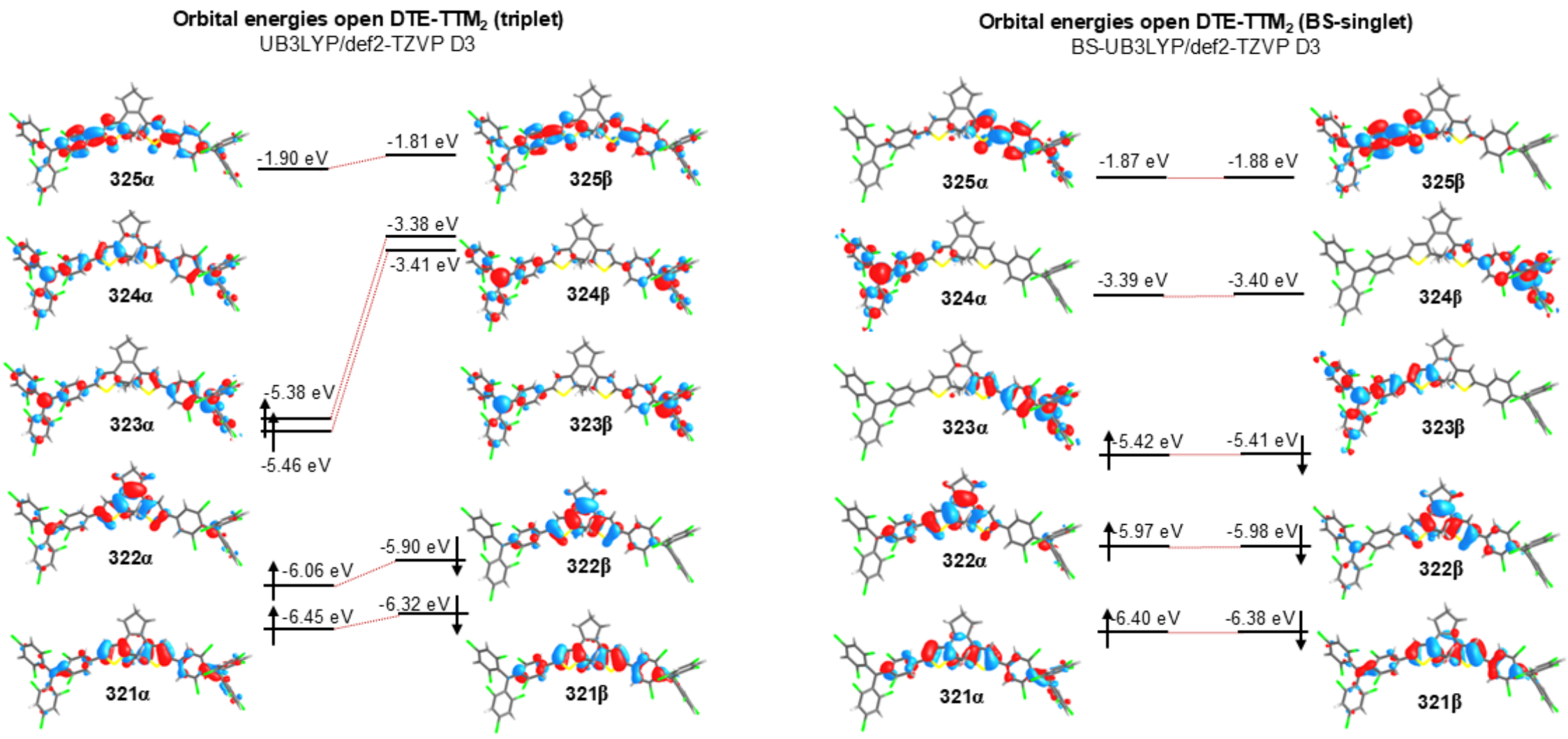

Figure S44 Orbital energies of **o-DTE-TTM$_2$** (triplet) and **o-DTE-TTM$_2$** (BS singlet) (*Geometry optimization and orbital analysis: UB3LYP/def2-TZVP D3, (isovalue: 0.03)*)

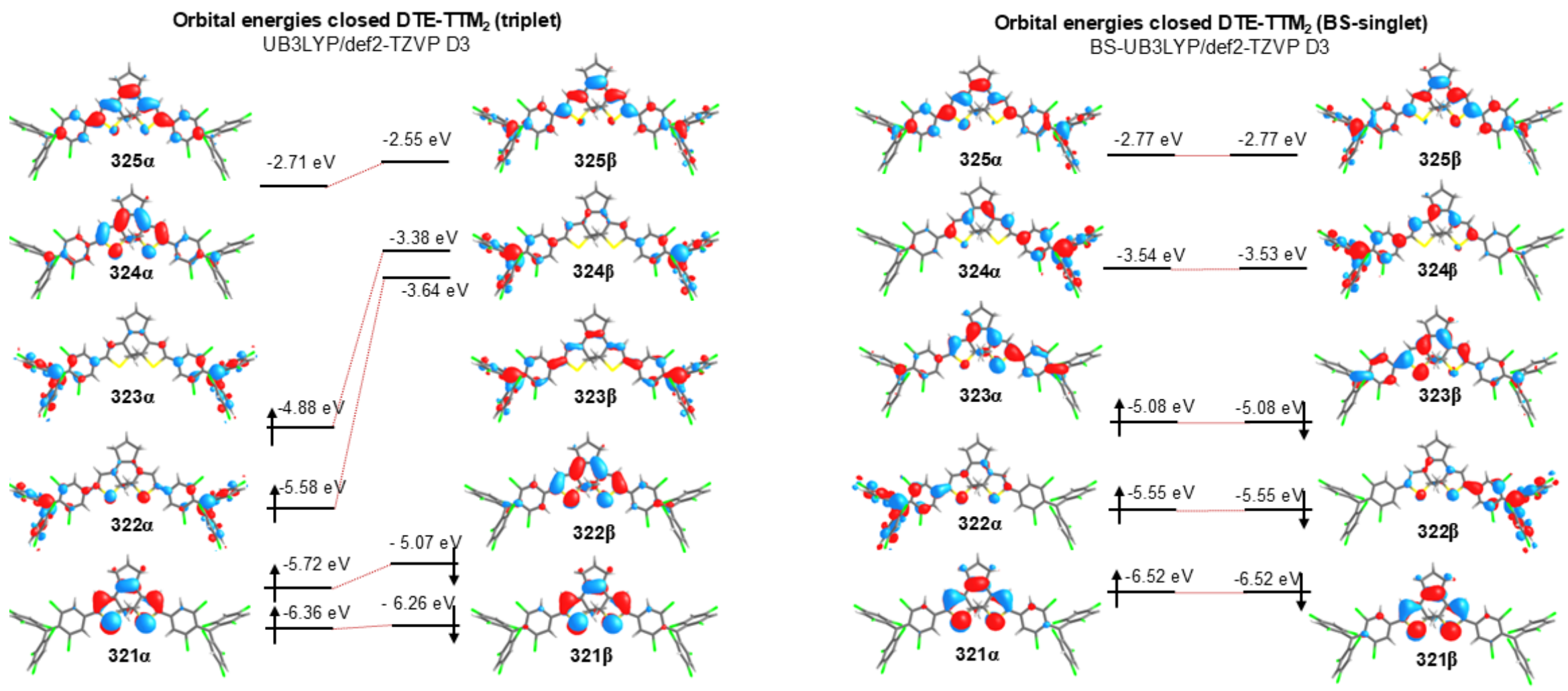

Figure S45 Orbital energies of **c-DTE-TTM$_2$** (triplet) and **c-DTE-TTM$_2$** (BS singlet) (*Geometry optimization and orbital analysis: UB3LYP/def2-TZVP D3, (isovalue: 0.03)*)

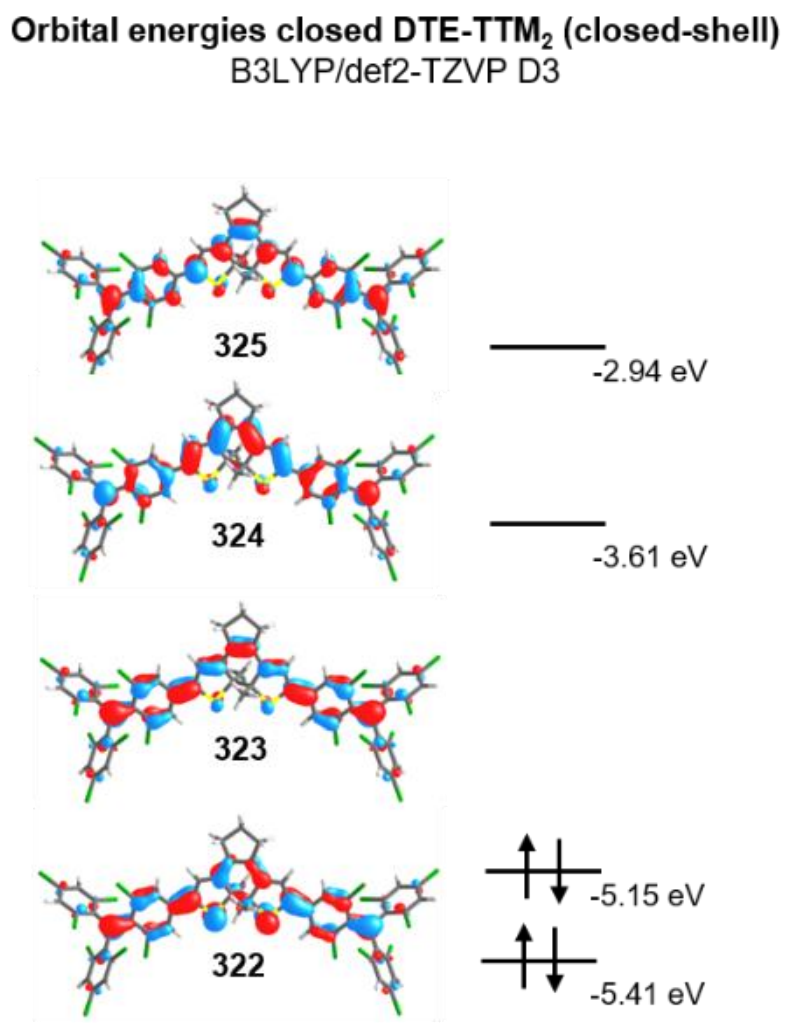

Figure S46 Orbital energies of **c-DTE-TTM₂** (closed-shell, quinoidal) (*Geometry optimization and orbital analysis: B3LYP/def2-TZVP D3, (isovalue: 0.03)*)

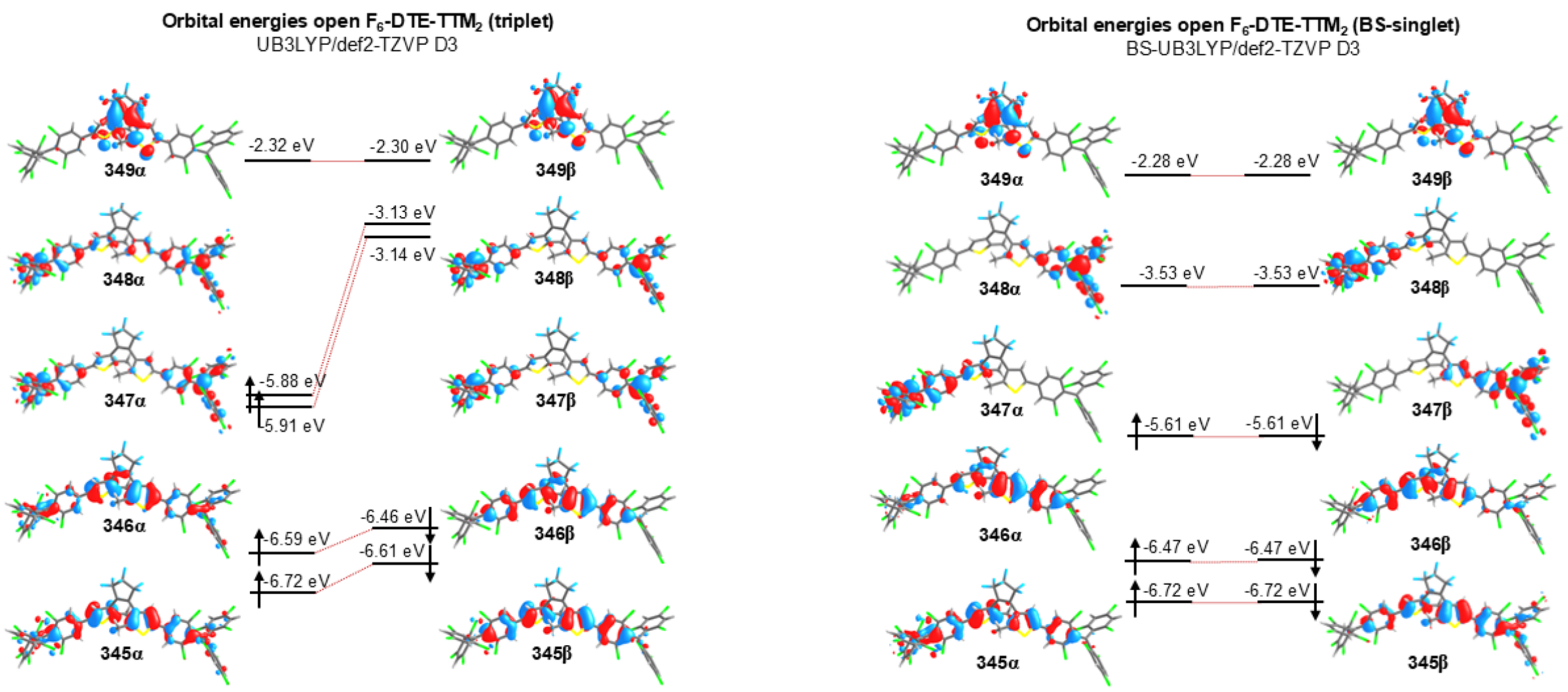

Figure S47 Orbital energies of **o-F$_6$-DTE-TTM$_2$** (triplet) and **o-F$_6$-DTE-TTM$_2$** (BS singlet) (*Geometry optimization and orbital analysis: UB3LYP/def2-TZVP D3, (isovalue: 0.03)*)

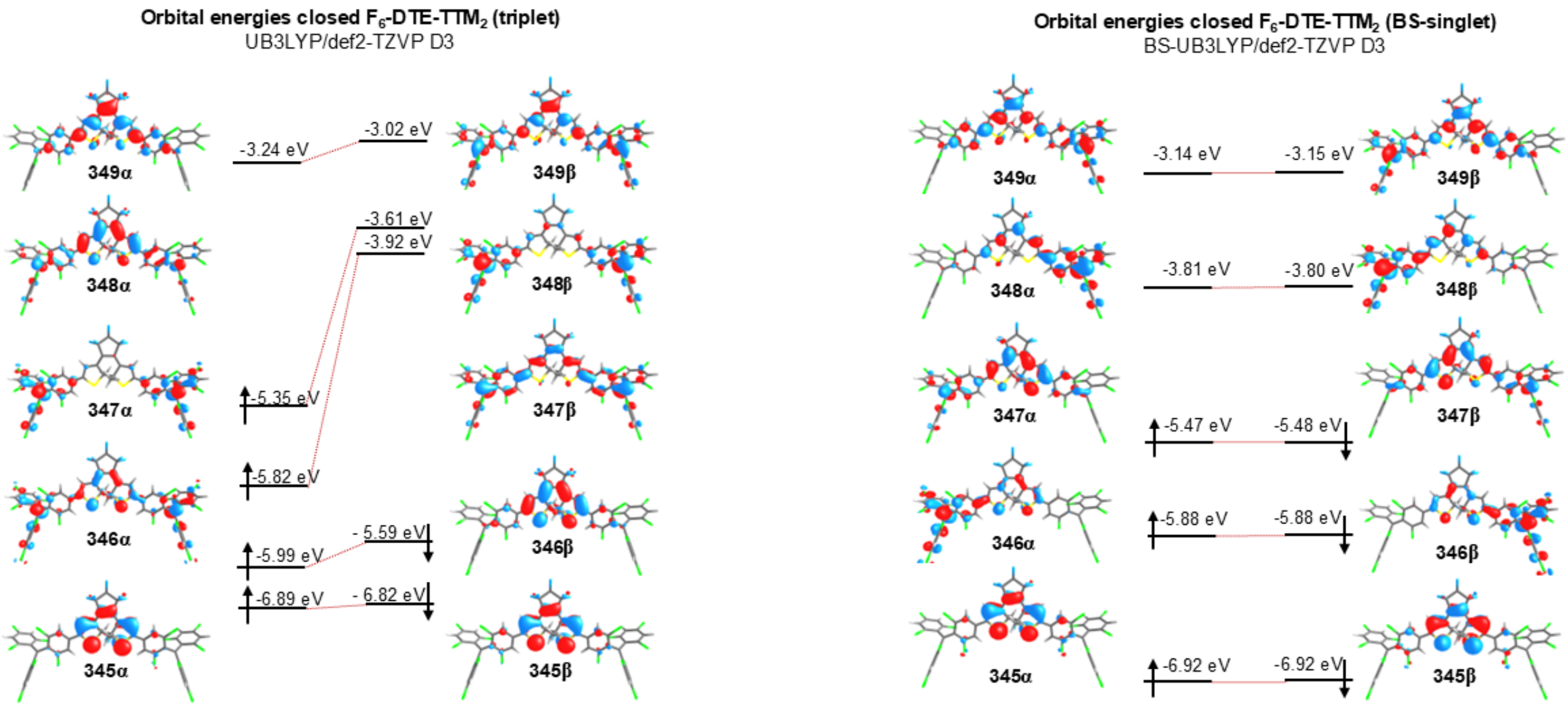

Figure S48 Orbital energies of **c-F$_6$-DTE-TTM$_2$** (triplet) and **c-F$_6$-DTE-TTM$_2$** (BS singlet) (*Geometry optimization and orbital analysis: UB3LYP/def2-TZVP D3, (isovalue: 0.03)*)

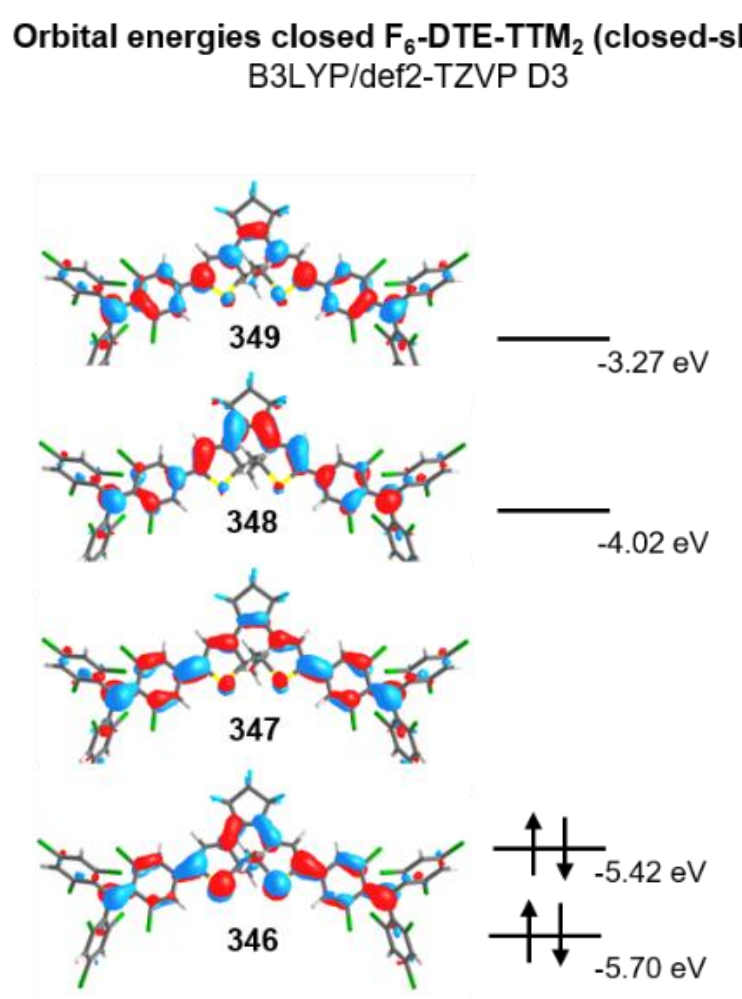

Figure S49 Orbital energies of **c-(F$_6$)-DTE-TTM$_2$** (closed-shell, quinoidal) (*Geometry optimization and orbital analysis: B3LYP/def2-TZVP D3, (isovalue: 0.03)*)

10. Comparison with diarylethene-nitronyl nitroxide spin couplers

To better compare the present system with the pioneering diarylethene-based spin couplers, we summarized the chemical structures in Figure S50 and key results in Table S4.

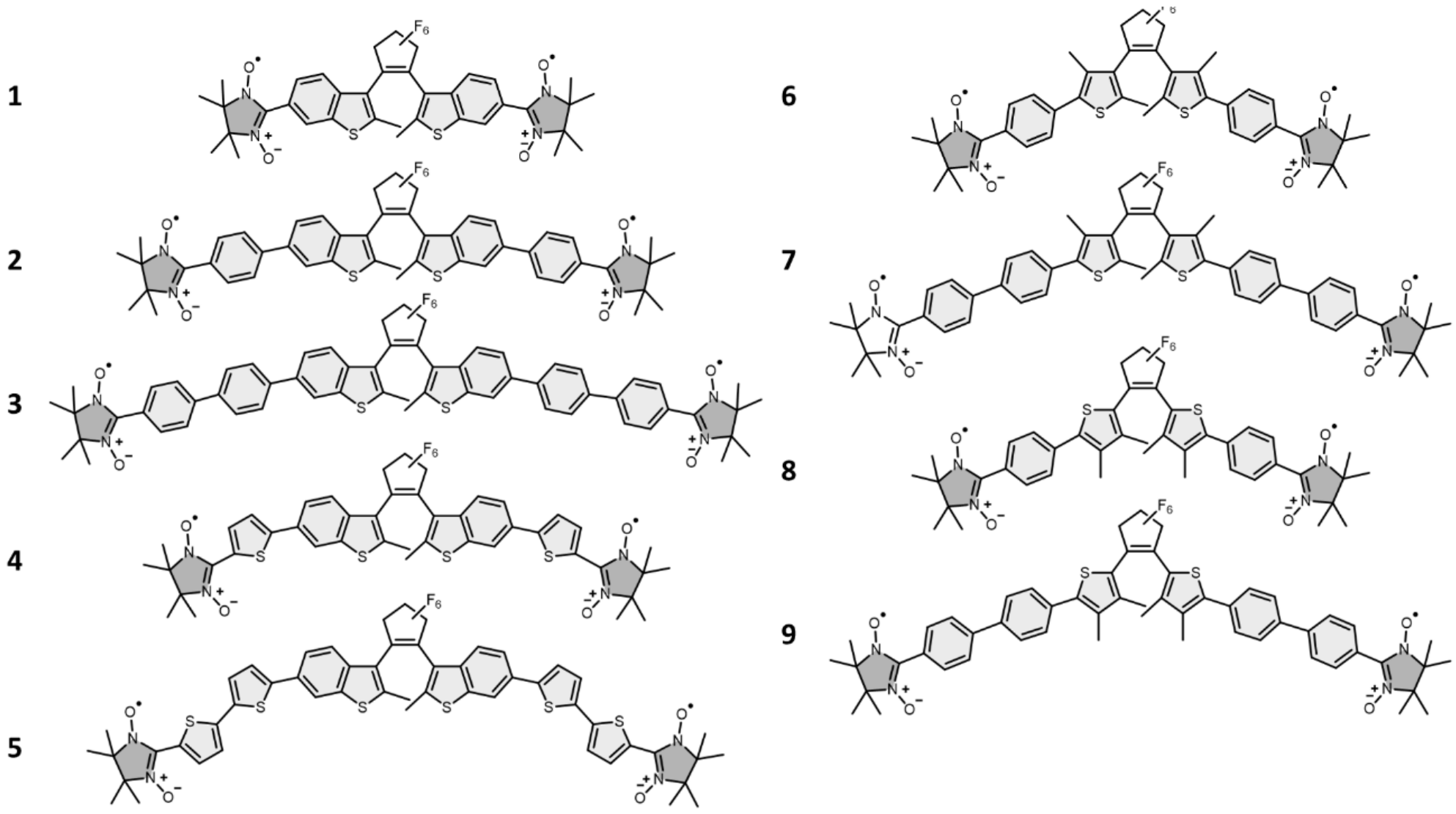

Figure S50 Chemical structures from Irie work[19-24]

Table S4 Comparison of early diarylethene-nitronyl nitroxide spin couplers and the present diradicaloids

Compound[1]	o→c / nm	c→o / nm	Magnetic parameter[2]/ K		Half-field transition	UV-Vis / nm		PL, PLQY, lifetime[3]	CV
			o	c		o	c		
1	313	517<br>578	-2.2	-11.6	Measured, but not observed for both o and c forms	309<br>377	280<br>348<br>400<br>565	n.r.	small orbital interaction between radical and diarylethene
2	313	517<br>578	~0.001	>0.08	n.r.	313<br>377	280<br>367<br>553	n.r.	n.r.
3	313	517	<0.0003	0.010	n.r.	320	311<br>374<br>548	n.r.	n.r.
4	366	578	0.0056;<br><0.0003	>0.04	n.r.	365	407<br>574	n.r.	n.r.
5	366	578	<0.00003	>0.04	n.r.	395	407<br>583	n.r.	n.r.
6	313	visible light	>0.04	>0.04	n.r.	314	350<br>588	n.r.	n.r.
7	366	visible light	<0.0003	>0.04	n.r.	319	350<br>583	n.r.	n.r.
8	–[4]	visible light	>0.04	>0.04	n.r.	310<br>375	360<br>469	n.r.	n.r.
9	–[4]	visible light	0.0032;<br><0.0003	<0.0003	n.r.	315<br>377	305<br>467	n.r.	n.r.
**DTE-TTM$_2$**	**365<br>430<br>465<br>490<br>525**	**heating**	**-5.6**	**-9.2**	**measured in both o and c forms**	**377<br>440<br>630**	**502<br>710<br>780**	**o:710 nm, 1.0%, 1.6 ns, c: n.d.**	weak interaction between radicals in o, but

**F_6-DTE-TTM_2**	**365<br>430<br>465<br>490<br>525**	**heating**	**-5.5**	**-9.7**	**377<br>430<br>607**	**520<br>710<br>790**	**o:710 nm, 0.7%, 1.7 ns<br>c: n.d.**	enhanced in c.

o = open form; c = closed form; n.r. = not reported; n.d. = not determined

[1]Compounds 1-9: *J. Am. Chem. Soc.* **2000**, *122*, 7195–7201; *J. Am. Chem. Soc.* **2000**, *122*, 8309–8310; *Chem. Eur. J.* **2001**, *7*, 3466–3473; *J. Org. Chem.* **2001**, *66*, 8799–8803; *J. Phys. Chem. B* **2002**, *106*, 11218–11225.

[2]For the Irie systems, the magnetic parameters are listed as originally reported ($2J/k_B$) or ($|2J/k_B|$) values. For DTE-TTM_2 and F_6-DTE-TTM_2, the values correspond to (ΔE_{ST}) obtained from VT-EPR fitting.

[3]Photoluminescence properties were not reported in Irie work.

[4]Light induced open-to-closed photoisomerization was not occurred in compounds 8 and 9.

11. Supplementary NMR and mass spectra

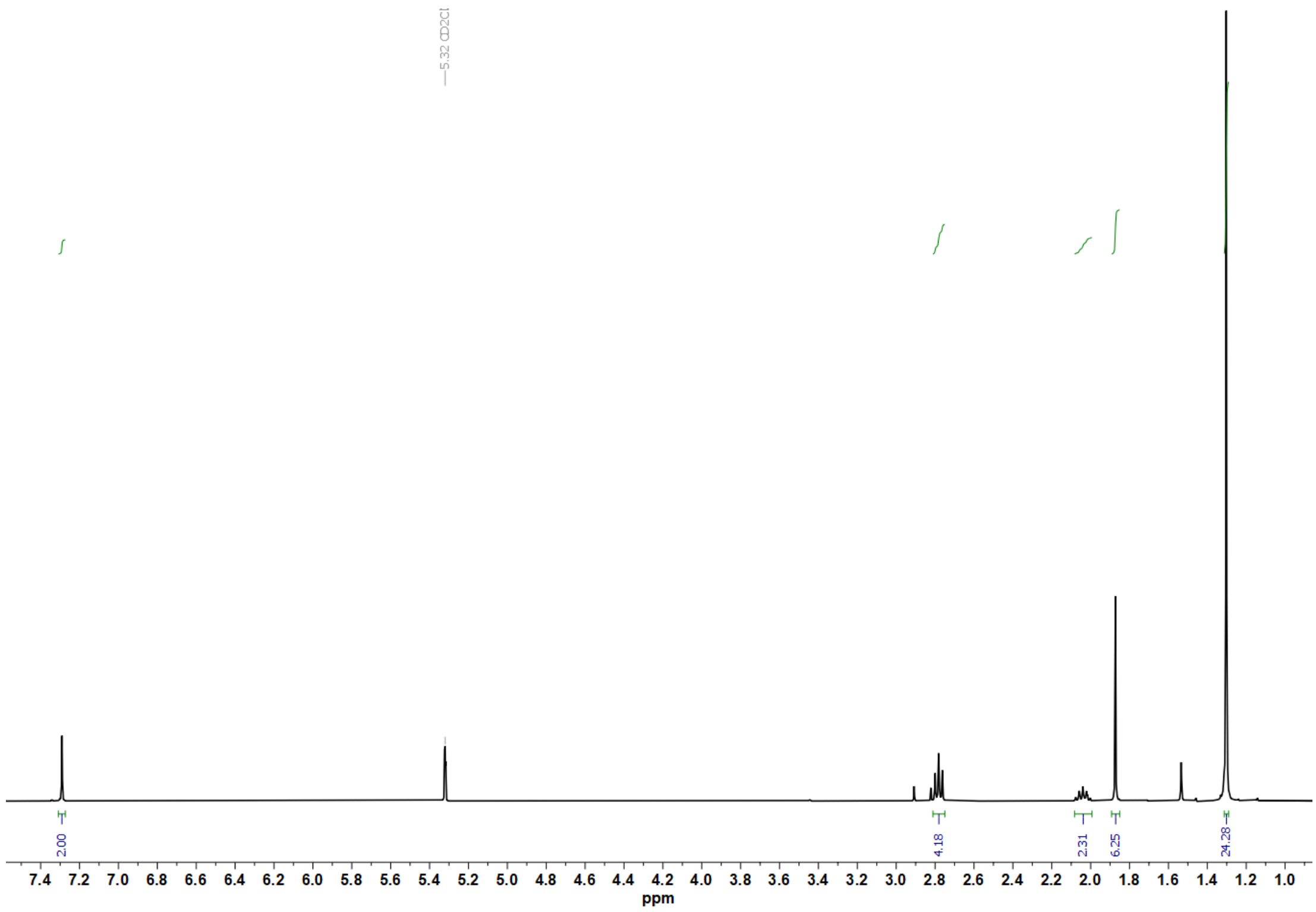

Figure S51 ^{1}H NMR of compound **o-DTE-Bpin$_2$** in CD_2Cl_2 (400 MHz, 298 K)

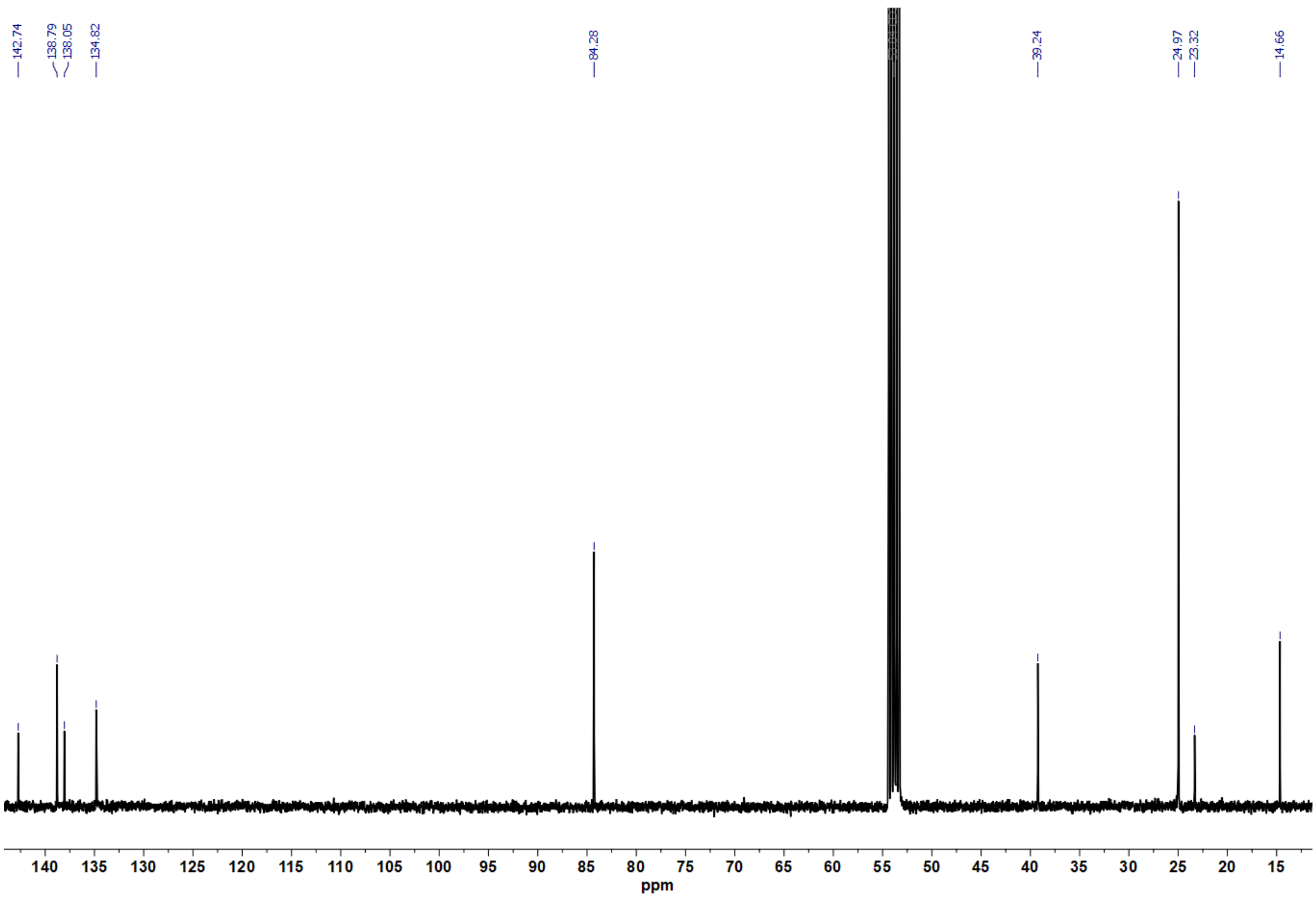

Figure S52 ^{13}C NMR of compound **o-DTE-Bpin$_2$** in CD_2Cl_2 (101 MHz, 298 K)

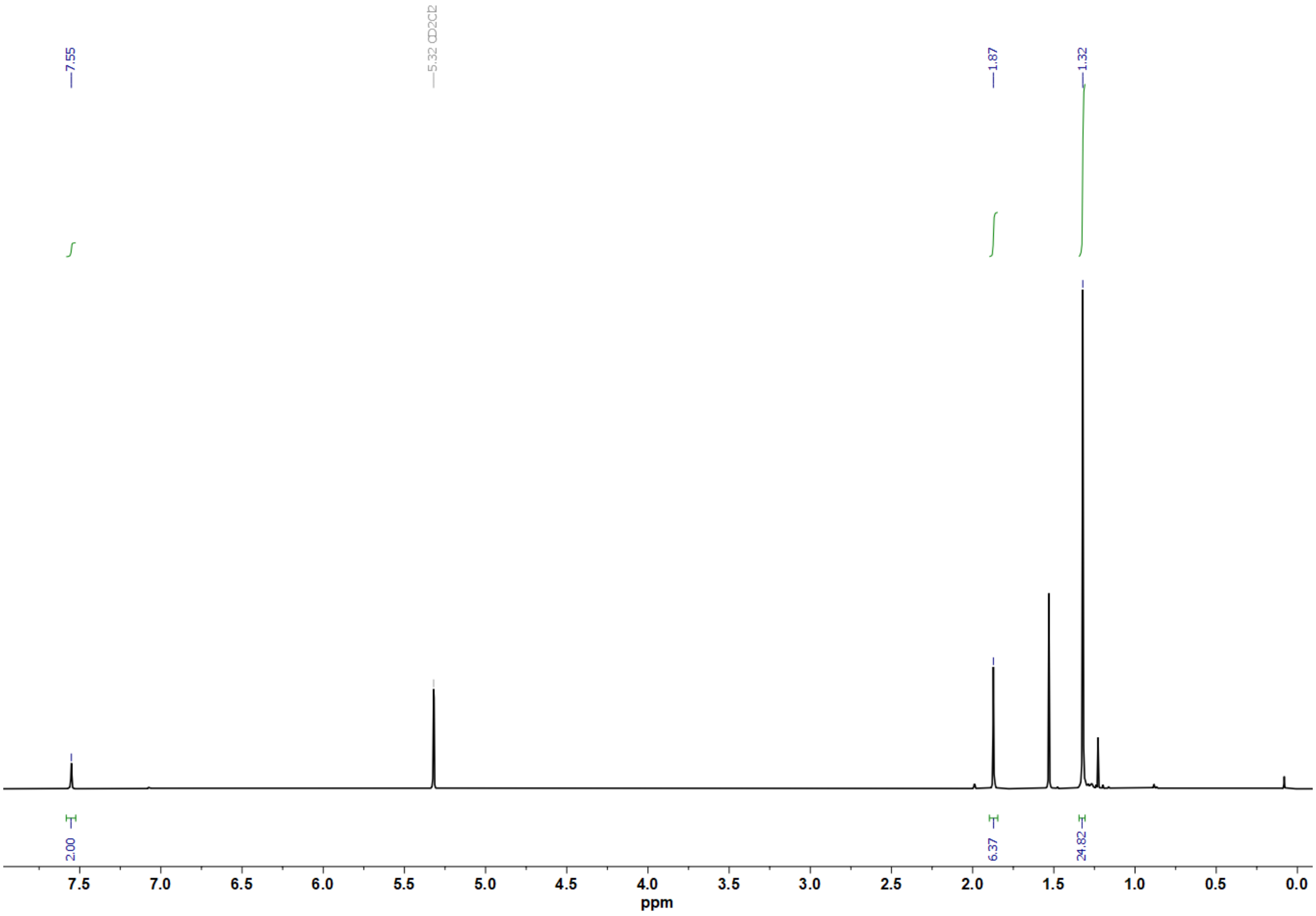

Figure S53 ^{1}H NMR of compound **o-F$_6$-DTE-Bpin$_2$** in CD_2Cl_2 (400 MHz, 298 K)

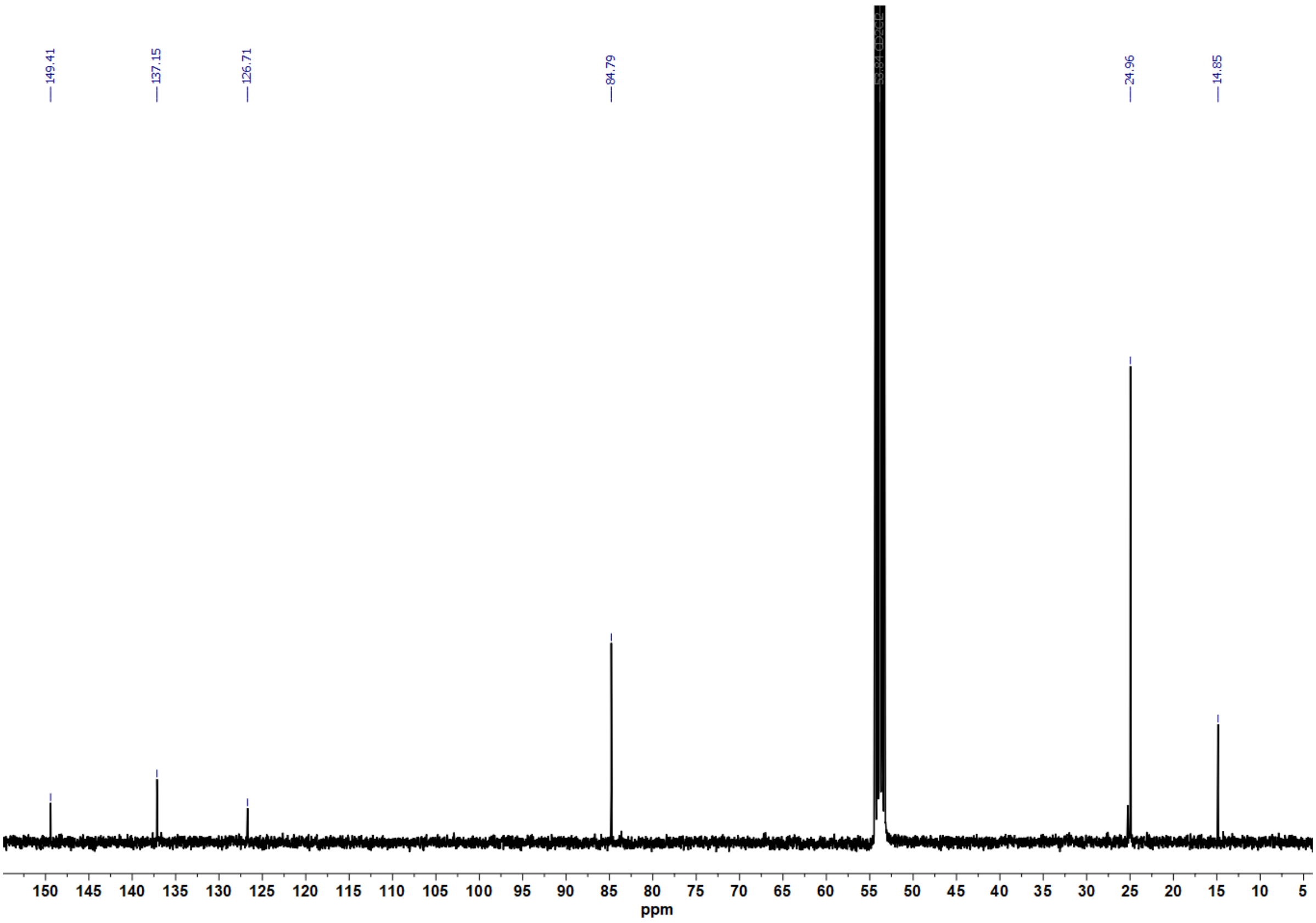

Figure S54 ^{13}C NMR of compound **o-F$_6$-DTE-Bpin$_2$** in CD_2Cl_2 (101 MHz, 298 K)

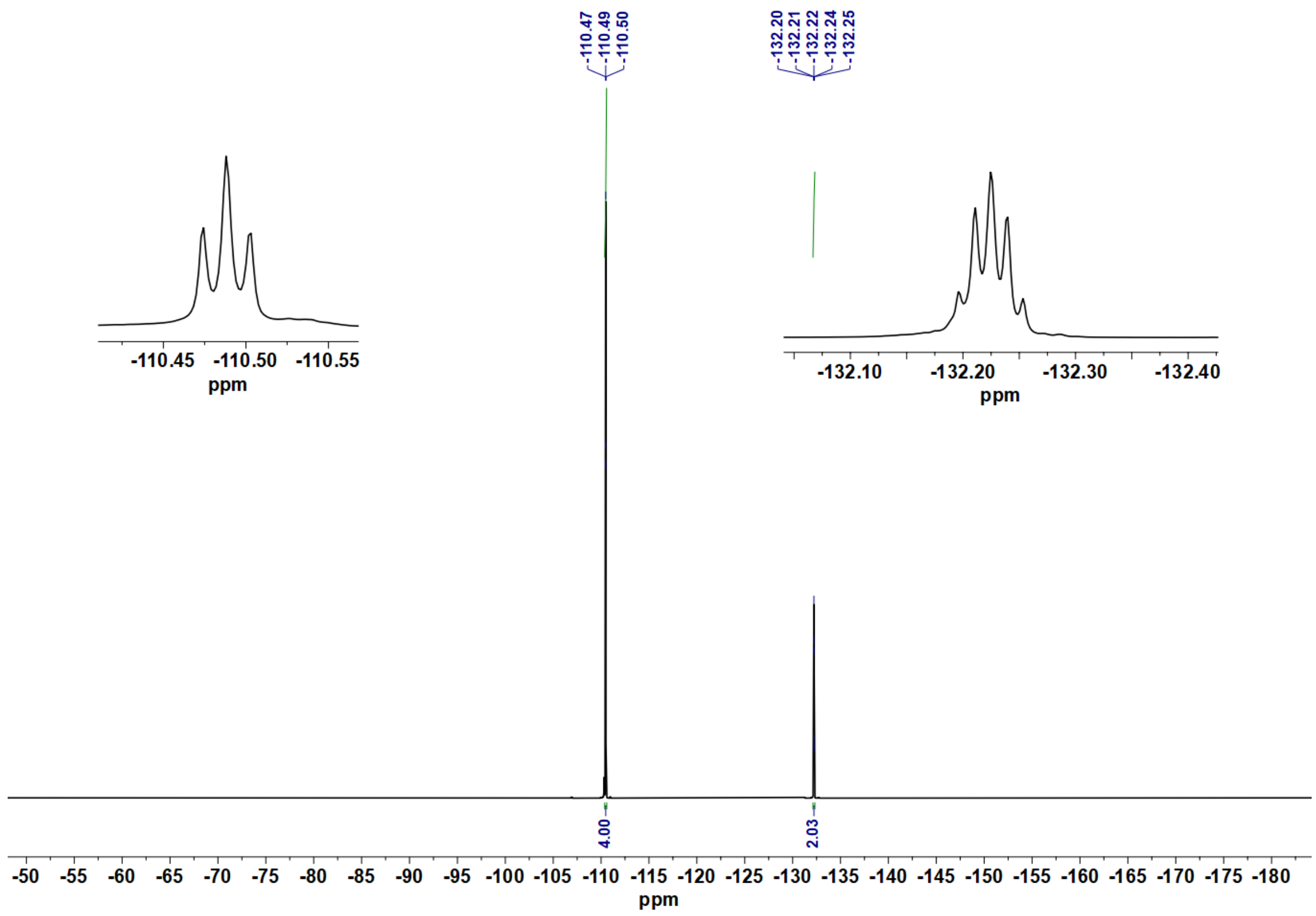

Figure S55 [13]F NMR of compound **o-F$_6$-DTE-Bpin$_2$** in CD_2Cl_2 (376 MHz, 298 K)

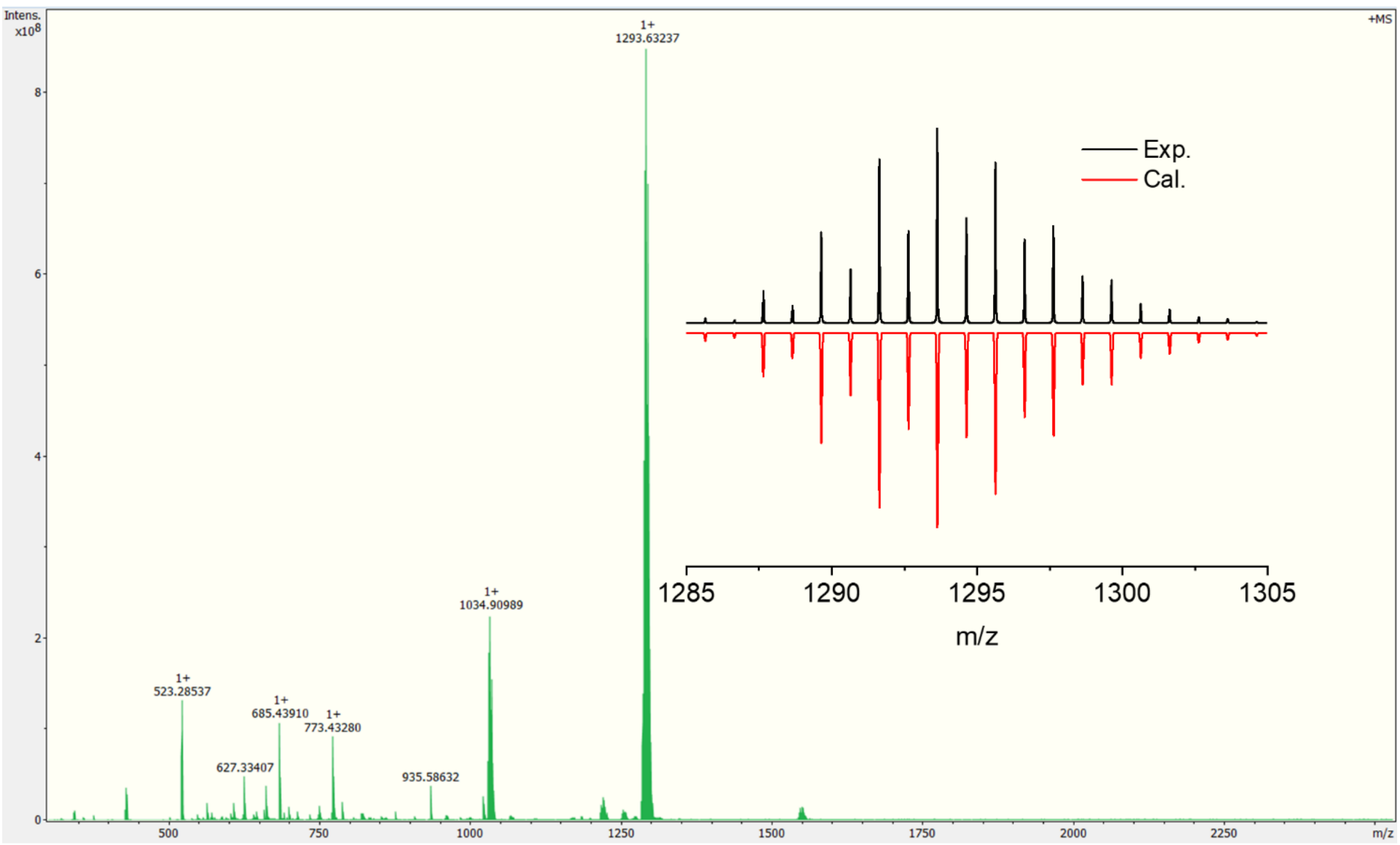

Figure S56 HR-MS (MALDI-MS; positive mode; Matrix: DCTB) of compound **o-DTE-TTM$_2$**, including isotopic patterns.

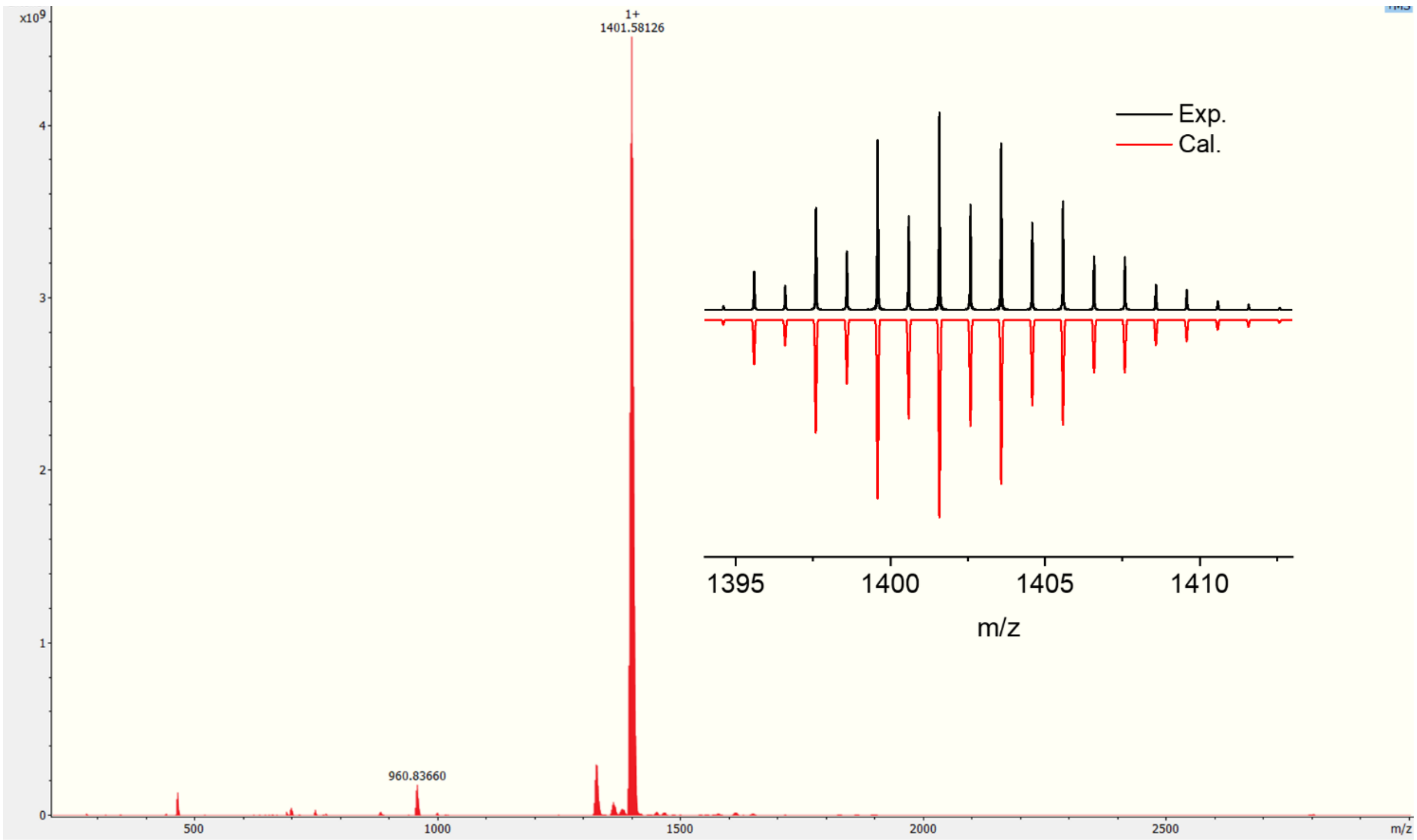

Figure S57 HR-MS (MALDI-MS; positive mode; Matrix: DCTB) of compound **o-F_6-DTE-TTM$_2$**, including isotopic patterns.

12. References

[1] R. Gostl, B. Kobin, L. Grubert, M. Patzel, S. Hecht, Sterically crowding the bridge of dithienylcyclopentenes for enhanced photoswitching performance, *Chem. Eur. J.* **2012**, *18*, 14282–14285.

[2] G. Sevez, J. L. Pozzo, Toward multi-addressable molecular systems: Efficient synthesis and photochromic performance of unsymmetrical bisthienylethenes, *Dyes Pigm.* **2011**, *89*, 246–253.

[3] M. E. Arnold, A. J. C. Kuehne, (2,6-Dichloro-4-iodophenyl)bis(2,4,6-trichlorophenyl)methane as a precursor in efficient cross-coupling reactions for donor and acceptor functionalized triphenylmethyl radicals, *Dyes Pigm.* **2023**, *208*, 110863.

[4] O. V. Dolomanov, L. J. Bourhis, R. J. Gildea, J. A. K. Howard, H. Puschmann, *OLEX2*: a complete structure solution, refinement and analysis program, *J. Appl. Crystallogr.* **2009**, *42*, 339–341.

[5] G. M. Sheldrick, A short history of SHELX, *Acta Crystallogr. A* **2008**, *64*, 112–122.

[6] G. M. Sheldrick, Crystal structure refinement with SHELXL, *Acta Crystallogr. C* **2015**, *71*, 3–8.

[7] J. J. Snellenburg, S. Laptenok, R. Seger, K. M. Mullen, I. H. M. van Stokkum, Glotaran: A Java-Based Graphical User Interface for the R Package TIMP, *Journal of Statistical Software* **2012**, *49*, 1 – 22.

[8] F. Neese, Software update: The ORCA program system-version 6.0, *WIREs Comput. Mol. Sci.* **2025**, *15*, e70019.

[9] S. Grimme, J. Antony, S. Ehrlich, H. Krieg, A consistent and accurate ab initio parametrization of density functional dispersion correction (DFT-D) for the 94 elements H-Pu, *J. Chem. Phys.* **2010**, *132*, 154104.

[10] U. Ekstrom, L. Visscher, R. Bast, A. J. Thorvaldsen, K. Ruud, Arbitrary-order density functional response theory from automatic differentiation, *J. Chem. Theory. Comput.* **2010**, *6*, 1971–1980.

[11] S. Grimme, S. Ehrlich, L. Goerigk, Effect of the damping function in dispersion corrected density functional theory, *J. Comput. Chem.* **2011**, *32*, 1456–1465.

[12] F. Weigend, Accurate coulomb-fitting basis sets for h to rn, *Phys. Chem. Chem. Phys.* **2006**, *8*, 1057–1065.

[13] F. Weigend, R. Ahlrichs, Balanced basis sets of split valence, triple zeta valence and quadruple zeta valence quality for H to Rn: Design and assessment of accuracy, *Phys. Chem. Chem. Phys.* **2005**, *7*, 3297–3305.

[14] K. Yamaguchi, Y. Takahara, T. Fueno, in *Appl. Quantum Chem.*, **1986**, pp. 155–184.

[15] T. Soda, Y. Kitagawa, T. Onishi, Y. Takano, Y. Shigeta, H. Nagao, Y. Yoshioka, K. Yamaguchi, Ab initio computations of effective exchange integrals for H-H, H-He-H and Mn_2O_2 complex:: comparison of broken-symmetry approaches, *Chem. Phys. Lett.* **2000**, *319*, 223–230.

[16] K. Yamaguchi, The electronic structures of biradicals in the unrestricted Hartree-Fock approximation, *Chem. Phys. Lett.* **1975**, *33*, 330–335.

[17] A. Punzi, Y. Dai, C. N. Dibenedetto, E. Mesto, E. Schingaro, T. Ullrich, M. Striccoli, D. M. Guldi, F. Negri, G. M. Farinola, D. Blasi, Dark state of the Thiele hydrocarbon: Efficient solvatochromic emission from a nonpolar centrosymmetric singlet diradicaloid, *J. Am. Chem. Soc.* **2023**, *145*, 20229–20241.

[18] S. Canola, J. Casado, F. Negri, The double exciton state of conjugated chromophores with strong diradical character: insights from TDDFT calculations, *Phys. Chem. Chem. Phys.* **2018**, *20*, 24227–24238.

[19] K. Matsuda, M. Irie, Photoswitching of intramolecular magnetic interaction using a photochromic spin coupler: An ESR study, *J. Am. Chem. Soc.* **2000**, *122*, 8309–8310.

[20] K. Matsuda, M. Irie, Photoswitching of magnetic interaction: diarylethene photochromic spin couplers, *Polyhedron* **2001**, *20*, 1391–1395.

[21] K. Matsuda, M. Irie, Photochromism of diarylethenes with two nitronyl nitroxides: photoswitching of an intramolecular magnetic interaction, *Chem. Eur. J.* **2001**, *7*, 3466–3473.

[22] K. Matsuda, M. Matsuo, M. Irie, Photoswitching of intramolecular magnetic interaction using diarylethene with oligothiophene pi-conjugated chain, *J. Org. Chem.* **2001**, *66*, 8799–8803.

[23] K. Matsuda, M. Matsuo, S. Mizoguti, K. Higashiguchi, M. Irie, Reversed photoswitching of intramolecular magnetic interaction using a photochromic bis(2-thienyl)ethene spin coupler, *J. Phys. Chem. B* **2002**, *106*, 11218–11225.

[24] K. Matsuda, M. Irie, A diarylethene with two nitronyl nitroxides: Photoswitching of intramolecular magnetic interaction, *J. Am. Chem. Soc.* **2000**, *122*, 7195–7201.
